# Amplicon-Based Profiling of Fungal Communities in Decayed Outdoor Wood Structures and Natural Logs in Tropical Singapore

**DOI:** 10.3390/jof12100754

**Published:** 2026-10-08

**Authors:** Sng Yu En Jerrica, Kelly Loke, Shi Hui Beh, Ying Chua, Yujia Deng, Yan Hong

**Affiliations:** 1 School of Biological Sciences, Nanyang Technological University, 60 Nanyang Drive, Singapore 637551, Singapore; sngy0016@e.ntu.edu.sg (S.Y.E.J.); kloke004@e.ntu.edu.sg (K.L.); sbeh003@e.ntu.edu.sg (S.H.B.); chua1069@e.ntu.edu.sg (Y.C.); 2 School of Life Sciences, Nanjing Agricultural University, Nanjing 210095, China; 9231310206@stu.njau.edu.cn

**Keywords:** wood biodeterioration, outdoor wood structures, wood-decay fungi, ITS1 amplicon sequencing, fungal community composition, tropical Singapore, *Trichaptum byssogenum*, *Paecilomyces maximus*, *Schizophyllum commune*, *Phanerochaete* sp.

## Abstract

Wood structures (processed wood) are increasingly incorporated into Singapore’s sustainable built environment, but their durability is challenged by biodeterioration exacerbated by the tropical climate. We collected 133 decayed wood samples from outdoor wood structures and natural logs at 15 sites including public parks, wooden buildings, and private houses. Fungal communities were characterized using ITS1 amplicon sequencing, with taxonomic assignments initially performed using the UNITE database and subsequently refined using the ITS RefSeq Targeted Loci database, reducing inconclusive genus-level identifications from 55.8% to 44.0%. All but one site exhibited substantial local-scale heterogeneity in fungal communities, with between-site Bray–Curtis dissimilarities ranging from 0.61 to 1.0, demonstrating marked compositional differences among sampling sites, with median within-site Bray–Curtis dissimilarities ranging from 0.60 to 0.98, and samples collected from the same pieces of processed wood or natural log commonly harboring distinct fungal communities. The sole exception was a residential site dominated by *Trichaptum byssogenum.* PERMANOVA indicated that sampling location and substrate type explained 25.1% and 4.6% of the variation in fungal community composition, respectively, suggesting weak influence of wood treatment. One ascomycete and two basidiomycete isolates were further evaluated for their wood-decaying capacity in vitro. Remarkably, the ascomycete *Paecilomyces maximus* alone was capable of degrading angsana, balau, spruce, and rubberwood blocks, highlighting the potentially important role of ascomycetes as active primary saprotrophs in wood decomposition, consistent with their dominant presence in our study (eight of the nine widely dominant genera present in four–seven sites). Collectively, these findings reveal the complexity and heterogeneity of tropical wood biodeterioration and provide insights relevant to wood processing, durability assessment, and the management of timber structures in tropical environments.

## 1. Introduction

The “Singapore Green Plan 2030” is a national movement to transform Singapore into a greener and more sustainable city [1]. As part of this initiative, there is an increasing demand for wood material for wood infrastructure such as wood setups outdoors (benches, tables, wood walks, handrails and structures for playgrounds), furniture and flooring indoors [2], and buildings using mass-engineered timber (MET).

MET is produced from logs harvested from sustainably managed plantations of softwood species, such as Norway Spruce (*Picea abies* (L.) H. Karst.) and Sitka Spruce (*Picea sitchensis* (Bong.) Carrière), of which the wood possesses a favorable strength-to-weight ratio and good dimensional stability. MET-based buildings have a lower carbon footprint and net carbon emissions than traditional steel- or concrete-based buildings [3]. Consequently, increasing efforts have been made to expand the use of MET in infrastructure projects, including at one of our sampling sites, GAIA, the new home of Nanyang Business School and one of Asia’s largest wooden buildings, as well as in public spaces such as the Jurong Lake Gardens’ pavilions and the upcoming Punggol Digital District.

Parks and residential areas are also making greater use of processed wood-based structures, including handrails, poles, fences, tables and benches, due to their sustainable supply, low carbon emissions, and role as carbon sinks. Common timber materials used in Singapore’s outdoor infrastructure include balau, a dense and durable tropical hardwood obtained from several *Shorea* Roxb. ex C.F. Gaertn species, and rubberwood, derived from *Hevea brasiliensis* (Willd. ex A. Juss.) Müll. Arg. [4,5].

Wood is widely regarded as a sustainable and renewable construction material because of its favorable strength-to-weight ratio [6,7]. However, as a natural carbon-rich substrate, it is also inherently susceptible to biological degradation. Wood decay fungi play a central role in decomposing dead plant material and recycling carbon and nutrients within ecosystems, making them key drivers of both nutrient cycling and wood decomposition.

Singapore is located in the humid equatorial tropics (1°17′ N, 103°51′ E) and is characterized by consistent high temperatures, high relative humidity, and abundant year-round rainfall, with mean annual temperatures of 27.5–28.0 °C, a relative humidity of 82–84%, approximately 170–190 rainy days annually, and an annual rainfall of 2200–2600 mm [8]. Singapore’s high relative humidity and temperature provide a conducive environment for fungal colonization and growth [9].

**Figure 1:**
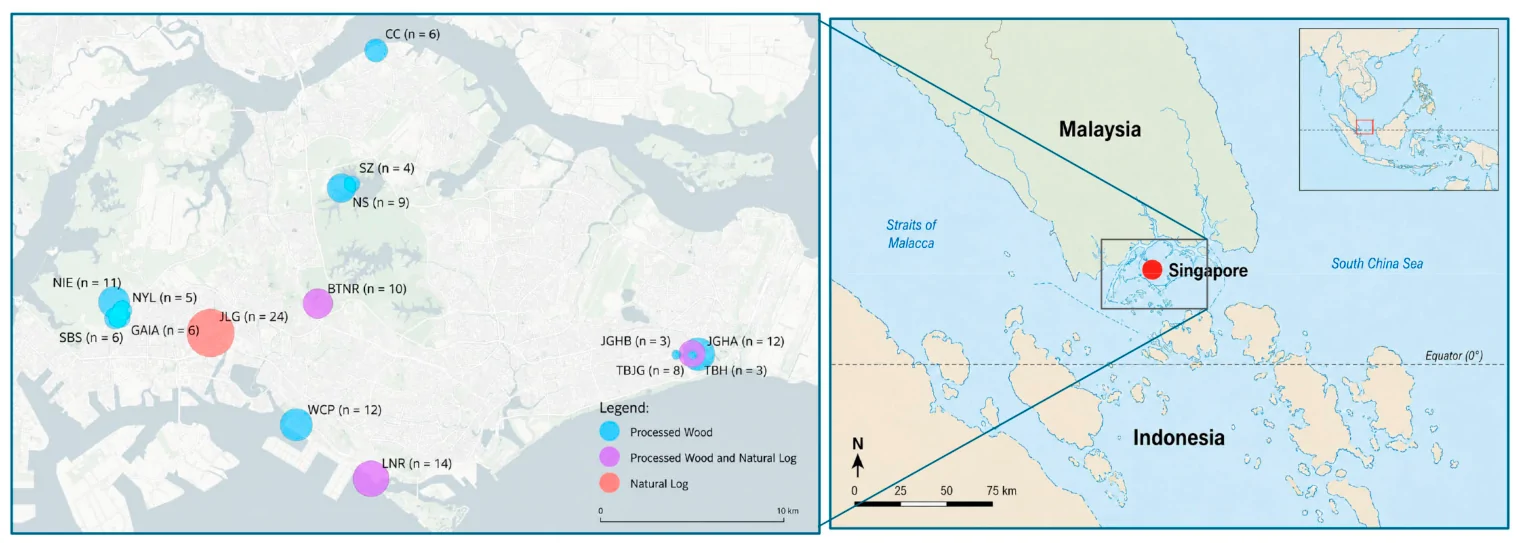
Map of Singapore showing the 15 sampling sites. Circle size is proportional to the number of samples collected at each site (n = 3–24).

**Figure 2:**
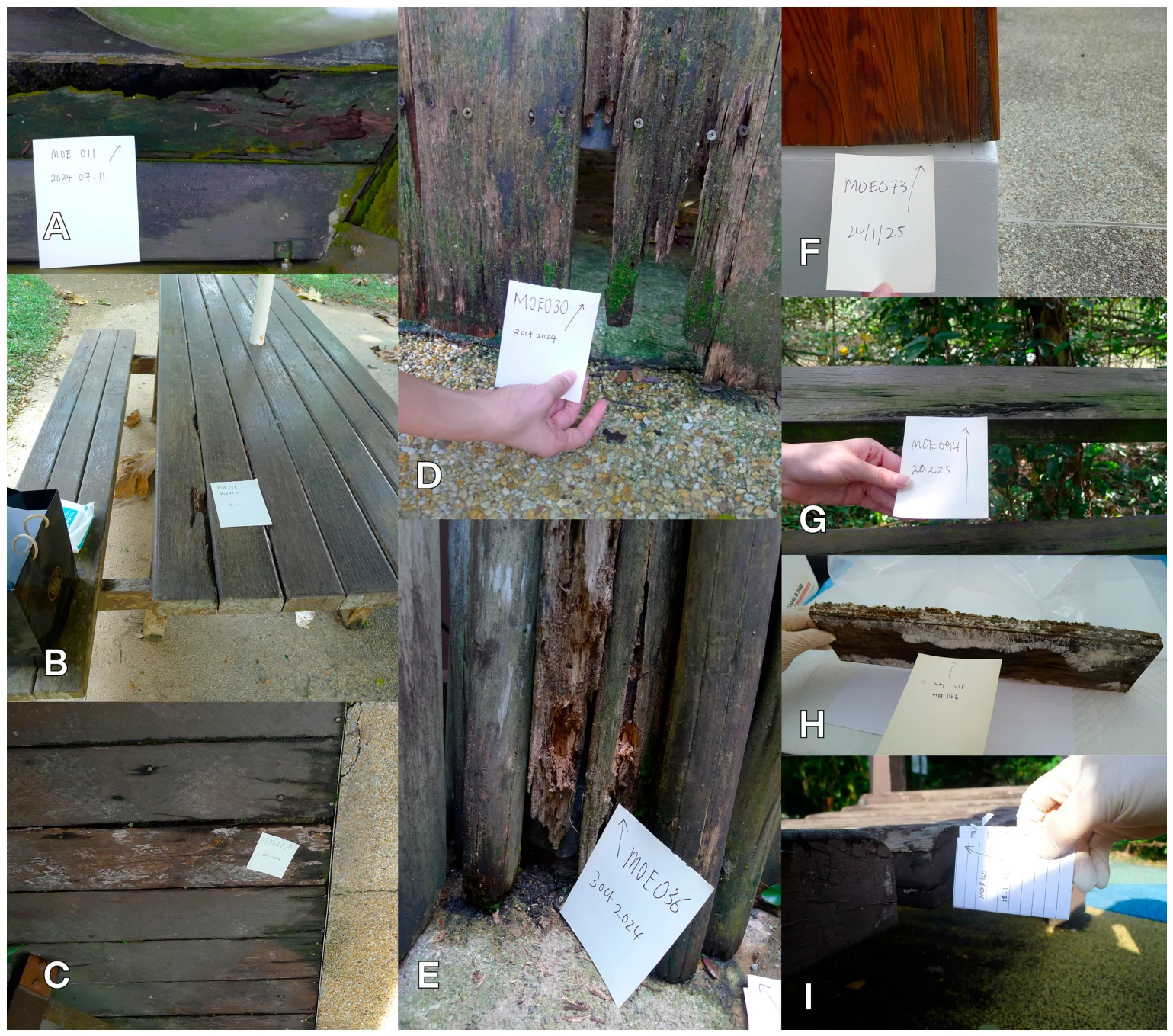
Representative processed wood samples collected in this study. (**A**) MOE011 (SBS1.2): wooden crate box located in the open area on Level 2 of the School of Biological Sciences (SBS); (**B**) MOE028 (NIE8.1): picnic table in the open area on Level 1 of the National Institute of Education (NIE); (**C**) MOE031 (SZ2.1): wooden floor planks at the Singapore Zoo (SZ); (**D**) MOE030 (SZ1.1): wooden door planks at SZ; (**E**) MOE036: wooden poles at SZ; (**F**) MOE073 (GAIA4.1): wooden square pillar at GAIA, Nanyang Technological University (GAIA); (**G**) MOE094 (LNR11.2): handrail at Labrador Nature Reserve (LNR); (**H**) MOE146 (JGHA11.1): wooden plank collected from Jalan Greja House-A (JGHA); and (**I**) MOE160 (TBJG1.2): wooden playground structure at Taman Bedok–Jalan Greja neighborhood (TBJG).

Although considerable attention has been given to pathogenic fungi affecting urban trees [10], little is known about fungal communities associated with the degradation of natural deadwood and outdoor wooden structures. Biodeterioration of wooden structures can result in progressive structural damage, ultimately necessitating costly repair or replacement [11]. Such deterioration has been reported at sites in Singapore (Figure 1), including Jalan Greja House A (JGHA) (Figure 2H) and the Singapore Zoo (Figure 2C–E), where the warm, high-moisture and humid lowland tropical environment may promote soft-rot fungus growth [12,13]. It remains unclear whether wood biodeterioration in Singapore is driven by a limited number of dominant fungal species, and whether the same fungal taxa are involved in the decay of natural deadwood (untreated fallen or cut logs) and processed wood (chemically and/or physically treated wood used in built environments and outdoor structures). Addressing this knowledge gap may be important for the more effective and targeted prevention and mitigation of wood deterioration.

Filamentous fungi possess adaptations that make them the dominant agents of deadwood decomposition, even though deadwood, in general, though abundant with high carbon content, can be considered nutrient-imbalanced due to its deficiency in essential elements such as nitrogen and phosphorous [14,15]. Furthermore, parts of the deadwood may be protected by a recalcitrant lignin barrier that makes it difficult for many microorganisms to access the polysaccharides as a nutrient source [14], making it hard to digest [15]. However, fungi are capable of degrading lignocellulosic materials, thereby releasing carbon stored in wood and contributing to nutrient cycling that helps maintain ecosystem balance [16]. Wood-decay fungi are commonly grouped according to the major wood biopolymers they degrade and are generally divided into three categories: (i) white-rot fungi, primarily members of *Basidiomycota* R.T. Moore, degrade all major wood components, including cellulose, hemicellulose, and lignin, leaving behind whitish fibrous residues; (ii) brown-rot fungi, also mainly members of *Basidiomycota*, selectively degrade cellulose and hemicellulose while leaving behind modified brown lignin [14,17,18]; and (iii) soft-rot fungi, primarily members of *Ascomycota* Caval.-Sm, degrade cellulose and hemicellulose and, to a lesser extent, lignin, and are characterized by tapering cavities within wood cell walls and a brownish-gray, spongy residual structure [11,14]. Most known wood-decay fungi belong to the phylum *Basidiomycota*, whereas a smaller proportion are classified as *Ascomycota* [19]. Historically, fungi within *Basidiomycota* have been regarded as the primary drivers of wood decay because many white-rot species are capable of completely degrading lignin to CO_2_ and H_2_O, thereby enabling access to cellulose that is otherwise protected by lignin [20]. In contrast, many ascomycetes G. Winter have traditionally been considered incapable of extensive lignin degradation and are typically associated with soft-rot decay, which primarily affects the outer layers of wood and modifies wood structure without completely removing lignin [14,21]. However, an experimental study on higher ascomycete fungi showed that several species caused substantial mass loss in birch (*Betula* sp.) wood in the absence of basidiomycetes G. Winter and were capable of independently degrading syringylpropane lignin units [22]. This finding raises the possibility that fungi within *Ascomycota* may also function as primary wood decomposers. Most investigations of decay fungi in timber construction concern indoor damage, with only a few examining exterior timbers in the Northern hemisphere and Antarctic Peninsular and there being a lack of systematic surveys of wood-decay fungi in the tropics [21]. Their role in outdoor wood structure biodeterioration, particularly in tropical environments such as Singapore, remains poorly understood.

Traditional fungal identification methods rely heavily on culture-dependent approaches, which tend to underestimate fungal diversity and bias results toward species that are readily culturable on standard growth media [23]. Although culture-based methods enable the isolation of pure fungal strains for downstream analyses and confirm the presence of viable fungi, they remain limited because many fungal species are unculturable [24,25]. It has been estimated that only approximately 17% of fungal species have been successfully cultured, as many depend on obligate interspecies or mutualistic interactions for growth [26,27]. Consistent with this, only a subset of the dominant fungi detected in our previous metagenomics analysis of tree decay could be isolated in culture, and several of these isolates exhibited slow growth, further highlighting the limitations of culture-dependent approaches when used alone [10]. In comparison, ITS amplicon sequencing analysis provides a more comprehensive and less biased characterization of fungal communities and their relative abundances in decaying wood [26].

This study aimed to characterize fungal communities associated with decaying outdoor wooden structures across buildings, parks, and residential areas in Singapore, in comparison with those associated with natural log decay. Particular emphasis was placed on identifying dominant fungal taxa, assessing mycobiome similarity in relation to geographic distance and substrate type (wood structure vs. natural log), and possible associations of *Basidiomycota* and *Ascomycota* with wood decay. In addition, culture-dependent methods were used to obtain some pure fungal isolates and evaluate their ability to degrade in vitro the wood blocks of four locally relevant species: angsana (*Pterocarpus indicus* Willd.), spruce (*Picea* sp.), rubberwood (*Hevea brasiliensis*), and balau (*Shorea* sp.). This study provides a deeper understanding of the fungal taxa possibly driving wood decay under tropical climatic conditions, which will help evaluate the risks of structural biodeterioration and support the development of effective prevention and mitigation strategies, particularly in tropical countries that are rapidly expanding the use of timber-based infrastructure.

## 2. Materials and Methods

### 2.1. Sample Collection and Study Area

Singapore has a tropical rainforest climate (Köppen climate classification: Af) characterized by uniformly high temperatures, high humidity, and substantial year-round precipitation averaging approximately 2190 mm annually [27]. Wind conditions are generally mild, with mean wind speeds below 2.5 m/s, predominantly originating from the northeast and south. Singapore experiences two monsoon seasons, the northeast monsoon (December to early March) and the southwest monsoon (June to September), both of which can bring short periods of heavy rainfall and strong winds exceeding 10 m/s [28].

During field collection, each decayed wood sample was assigned a unique identifier beginning with “MOE010” and a location-based code consisting of up to four letters representing the site initials followed by a numerical designation (e.g., SZ1 for a sample collected from the Singapore Zoo). Where present, fruiting bodies attached to or immediately adjacent to decay wood samples were also collected. Samples collected from different regions of the same wood piece were distinguished using decimal extensions (e.g., SZ1.1 and SZ1.2).

Two samples (NIE1.1 and NIE2.1) were treated as technical duplicates because they were inadvertently mixed during DNA extraction. The two equal aliquots of the mixture were retained to evaluate the reproducibility and reliability of the ITS amplicon sequencing analysis.

**Figure 3:**
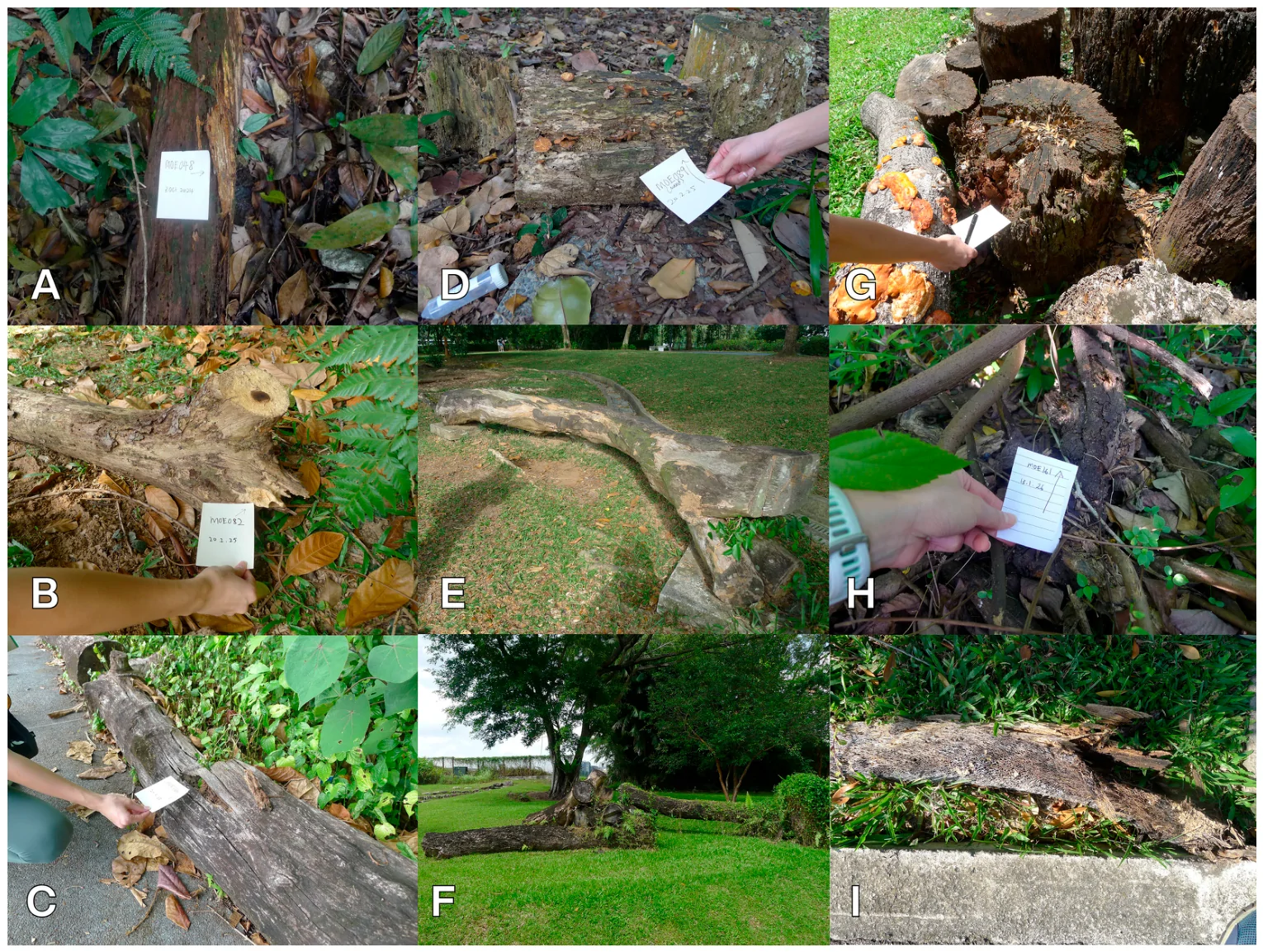
Representative natural log samples were collected in this study. (**A**) MOE048 (BTNR1.1): fallen log at Bukit Timah Nature Reserve (BTN); (**B**) MOE082 (LNR1.1): fallen log at Labrador Nature Reserve (LNR); (**C**) MOE086 (LNR5.1): placed logs along a walkway at LNR; (**D**) MOE089 (LNR7.2): tree stump at LNR; (**E**) MOE092 (LNR10.1): a tree log bench at LNR; (**F**) MOE108 (JLG6.1) and MOE109 (JLG7.1): placed logs at Jurong Lake Gardens (JLG); (**G**) MOE117 (JLG13.1): placed tree stumps at JLG; (**H**) MOE161 (TBJG2.1): naturally decaying wood from a plant at the Taman Bedok–Jalan Greja neighborhood (TBJG); and (**I**) MOE169 (TBJG9.2): a fallen log at TBJG.

A total of 133 wood samples were collected from 15 study sites across Singapore (Table 1, Figure 1) and grouped into two substrate categories: processed wood and natural logs. Processed wood refers to timber that has been chemically and/or physically treated and incorporated into built structures such as flooring, railings, and furniture. Figure 2 and Figure 3 illustrate representative processed wood and natural log samples collected in this study.

Both substrate types were sampled at three locations—Bukit Timah Nature Reserve, Labrador Nature Reserve, and Taman Bedok–Jalan Greja—to enable comparative analysis of the mycobiomes associated with the two forms of decaying wood within the same locations.

Information on timber origin, wood species, treatment history, installation date, and replacement history was unavailable for many processed wood structures. Hence, these variables were not included in the analyses.

Sampling sites encompassed diverse environments, including nature reserves and public parks managed by the National Parks Board, zoological facilities managed by Mandai Wildlife Group, campus areas within Nanyang Technological University, residential neighborhoods, and private residences.

Figure 1 provides a map showing the locations of all 15 sampling sites across Singapore.

Decayed samples were selected based on visible structural damage or the presence of fungal fruiting bodies. Decaying wood material (~1 g) was scraped using a chisel and transferred into sterile 50 mL centrifuge tubes (Bio Basic Asia Pacific Pte. Ltd., Singapore, Singapore). Sampling tools were disinfected with 70% ethanol between collections to minimize cross-contamination. For each sample, the collection site, observable decay characteristics (including color, texture, and extent of decay), and any additional notes provided by collaborating organizations were recorded.

### 2.2. DNA Extraction

Wood samples and fruiting bodies were cryogenically ground, and approximately 0.15 g of ground material was processed using the PowerLyzer PowerSoil DNA Isolation Kit (QIAGEN LLC, Germantown, MD, USA) with minor protocol modifications. Solutions C1 and C6 were pre-incubated at 60 °C to improve DNA yield. Samples were then either subjected to three cycles of 10 s vortexing followed by 5 min incubation at 70 °C, with a final 20 s vortexing step, or pulverized at 35 Hz for 5 min using an AM100S HF Vibratory Ball Mill (Ants Scientific Instruments, Beijing, China) in place of the PowerLyzer 24 Homogenizer (QIAGEN).

DNA from pure fungal cultures was extracted using a modified Lyticase–Chelex-100 protocol adapted from Pryce et al. [29]. Approximately 2–4 mm^2^ of mycelial biomass, together with zirconia–silica beads, was suspended in 500 µL of 10 mM Tris–HCl (pH 8.0) and pulverized at 35 Hz for 5 min using an AM100S HF Vibratory Ball Mill. Samples were subsequently treated with 30 U of Lyticase (Sigma-Aldrich, St. Louis, MO, USA) at 37 °C for 45 min, followed by incubation with 20 mg of Chelex^®^-100 (Bio-Rad Laboratories, Hercules, CA, USA) at 95 °C for 10 min. Samples were then cooled, centrifuged at 13,000× *g* for 2 min, and purified using the EZ-10 Spin Column PCR Products Purification Kit (Bio Basic Asia Pacific Pte. Ltd., Singapore, Singapore) with minor modifications: the Elution Buffer was pre-incubated at 60 °C, followed by a final 15 min incubation at 60 °C to improve DNA yield. DNA concentration and purity were assessed using a NanoDrop 1000 spectrophotometer (Thermo Scientific, Waltham, MA, USA), and DNA integrity was verified by agarose gel electrophoresis.

### 2.3. Amplicon Sequencing and Bioinformatic Analysis

Genomic DNA extracted from decayed wood samples was submitted to Novogene for ITS1-targeted amplicon sequencing. DNA was fragmented to 250–300 bp for the construction of 350 bp insert libraries and sequenced using a paired-end 150 bp next-generation sequencing strategy. Each sample yielded more than 50,000 raw reads, with most samples exceeding 100,000 reads. Following quality control and denoising, reads were resolved into amplicon sequence variants (ASVs), and taxonomic assignments were performed against the UNITE reference database using the QIIME2 Naïve Bayes classifier. Taxonomic classification in QIIME2 employed the classify-sklearn plugin with the default confidence threshold of 0.7 for the --p-confidence parameter, as implemented in Novogene’s standardized ITS pipeline. This threshold corresponds to a posterior probability cut-off applied sequentially from kingdom to species such that each ASV is assigned to the deepest taxonomic rank with a cumulative classification probability of at least 0.7, thereby reducing low-confidence over-classification. A confidence value of 0.7 indicates that the classifier is 70% confident that the sequence belongs to the assigned taxon. ASV read counts were converted to relative abundances and exported as Excel spreadsheets, which were subsequently imported into the custom “Fungi Metagenomic Analysis” R Shiny application (https://jerricasng.shinyapps.io/shiny_app/) for downstream analyses using RStudio (v2026.01.0+392). The application integrates multiple R packages: shiny, tidyverse (including dplyr, readr, readxl, tibble, purrr, stringr, and tidyr), vegan, ggplot2, pheatmap, reshape2, RColorBrewer, openxlsx, xml2, and rentrez.

It accepts (i) a sample metadata file (.csv/.xlsx) and (ii) multiple sequencing batches consisting of ASV relative-abundance tables together with their corresponding ASV sequence files (.FASTA/.txt). During import, UNITE taxonomic strings were parsed into standard ranks (kingdom to species) and species-hypothesis identifiers (SH IDs) where available. Missing ranks, SH IDs, and placeholder annotations (e.g., “sp.”, “gen.”, “incertae sedis”) were standardized to “Unknown “or “Not given”. To ensure unique identifiers across sequencing batches, batch-tagged ASV IDs were generated and used to merge all batches into combined abundance, sequence, and taxonomic tables for downstream analyses. The application also includes a missing-BLAST checker to verify that missing ASV matches are due to the absence of corresponding entries in the database rather than temporary website or query errors.

To improve taxonomic classification, the application identified ASVs lacking assignment at a user-selected taxonomic rank (kingdom to species) or species-hypothesis identifier (SH ID) and exported their corresponding sequences as FASTA files. These ASVs were queried using NCBI BLAST (megablast) against the ITS RefSeq Targeted Loci database. BLAST hit tables (.csv/.xlsx) were subsequently reimported and filtered using user-defined minimum percentage identity and maximum E-value thresholds, after which taxonomic lineages for subject accessions were retrieved programmatically from NCBI. In this study, an E-value threshold of ≤1 × 10^−25^ and a percentage identity threshold of ≥ 90% were used for conclusive identification. For each ASV, only the single best hit from the filtered results was retained. BLAST-derived taxonomy was then integrated with the original UNITE-based assignment provided by Novogene and updated only when BLAST provided a deeper classification than UNITE. The output table recorded the source database (UNITE or BLAST) and whether an ASV was included in the BLAST analysis.

Exploratory community profiling was performed with taxonomic composition plots (pie charts and stacked bar plots); alpha-diversity analyses (observed ASVs, Shannon indices, and Simpson indices); beta-diversity analyses based on Bray–Curtis dissimilarities (distance heatmaps, hierarchical clustering dendrograms, non-metric multidimensional scaling [NMDS] ordinations, within-group beta-diversity boxplots, and PERMANOVA); and Euclidean distance-based ordinations (PCA and PERMANOVA). All analyses and plots were performed using metadata-defined groupings such as sampling location and substrate type. Detailed descriptions of the statistical analyses and visualization methods are provided in the Supplementary Material (Section S2).

Fungal nomenclature and author citations were verified against the Index Fungorum online database (https://www.indexfungorum.org/names/names.asp; accessed on 24 September 2026).

### 2.4. Fungal Culture and Molecular Identification

Potato Dextrose Agar (PDA) (39 g/L; Millipore, Merck KGaA, Darmstadt, Germany) was supplemented with filter-sterilized ampicillin (100 µg/mL; Gold Biotechnology^®^, St. Louis, MO, USA) and streptomycin (30 µg/mL; PanReac AppliChem, ITW Reagents, Darmstadt, Germany) to inhibit both Gram-positive and Gram-negative bacteria [30,31], as bacterial contamination may affect fungal growth [32]. Wood-sample suspensions were prepared by fragmenting decayed wood samples in 1 mL of sterile Milli-Q water. Fungal cultures were then obtained by spread-plating 100 µL aliquots of undiluted suspension and a 1:10 dilution onto PDA plates. Plates were sealed with air-permeable surgical tape (Micropore™ tape, 3M) and incubated at 28 °C in darkness. Pure cultures were subsequently obtained through repeated subculturing using the touch-and-go method.

DNA was extracted from pure fungal cultures using the modified Lyticase–Chelex-100 protocol described in Section 2.2. PCR amplification for fungal isolates and fruiting bodies was performed in 50 µL reaction mixtures using Q5 High-Fidelity DNA Polymerase (New England Biolabs, Ipswich, MA, USA) according to the manufacturer’s instructions, with 4 µL of template DNA per reaction. Cycling conditions consisted of an initial denaturation at 98 °C for 30 s, followed by 35 cycles of 98 °C for 10 s, 52 °C (ITS1) or 55 °C (ITS) for 30 s, and 72 °C for 30 s, with a final extension at 72 °C for 2 min.

The primer pair ITS5 (5′-GGAAGTAAAAGTCGTAACAAGG-3′) and ITS2 (5′-GCTGCGTTCTTCATCGATGC-3′) was used to amplify the ITS1 region, while the primer pair V9D (5′-TTAAGTCCCTGCCCTTTGTA-3′) and LS266 (5′-GCATTCCCAAACAACTCGACTC-3′) was used to amplify the complete ITS region.

PCR amplicons were purified using the EZ-10 Spin Column PCR Products Purification Kit (Bio Basic Asia Pacific Pte. Ltd., Singapore) with the minor modifications described above. DNA concentration and purity were assessed using a NanoDrop 1000 spectrophotometer (Thermo Scientific, Waltham, MA, USA), and PCR products were verified by agarose gel electrophoresis. Successful PCR amplicons were submitted to Bio Basic Asia Pacific for Sanger sequencing using the primer pairs ITS2/ITS5 (ITS1 region) or ITS1/ITS4 (complete ITS region). Raw chromatograms were processed using Unipro UGENE, reverse reads were reverse-complemented, and forward and reverse reads were aligned using MUSCLE (Multiple Sequence Comparison by Log-Expectation). The final consensus sequences were then queried against the NCBI BLAST database “Internal transcribed spacer region (ITS) from Fungi type and reference material” dataset and limited only to sequences from type material for taxonomic identification.

### 2.5. In Vitro Wood-Decay Assays and Analysis

In vitro wood-decay assays were conducted using isolates for three fungal species—*Paecilomyces maximus* C. Ram, *Schizophyllum commune* Fr., and *Phanerochaete* P. Karst. sp.—whose identities were confirmed by Sanger sequencing and NCBI BLAST analysis. For each species, 36 PDA plates supplemented with antibiotics were inoculated with mycelial plugs and incubated at 28 °C until the agar surface was fully colonized by fungal mycelia.

Wood blocks (30 × 10 × 8 mm) prepared from four tree species—angsana (*Pterocarpus indicus*) (A), rubberwood (*Hevea brasiliensis*) (R), spruce (*Picea* sp.) (S), and balau (*Shorea* sp.) (B)—were oven-dried at 100 °C for 4 days to constant weight and sterilized by double autoclaving at 121 °C for 20 min. For each fungal treatment, wood blocks were placed onto actively growing fungal cultures or uninoculated negative-control plates, with nine replicate plates containing two wood blocks each (18 blocks per treatment). Plates were sealed with air-permeable surgical tape and incubated at 28 °C.

Blocks inoculated with *P. maximus* and *S. commune* (27 March 2025) were collected at 2 weeks (10 April 2025), 6 weeks (8 May 2025), and 4 months (28 July 2025) post-inoculation, with six blocks collected for each fungus–wood combination at each time point. Blocks inoculated with *Phanerochaete* sp. (3 April 2025) were harvested after 5 weeks (8 May 2025) and 4 months (28 July 2025), again with six blocks per combination at each time point. Four negative control blocks per tree species were collected at every sampling point. Surface mycelia were gently removed using C-fold paper towels, after which the wood blocks were oven-dried at 100 °C for 3 days before final dry weights were recorded. Percentage mass retained was subsequently calculated using Microsoft Excel.

One-tailed Welch’s *t*-tests assuming unequal variances were performed in Microsoft Excel to determine whether fungal treatments resulted in a significantly lower percentage of mass retained relative to the negative controls. Results were visualized as bar plots showing the mean percentage mass retained, with standard deviation error bars and significance levels indicating the differences between comparing the control group (wood block only) and the inoculated group (wood block with fungal mycelia).

## 3. Results

### 3.1. Ascomycota and Basidiomycota Dominated All Sites

Across the 133 samples, *Ascomycota* and *Basidiomycota* dominated the decayed wood-associated mycobiome, accounting for 45.4% and 41.2% of the total relative abundance. The remaining 10 fungal phyla collectively contributed only 0.43%, while the remaining reads could not be conclusively classified taxonomically.

Phylum-level community composition varied among sampling sites. Some sites were strongly dominated by *Ascomycota* and others by *Basidiomycota*, whereas only two sites exhibited relatively similar contributions from both phyla (Table S1).

### 3.2. Alpha Diversity Within and Across Locations

Alpha diversity was assessed using the Simpson index to evaluate genus-level diversity and dominance patterns within the fungal communities.

#### Simpson Index

**Figure 4:**
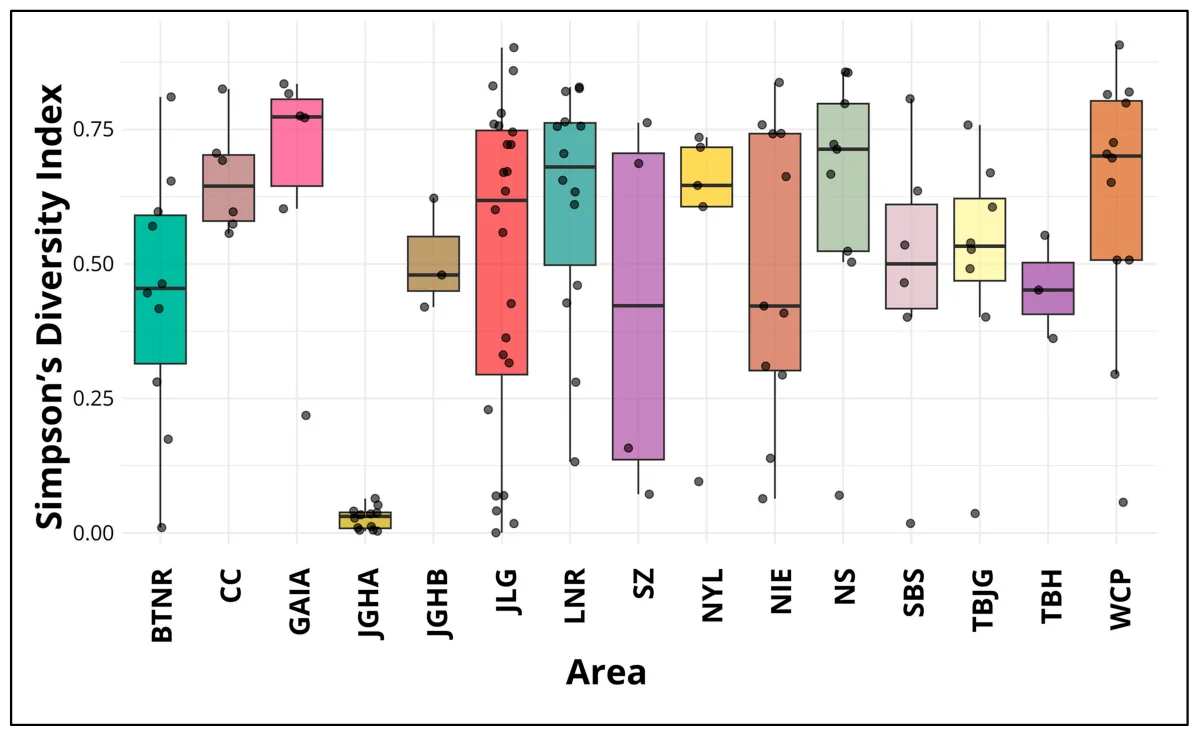
Boxplots of Simpson’s diversity index for fungal genus-level communities across sampling sites. Each point represents individual wood sample.

Simpson indices were calculated for all 133 samples and grouped by sampling location (Figure 4). The results revealed substantial variation in alpha diversity both among and within locations. All but one site, including GAIA, Night Safari, West Coast Park, and Labrador Nature Reserve, exhibited high mean Simpson values, indicating greater fungal genus diversity and lower dominance by any single genus. Simpson values within individual locations varied widely, ranging from approximately 0 to 0.80, with site-level means between 0.4 and 0.7. This broad variation indicates that the degrees of genus dominance differed considerably among samples collected from the same location. In contrast, samples from Jalan Greja House-A (JGHA) exhibited Simpson values close to 0 across all samples, indicating strong dominance by one or few genera at that location.

### 3.3. Fungal Community Composition Across Sites

Non-metric multidimensional scaling (NMDS) based on Bray–Curtis dissimilarities was used to visualize differences in fungal community composition across sites (Figure S33). The ordination revealed partial clustering of samples according to location, with only the JGHA samples forming a distinct cluster, whereas samples from the remaining locations showed substantial overlap in their 95% confidence ellipses. The NMDS stress value of 0.273 indicates that the two-dimensional ordination provides only a moderate representation of the underlying dissimilarities and should therefore be interpreted as a general visualization rather than a precise representation of the multivariate community structure. PERMANOVA analysis based on Bray–Curtis dissimilarities revealed a significant effect of location on fungal community composition (F = 2.82, R^2^ = 0.25, *p* = 0.001), indicating that sampling location explained approximately 25.1% of the observed variation in community structure. Together with the NMDS ordination, these results suggest that although geographic location significantly influences fungal community composition, a substantial proportion of the variation is likely driven by additional environmental or biological factors.

**Figure 5:**
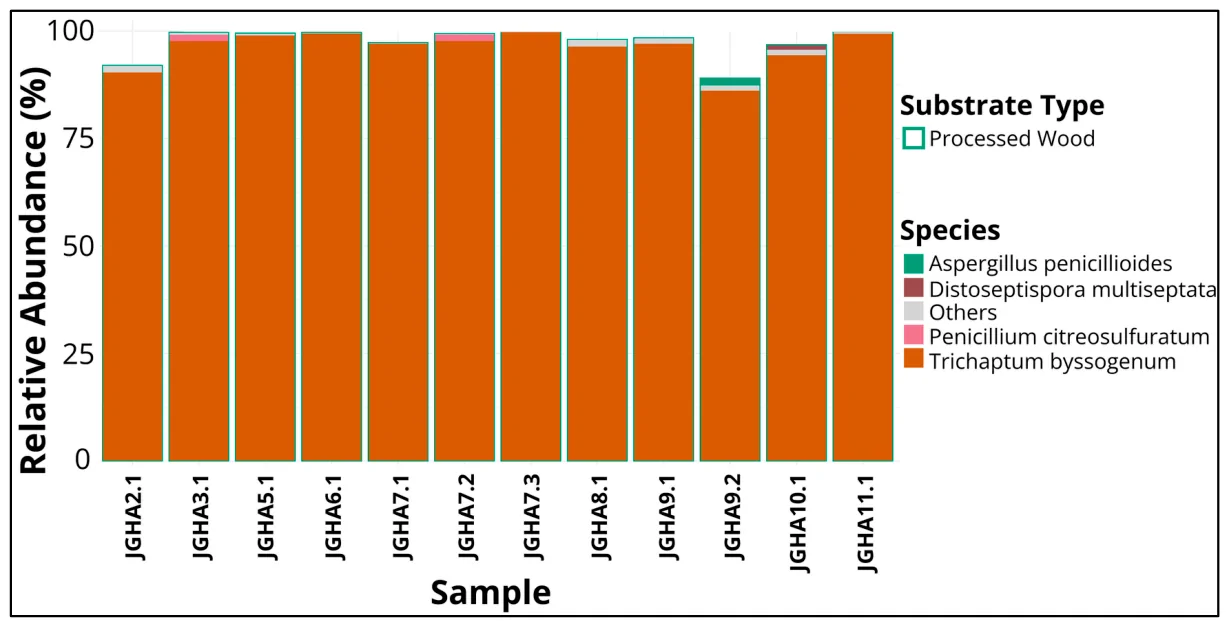
Relative abundance of fungal species detected in samples collected from JGHA. Consistent dominance of *Trichaptum byssogenum* was observed across all samples.

However, the NMDS ordination (Figure S33) also revealed that samples from JGHA clustered tightly and exhibited a relatively small 95% confidence ellipse, indicating low within-site compositional variation. This pattern is consistent with the Simpson diversity index boxplot (Figure 4), which showed low variability among JGHA samples, characterized by a narrow interquartile range and minimal spread. This observation is further supported by the species-level data (Figure 5), in which nearly all samples were dominated by *Trichaptum byssogenum* (Jungh.) Ryvarden. Because this location was unusually homogeneous and taxonomically skewed (Bray–Curtis dissimilarity ≤ 0.09 for all pairwise comparisons within JGHA) relative to other locations (Section S3.3; Figures S2–S16), it may have disproportionately influenced the PERMANOVA results, inflating the proportion of variance explained by location. Therefore, PERMANOVA was repeated after excluding JGHA samples (F = 1.76, R^2^ = 0.18, *p* = 0.001). Although the effect of location remained significant, the lower F value indicated weaker separation among locations, while the lower R^2^ value (18%) suggested that fungal community variation was less strongly associated with location after removal of the outlier site.

A Bray–Curtis beta-diversity heatmap across all sampling locations (Section S3.2; Figure S1) revealed high pairwise dissimilarities among sites ranging from 0.61 to 1.0, with mean and median values of 0.92 and 0.95 respectively. These results support the PERMANOVA and NMDS analyses, together indicating that fungal community composition differed substantially among sites.

With most sites exhibiting very high pairwise dissimilarity (Bray–Curtis dissimilarity), WCP and BTNR showed comparatively more similarity (Bray–Curtis dissimilarity = 0.61) despite being separated by 7.34 km. This observation suggests that factors other than geographic distance may also contribute to shaping fungal community composition.

To further investigate compositional similarity between WCP and BTNR, a Bray–Curtis beta-diversity heatmap was generated for samples from these two locations (Section S3.4; Figure S17). The heatmap revealed notable similarities among several sample pairs. Samples BTNR2.1 and WCP2.1 (both processed wood) exhibited a Bray–Curtis dissimilarity of 0.39, while BTNR2.1 and WCP9.1 (also both processed wood) showed a dissimilarity of 0.40. The most similar pair was BTNR9.1 (natural log) and WCP8.1 (processed wood), which exhibited a Bray–Curtis dissimilarity of only 0.11, indicating highly similar fungal genus-level communities despite differences in substrate type and geographic location.

**Figure 6:**
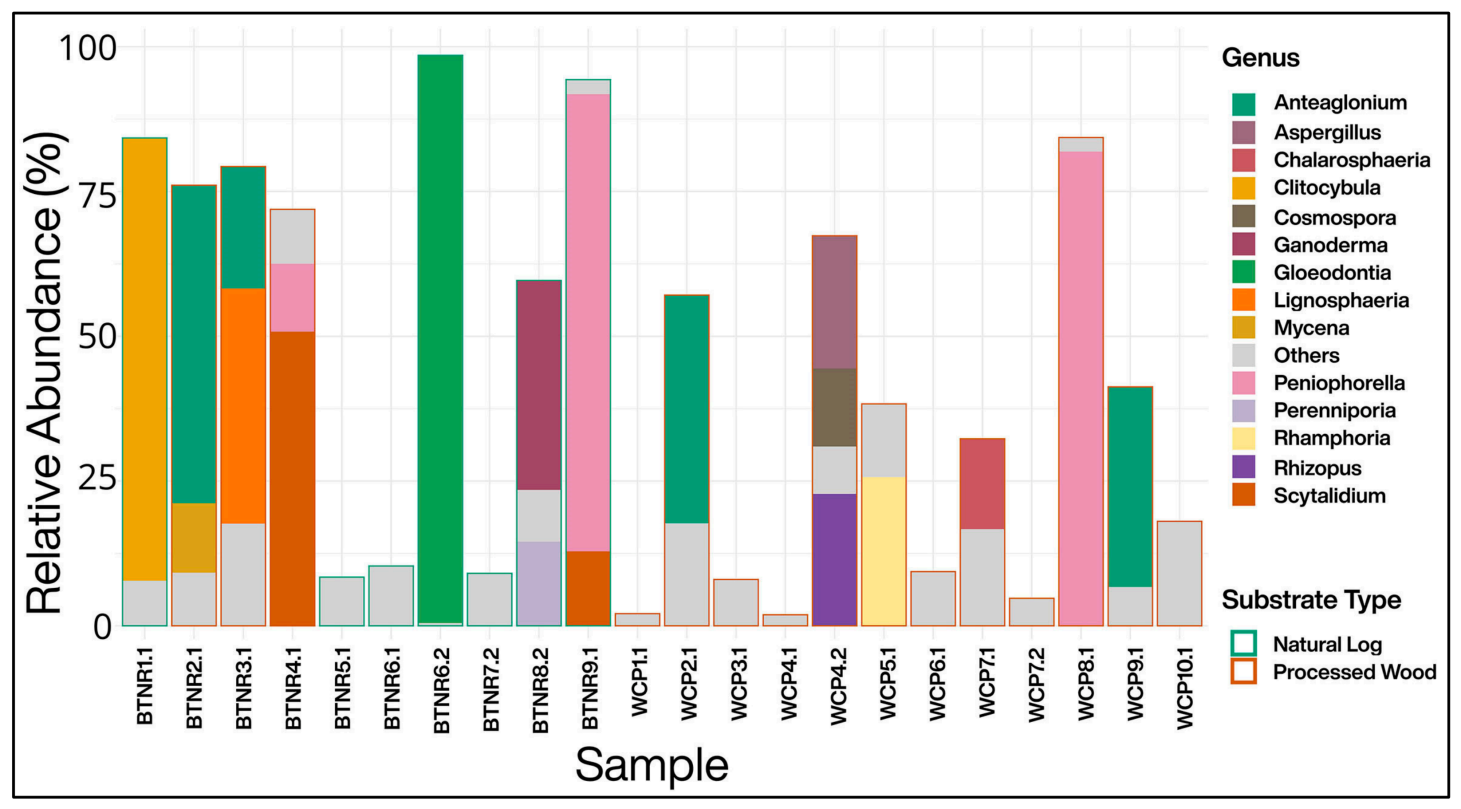
Relative abundances of fungal genera detected in samples collected from WCP and BTNR, excluding taxonomically inconclusive genera. “Others” represents taxa with relative abundances of ≤10%.

Genus-level bar plots for BTNR and WCP (Figure 6) revealed overlapping dominant genera across multiple samples, consistent with the genus-level beta-diversity patterns observed in Figures S1 and S17. Examination of genus-level composition further confirmed that highly similar sample pairs, including BTNR2.1 with WCP2.1 and WCP9.1, as well as BTNR9.1 with WCP8.1, shared several dominant genera, as summarized in Table 2.

**Figure 7:**
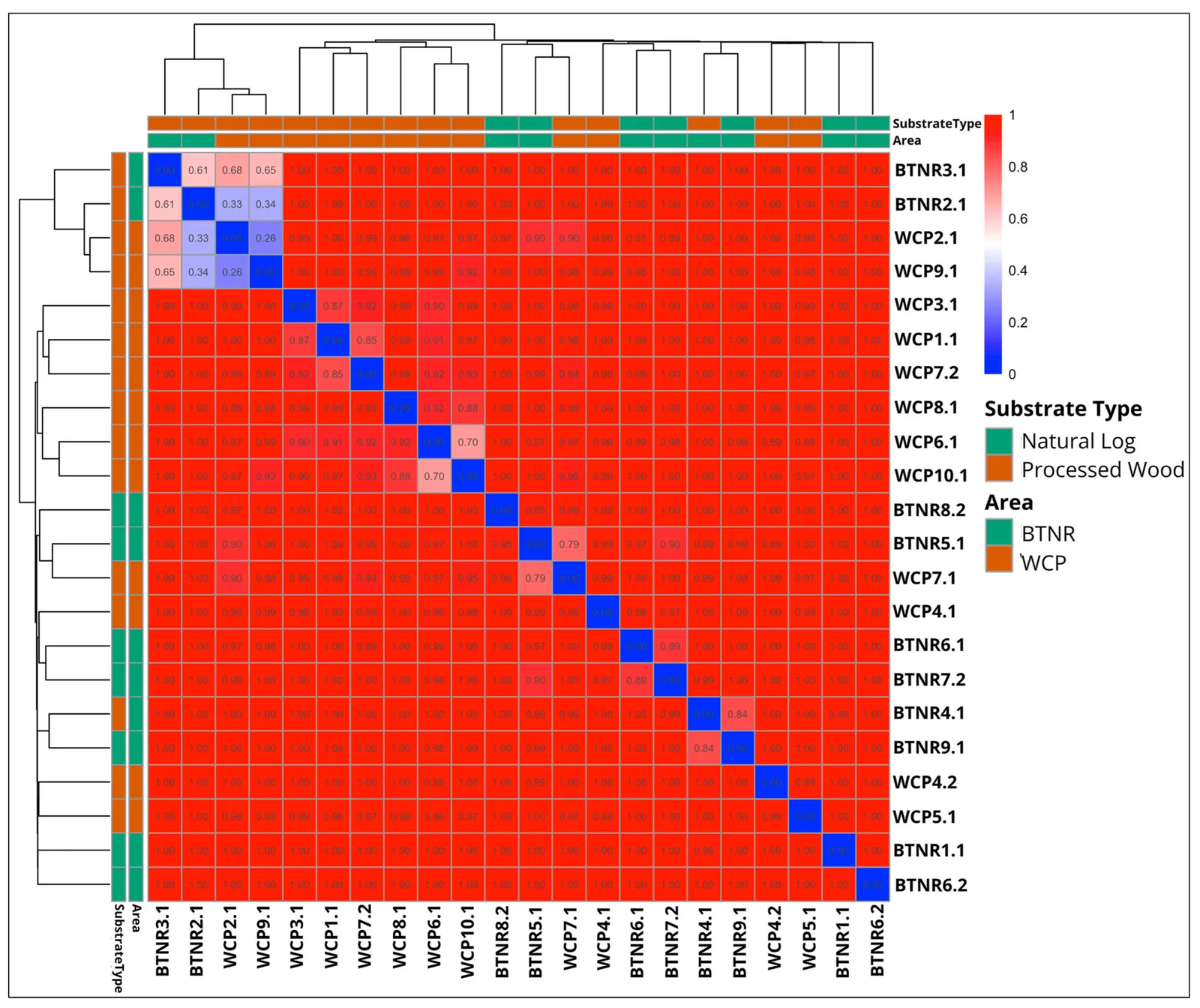
Bray–Curtis beta-diversity heatmap showing species-level dissimilarities among fungal communities in samples collected from WCP and BTNR, excluding taxonomically inconclusive species.

When beta diversity was recalculated at the species level (Figure 7), the sample pairs of BTNR2.1–WCP2.1 and BTNR2.1–WCP9.1 continued to exhibit relatively low dissimilarities of 0.33 and 0.34, respectively, whereas the sample pair BTNR9.1–WCP8.1 shifted to complete dissimilarity (Bray–Curtis dissimilarity = 1.00). These results indicate that the apparent genus-level convergence between BTNR9.1 and WCP8.1 was driven by different species belonging to shared genera (Table 2).

### 3.4. Fungal Community Composition Between Substrate Types

**Figure 8:**
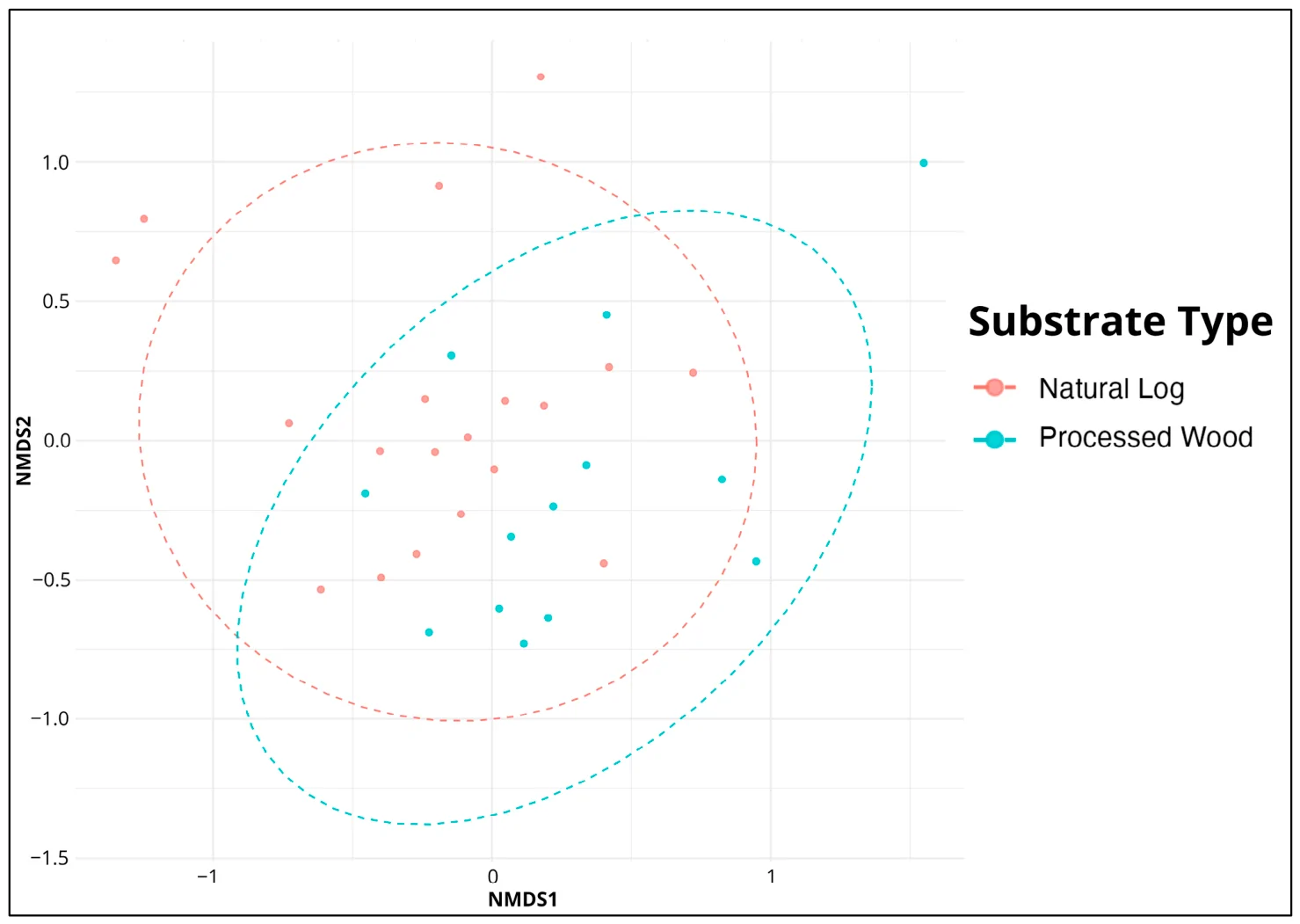
NMDS ordination of fungal communities at BTNR, LNR and JGHB, showing differences between natural log and processed wood samples (stress = 0.217). Ellipses represent 95% confidence intervals for each substrate type.

As sampling location did not fully explain the observed variation in fungal community composition, NMDS and PERMANOVA analyses were also used to evaluate differences between the two substrate types: natural logs and processed wood. The NMDS ordination (Figure 8) revealed substantial overlap between fungal communities associated with natural logs and processed wood, with no clear separation between the two substrate categories. PERMANOVA analysis (F = 1.46, R^2^ = 0.046, *p* = 0.006) indicated that although substrate type had a statistically significant effect on fungal community composition, the magnitude of this effect was small, with substrate type explaining only 4.6% of the observed variance. Together, these findings suggest that fungal communities associated with natural logs and processed wood are largely similar, potentially reflecting community convergence over time, and that substrate type alone is unlikely to be a major determinant of fungal community composition relative to other environmental or ecological factors.

Both comparisons reveal that other factors like the environment or wood type may play a greater role in shaping fungal compositions as compared to geographical location and substrate type.

### 3.5. Within-Site Fungal Community Analysis

As the NMDS ordinations and PERMANOVA analyses revealed only modest differences among sampling locations, further analyses were conducted to investigate variation in fungal community composition within individual locations. Bray–Curtis beta-diversity heatmaps were generated for each site (Figures S2–S16). Overall, most sampling sites exhibited high pairwise dissimilarities among samples, with median within-site Bray–Curtis dissimilarities ranging from 0.60 to 0.98. These results indicate that even within the same location, individual wood pieces generally harbor distinct fungal communities, highlighting local-scale heterogeneity as a prominent feature of decayed wood mycobiomes. An exception was observed for samples from JGHA, which were largely dominated by *Trichaptum byssogenum* (Figure 5).

### 3.6. Same Wood Piece Fungal Community Analysis

To investigate microenvironmental variation within individual wood pieces, fungal community composition was compared between samples collected from different positions on the same piece of wood structure or log, which were hypothesized to harbor highly similar fungal communities. With the exception of sample pairs collected from JGHA, which was dominated by a single fungal species, most pairs from other locations exhibited high dissimilarity values (≥0.50), with only two pairs (LNR11.1-LNR11.2 and CC3.1–CC3.2) showing moderate similarity (0.24 and 0.32, respectively). These findings indicate that even within a single wood structure or log, neighboring microenvironments may support substantially different fungal communities. For example, the four samples from one decaying log (JLG1.1, 1.2, 1.3, and 1.4) harbored distinct fungal communities with pairwise dissimilarity values between 0.71 and 1.

The pair NIE1.1/NIE2.1 were from two different wood structure pieces but were accidentally mixed during DNA extraction (Section 2.2). We used them as technical duplicates for testing the robustness and reliability of the amplicon sequencing workflow, which proved to be excellent with a very low dissimilarity (0.03) between them (Table 3).

### 3.7. Phylum Composition of Dominant Fungal Genera

**Figure 9:**
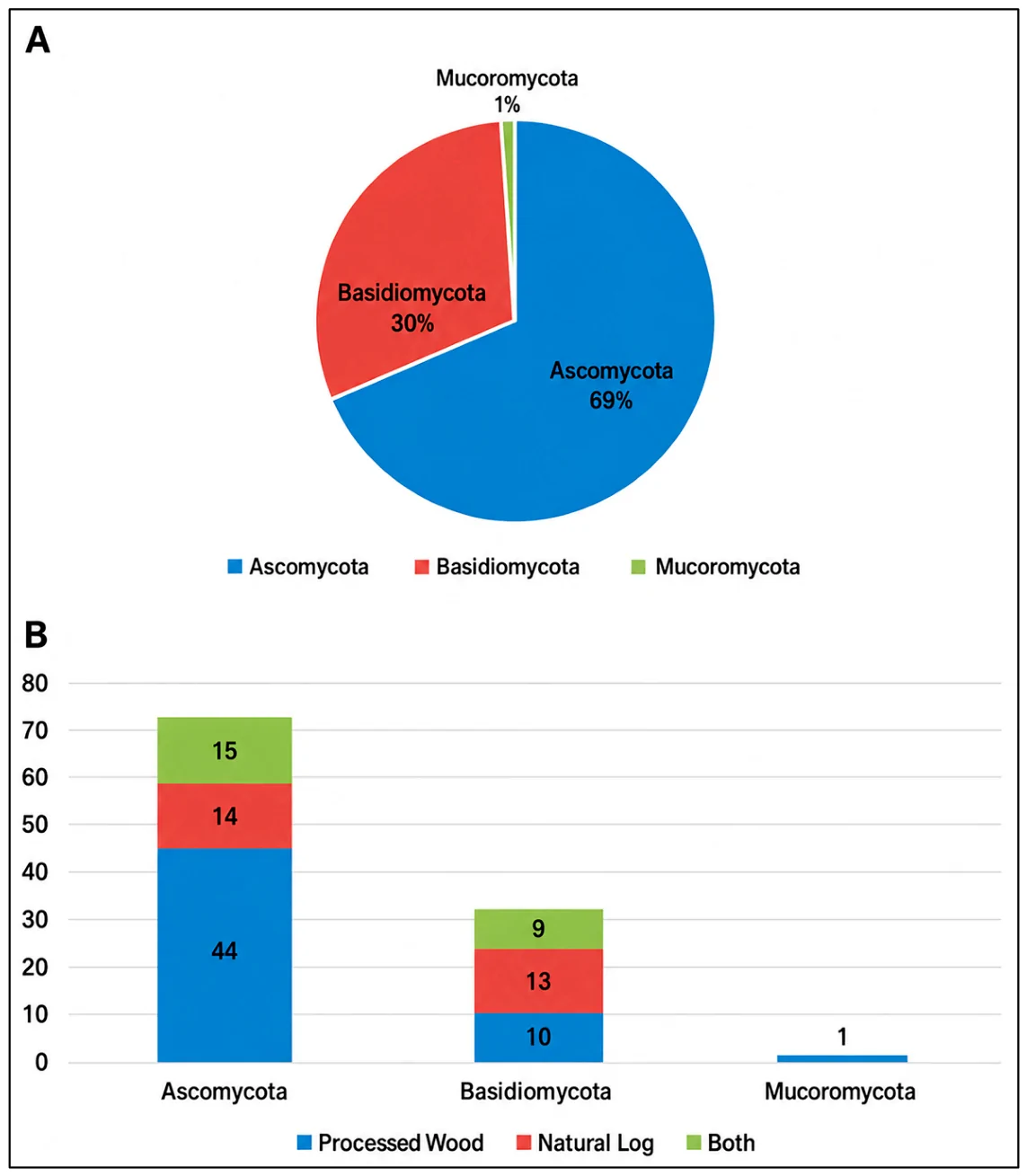
Phylum-level composition and substrate association (processed wood, natural logs, or both) of dominant fungal genera. (**A**) Phylum-level distribution of dominant genera (≥10% relative abundance). (**B**) Number of dominant genera within each phylum according to substrate type.

A dominant genus is defined as a genus with 10% or more relative abundance in a sample. Overall, there are 106 dominant genera identified by our study. The phylum composition of these genera is summarized in Figure 9A.

Although the overall abundance of *Ascomycota* across all samples was only slightly higher than that of *Basidiomycota* (45.4% vs. 41.2%), *Ascomycota* accounted for a substantially larger proportion of the dominant genera (69%) compared with *Basidiomycota* (30%; Figure 9A), suggesting that ascomycetes may establish dominance more readily than basidiomycetes. It is notable that more than half of the dominant genera assigned to *Ascomycota* (44 out of the 73) were specifically associated with processed wood decay, 14 were specific to natural wood decay and 15 were present in both substrate types. For dominant genera assigned to *Basidiomycota*, similar numbers of genera were specific to processed wood, specific to natural log and present in both substrates, with 10, 13 and 9 respectively (Figure 9B). Table 4 details the dominant fungal genera in our samples, number of locations with their presence, and published experimental evidence for their involvement in wood decay. Additional information on sampling locations and substrate types is provided in Table S4.

Among the dominant genera detected, most fungi within *Basidiomycota* (22 out of 32) have previously been reported as wood decaying fungi. Two exceptions are the *Tulasnella* sp., which was reported to lack the relevant enzymatic activities and their associated genes, and the *Coprinopsis* sp. which showed no wood-decay ability (Table 4). For five taxa, adjoining fruiting bodies were also collected and independently identified as belonging to the same taxon (Table 4). For the basidiomycetes with proven wood-decay activities, their detection in decayed wood in Singapore extends their documented occurrence to tropical environments and supports a similar ecological role in wood decomposition. Taxa associated with both log and processed wood or specifically with processed wood may be of particular relevance to the wood industry.

Among the dominant ascomycetes, only 12 of the 63 genera have experimental evidence demonstrating measurable wood mass loss. For several others, cultured isolates have shown activities of lignocellulose-degrading enzymes, suggesting potential roles in wood degradation, although such evidence does not by itself demonstrate decay of intact wood. Conversely, one genus has been reported to lack the relevant enzymatic activities examined (Table 4). The majority of the dominant *Ascomycota* fungi had their presence in decay wood reported but their functions in wood decay not experimentally investigated.

Notably, only nine genera were dominant across multiple sampling sites (four–seven sites, representing 27–47% of all sites). These widely distributed dominant genera comprised eight ascomycete genera (*Chalarosphaeria* W.P. Wu & Y.Z. Diao, *Cladophialophora* Borelli, *Exophiala* J.W. Carmich, *Pseudodactylaria* Crous, *Rhexodenticula* W.A. Baker & Morgan-Jones, *Sporidesmium* Link, *Teratosphaericola* Quaedvl. & Crous, and *Trichoderma* Pers.), and only one basidiomycete genus, *Peniophorella* P. Karst. (Table S5).

*Peniophorella*, a white-rot basidiomycete [83], is the most cross-site-dominant basidiomycete genera identified by our study, detected in seven sites. It is known for wood colonization and is present across all forested continents [84]. *Hyphoderma praetermissum* (now *Peniophorella praetermissa* (P. Karst.) K.H. Larss.) was reported to cause 36–60% weight loss for sweetgum and 28–51% weight loss for white fir. Another report by Schilling et al. tested *Peniophorella praetermissa* in an aspen wood-decay assay and found a negligible mass loss of 0.06% (± 0.11 SE) at two weeks and 0.10% (± 0.08 SE) at four weeks [83], and suggested that it was not an early wood colonizer. Its widespread dominance in our samples raises the possibility that it may function as a mid- to late-stage primary wood decomposer in Singapore.

Table S5 summarizes the locations where the eight widely distributed dominant ascomycete genera were detected, together with their reported ecological functions and potential wood-decay roles [85,86,87,88,89,90,91,92,93,94,95,96,97]. Most of these genera remain poorly characterized as wood-decay agents. Although many have been reported as saprotrophs associated with dead plant material, including submerged decaying wood, experimental characterization of their specific decay mechanisms or rot types remains limited (Table S5).

### 3.8. In Vitro Wood-Decay Assays

**Figure 10:**
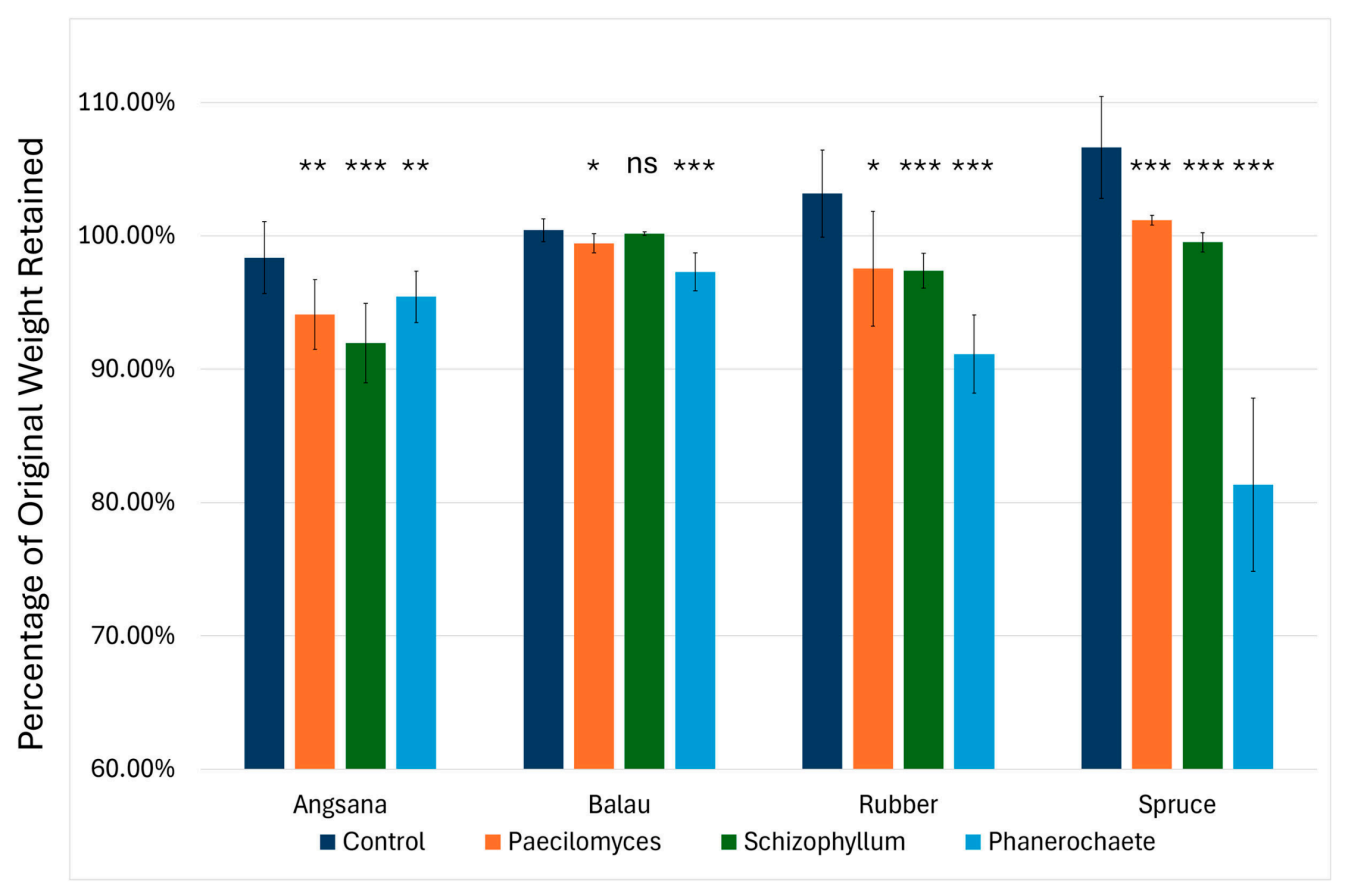
Percentage of original wood mass retained after in vitro fungal treatment with three fungal species across four wood types. Statistical significance is indicated by ns (not significant), significant with *p* < 0.05 (*), very significant with *p* < 0.01 (**), and highly significant with *p* < 0.001 (***).

In vitro wood-decay assays were conducted using pure isolates of *Paecilomyces maximus*, *Schizophyllum commune*, and *Phanerochaete* sp. Statistical differences in wood mass loss among fungal treatments were evaluated using Welch’s one-tailed *t*-test. Only wood blocks collected at the final sampling time point (28 July 2025) were included in the mass-loss analysis, corresponding to 4 months post-inoculation for *Paecilomyces maximus* and *Schizophyllum commune*, and slightly less than 4 months for *Phanerochaete* sp. The results are summarized in Figure 10. Four wood species commonly used in urban landscaping and structural wood applications in Singapore—angsana, balau, rubber, and spruce—were included in the assay.

For *Paecilomyces maximus*, an ascomycete, the assay demonstrated its ability to independently degrade wood. Significant mass loss was observed in balau and rubberwood blocks (*p* < 0.05), very significant mass loss (*p* < 0.01) in angsana wood, and highly significant mass loss (*p* < 0.001) in spruce wood.

For *Schizophyllum commune*, a basidiomycete, highly significant mass loss (*p* < 0.001) was observed in angsana, rubberwood, and spruce blocks. However, no significant mass loss was detected in balau wood, which is notable given that *Schizophyllum commune* is a well-known primary wood-decay fungus.

For *Phanerochaete* sp., a primary wood-decay basidiomycete, significant mass loss was observed across all four wood types, with angsana wood showing significant mass loss (*p* < 0.01) and the remaining three wood types showing highly significant mass loss (*p* < 0.001).

## 4. Discussion

Across the 15 sampling sites in Singapore, no fungal genus consistently dominated decayed wood structures or natural logs, which is defined as presence in more than half of sample sites, and the fungal community composition differed significantly both across and within sampling sites. Moreover, sampling location and substrate type explained only a relatively small proportion of the variation in fungal community composition (23.8% and 5.9%, respectively), indicating that these broad-scale factors were significant but insufficient determinants of fungal community structure. Notably, paired samples collected only centimeters apart on the same wood piece generally exhibited high dissimilarity, revealing substantial fine-scale heterogeneity in fungal communities. This pattern is consistent with the hypothesis proposed by Bosch et al. [98] that mycobiome composition in decayed wood is largely stochastic. They studied fungal communities in fine-scale 1 cm^3^ cylinders (comparable to our 1 g sampling size) collected from 10 decaying European beech wood logs (three cylinders per log) and found no significant difference in fungal alpha diversity between fine-scale samples (1 cm^3^ cylinder) and composite samples (pooled samples of the three cylinders per log). Moreover, the three fine-scale samples from the same log were not closely clustered together in the NMDS plot and were distinct from their composite sample. Together, our findings suggest that microscale environmental factors may exert a strong influence on fungal community assembly. Consistent with this suggestion, a study of the fine-scale distribution of arbuscular mycorrhizal (AM) fungi in semiarid Mediterranean scrub lands found plant community composition, soil physicochemical properties and spatial distance to be the three primary drivers for AM fungus composition [99]. Another recent in vitro study of hyphae growth and turnover of the six wood-decay fungi identified fungal species and nutrition as the two key determinants for hyphal growth and turnover [100]. We hypothesize that microscale ecological differences likely play important roles in determining the wood-decay fungus community in the tropics. This hypothesis somehow deviates from the hypothesis that filamentous fungi grow through wood and establish spatially distinct territories separated by interaction zones, where competing species suppress one another, thereby enabling colonization by one or a few dominant fungi [101,102,103,104].

The dominance of a single fungal species, *Trichaptum byssogenum*, at one sampling site was exceptional to this study. All samples from this site were collected from decayed wood planks, presumed to be Shorea sp., that had been dismantled from a house porch. Visible white mycelial growth was present on the underside of all planks and was molecularly identified as *T. byssogenum*. One possible explanation was that the persistently dark and moist conditions beneath the wooden porch favored extensive mycelial growth, allowing *T.* byssogenum to colonize much of the structure. This interpretation is consistent with the observation that samples from other locations were more exposed to sunlight and external environmental conditions. Alternatively, the wood may have been infected before processing, followed by undergoing rapid fungal proliferation after installation under favorable conditions. This possibility is supported by the absence of the same fungal taxon in samples collected from the neighborhood. Further study is needed to conclude either of the possibilities.

Several limitations should be considered when interpreting our findings. Firstly, ITS1 metabarcoding detects DNA rather than metabolic activity; the occurrence or relative abundance of a taxon in decayed wood cannot, on its own, establish that it was actively degrading the sampled material. Fruiting-body observations, culture recovery, and wood-block decay assays provide additional evidence for selected taxa, but do not resolve the activity or contribution of every detected taxon in situ. Secondly, our study focused on two macro-factors (location and substrate type), but other variables might have contributed to the fungal community diversity that we identified. Wood species may also contribute to the heterogeneity of fungal communities, but we were unable to evaluate this factor because most natural logs collected in this study were too heavily decomposed for reliable species identification, and the identities of many wood structures were unknown, even to park rangers. To avoid misclassification and associated analytical bias, wood species identity was therefore excluded from the analyses. Information on timber origin, preservative treatment, installation date, and maintenance or replacement history was also unavailable for many processed wood structures. Therefore, wood structure age was not assessed as a predictor of fungal community composition. While our finding of distinctive mycobiomes in paired samples from the same wood structure or log upholds our conclusion of fine-scale fungal community diversification, variations in wood species and associated nutrient availability have likely contributed to the fine-scale heterogeneity in fungal community observed in our study. Another limitation is that our study provides only a snapshot of the intermediate to late stages of wood decay and may not be representative of fungal communities at other stages of the decay process, as studies have shown that decay stage and wood substrate quality significantly affect fungal species richness and the nature of diversity–decomposition relationships [105].

A significant finding from our study is the high proportion of fungi within the *Ascomycota* phylum among the dominant genera detected in the samples (69% vs 30% of fungi within *Basidiomycota* phylum; Figure 9). Such ascomycete dominance is further supported by the finding that eight of the nine widely present dominant genera (Table 4) belong to *Ascomycota*, indicating that fungi from the *Ascomycota* phylum were widely represented among the sampled decayed woods. However, sequence occurrence and relative abundance do not quantify degradation activity, so the respective contributions of these fungi to wood decay remain to be resolved. This pattern is consistent with several studies conducted in tropical and subtropical environments, where *Ascomycota* was similarly reported as the dominant phylum in decaying wood [14,106,107]. One possible explanation is that, during the later stages of wood decomposition, wood substrates become more humid and nutrient-rich, conditions that favor the activity of soft-rot ascomycetes [108]. This interpretation is consistent with our sampling strategy of only collecting from wood showing visible signs of decay, hence representing mid- to late-stage decomposition. Fungi within *Ascomycota* are also known to exhibit strong competitive interactions against basidiomycetes, which may further contribute to their dominance in such environments [107,108]. Their competitive advantage may be enhanced by their ability to adapt to changing environmental conditions through the production of extracellular enzymes and secondary metabolites, including antibiotic, fungitoxic, and fungistatic compounds [109,110,111,112]. Twelve of the dominant ascomycete genera identified in our study (Table 4) belong to lineages containing experimentally demonstrated wood decayers, including one of the widely distributed dominant ascomycete genera, *Trichoderma,* identified in four sites. For several additional genera, cultured isolates have shown lignocellulose-degrading enzyme activities, suggesting potential roles in wood degradation (Table 4). The other seven widely dominant ascomycete genera are known primarily as saprotrophs associated with dead plant material or submerged wood (Table S5). These ecological records make their presence in our samples plausible but do not establish that they caused the observed decay. Some experimental studies have shown that selected tropical freshwater fungi can cause wood mass loss and modify lignin, illustrating potential routes by which such fungi may contribute to decomposition [92,113]. They may be readily dispersed by rainfall and may contribute to the biodeterioration of outdoor wood structures in Singapore and other tropical regions with frequent and heavy rainfalls. Functionally, *Paecilomyces maximus*, an ascomycete, demonstrated its in vitro ability to independently degrade wood in the absence of any basidiomycete. Significant mass loss was observed in balau and rubberwood blocks (*p* < 0.05), very significant mass loss (*p* < 0.01) in angsana wood, and highly significant mass loss (*p* < 0.001) in spruce wood. Balau, rubberwood and angsana are common tree species for outdoor wood structures, and spruce wood serves as the main source of MET commonly used in Singapore. This preliminary finding supports the possibility that ascomycetes may serve as primary wood degraders, consistent with the report that some higher ascomycete fungi caused substantial mass loss in birch (*Betula* sp.) wood in the absence of basidiomycetes and were capable of independently degrading syringylpropane lignin units [22]. However, it remains to be proven if all the dominant ascomycetes we identified could degrade wood independently, and their mechanisms of wood decay, especially how they degrade lignin, are also unknown.

Our main interest was the decay of outdoor wood structures (processed wood decay) and we initially hypothesized that some basidiomycete fungi would dominate tropical wood decay, and also that mycobiomes between treated wood and natural log would be significantly different because of the following: (1) Natural logs of many tree species are inherently susceptible to decay and often possess limited structural durability, thereby requiring preservative treatment to improve longevity [6,7]. (2) Outdoor wood structures usually undergo a wide range of modifications, including pretreatment with fungitoxic or fungistatic preservatives such as copper- or iron-containing compounds [6], as well as thermomechanical processing methods involving steam, heat, ammonia, cold rolling, densification, or partial removal of lignin and hemicellulose to facilitate densification [114]. Our unexpected finding that natural logs and processed wood collected from the same locations did not exhibit clear separation in fungal community composition (Section 3.4, Fungal Community Composition Among Substrate Types) may suggest limited long-term effectiveness of current wood-processing approaches in preventing fungal colonization. Another surprise was the general lack of widely dominant basidiomycete fungi for outdoor wood decay. The association of a large number of ascomycetes with outdoor wood decay would challenge the wood industry to develop physical/chemical or biological treatments that would need to be effective against a broad spectrum of possible wood-decay fungal taxa. However, our tentative conclusion is constrained by the lack of information regarding the prior treatment history of many processed wood samples, as many structures had been installed for years or were collected opportunistically without documentation. This uncertainty regarding treatment history limits our ability to interpret fungal community profiles in relation to any specific treatment or processing method. Variation among processing methods may therefore represent an additional source of fungal community heterogeneity. Furthermore, prolonged outdoor exposure to moisture may promote preservative leaching, thereby reducing treatment efficacy over time [6].

Our study identified several fungal genera widely dominant in processed wood samples across multiple locations without experimental evidence of involvement in wood decay. Genera *Anteaglonium* Mugambi & Huhndorf (three locations), *Teratosphaericola* Quaedvl. & Crou (four locations), and *Tolypocladium* W. Gams (three locations) have been reported as endophytes of living trees, soil-associated fungi, or saprotrophs of naturally decaying wood rather than as dominant fungi in processed or engineered wood structures [95,115,116,117]. Several explanations may account for this unexpected finding. Firstly, processed wood materials may become contaminated with fungal spores during manufacturing or storage [118], although this alone does not explain the absence of these genera from natural log samples in our dataset. Two additional explanations appear more plausible in our context. Adhesives used in processed wood products may contain readily available carbohydrates that serve as nutrient sources for certain fungi, thereby enhancing fungal survival and colonization [118]. Secondly, some fungi have a natural resistance against copper-based preservatives [119]; known examples include species of *Trichaptum* and several soft-rot ascomycetes [120]. Such resistance may alter fungal community composition in treated wood by reducing biotic competition and allowing copper-resistant fungi to proliferate. Targeted in vitro decay assays using copper-treated wood blocks would help to evaluate these hypotheses. These processed wood-enriched fungal taxa can be targeted for optimizing wood treatment once their role in wood decay is confirmed.

In our previous metagenomic survey of diseased living urban trees in Singapore, several white- and brown-rot basidiomycete genera and one soft-rot ascomycete genus, *Scytalidum* Pesante, were shortlisted as significant pathogenic fungi for urban trees [10] (summarized in Table S2). Another notable observation was that samples from the same location were frequently dominated by the same pathogenic fungal taxa. Unlike the situation of pathogenic fungi in living trees, in our present decayed wood dataset, most of these genera were not detected (*Flavodon* Ryvarden, *Fomitiporia* Murrill, *Fomitopsis* P. Karst., *Meripilus* P. Karst., *Pseudofibroporia* Yuan Y. Chen & B.K. Cui, *Serpula* (Pers.) Gray, and *Tomophagus* Murrill) at all or were detected only at very low relative abundances (*Fulvifomes* Murrill, *Ganoderma* P. Karst., *Phellinus* Quél., *Rigidoporus* Murrill, and *Scytalidium*). This contrasting finding seems to suggest a distinctiveness between pathogenic fungi and saprophytic fungi in tropical Singapore. The exception was *Perenniporia* Murrill, which was listed as a pathogenic fungus but also detected in decayed wood structures at three sites, suggesting its possible facultative lifestyle. Furthermore, in our present study of wood decay, there is a general lack of strong local dominance by a single fungal taxon across samples within a single site. Only few taxa, such as *Trichaptum byssogenum* in JGHA samples, *Exophiala* sp. in CC samples, and *Peniophorella* sp. in JGHB samples, were consistently detected across samples from the same location.

## 5. Conclusions

This study provides a broad survey of fungal communities colonizing decayed natural logs and outdoor wood structures across 15 sites in Singapore, revealing a high level of fine-scale heterogeneity in the fungal community both within and among locations. Together, these findings suggest that macro-factors such as sampling location and substrate type exert only limited influence on fungal community composition. Even samples collected centimeters apart from the same wood piece frequently exhibited highly dissimilar communities, indicating that fungal community assembly is largely stochastic and likely driven by microscale environmental variation and biotic interactions.

Although the overall abundances of *Ascomycota* and *Basidiomycota* were comparable, ascomycetes were strongly represented among the dominant fungal genera. The widely distributed dominant genera detected across multiple locations were mostly ascomycetes, suggesting that this phylum may play a more important role in wood biodegradation under tropical conditions than is currently recognized. In vitro wood-decay assays further demonstrated the independent degradative capability of locally relevant wood species by an ascomycete fungal isolate of *Paecilomyces maximus*, reinforcing the potential ecological importance of ascomycetes in tropical wood decomposition.

Future studies should refine wood-decay assays by systematically evaluating the effects of various factors such as preservative treatment, wood type, moisture content, and pH. Further research is also needed to clarify the roles of ascomycetes in wood deterioration and the mechanisms underlying their decay activity. Such investigations could improve predictions of treated-wood durability under tropical climatic conditions and support the development of more effective antifungal strategies for wood-based infrastructure and construction materials.

## Figures and Tables

**Table 1:** Summary of sample collections.

Sample Site (Code)	Location Type	Natural Log Samples	Processed Wood Samples
School of Biological Sciences, Level 2 Open Area (SBS)	University Campus	0	6
National Institute of Education Level 1 Open Area (NIE)	University Campus	0	11
Singapore Zoo (SZ)	Public Zoo Facility	0	4
Night Safari (NS)	Public Zoo Facility	0	9
Bukit Timah Nature Reserve (BTNR)	Nature Reserve	7	3
Gaia (GAIA)	University Campus	0	6
Nanyang Lake (NYL)	University Campus	0	5
Labrador Nature Reserve (LNR)	Nature Reserve	8	6
Jurong Lake Gardens (JLG)	Public Park	24	0
Calvary Carpenter (CC)	Private Residence	0	6
Jalan Greja House-A (JGHA)	Private Residence	0	12
West Coast Park (WCP)	Public Park	0	12
Taman Bedok–Jalan Greja Neighborhood (TBJG)	Residential Neighborhood	4	4
Taman Bedok House (TBH)	Private Residence	0	3
Jalan Greja House-B (JGHB)	Private Residence	0	3

**Table 2:** Fungal composition comparison among selected samples from BTNR and WCP exhibiting low Bray–Curtis dissimilarity values.

Sample ID	BTNR2.1	WCP2.1	WCP9.1
Substrate Type	Processed Wood	Processed Wood	Processed Wood
**Genus Taxonomy**			
*Anteaglonium*	72.15%	69.01%	83.72%
*Knufia*	9.08%	0.45%	0%
*Pseudodactylaria*	0.76%	1.70%	1.52%
*Neospadicoides*	0.71%	9.20%	0%
*Pseudocastanedospora*	<0.01%	0.69%	5.33%
	**BTNR9.1**	**WCP8.1**	
**Substrate_Type**	**Natural Log**	**Processed Wood**	
**Genus Taxonomy**			
*Peniophorella*	83.71%	97.11%	
*Nothophaeomoniella*	1.02%	0.16%	
**Species Taxonomy**			
*Peniophorella crystallifera*	0%	97.79%	
*Peniophorella pallida*	83.66%	0%	
*Nothophaeomoniella ekebergiae*	1.02%	0.16%	

Bold text denotes table headings.

**Table 3:** Bray–Curtis dissimilarities between samples collected from the same wood pieces.

Sample Pairs (Wood Structure/Log)	Bray–Curtis Pairwise Dissimilarity Values
NIE1.1, NIE2.1 (replicates)	0.03
SBS1.1, SBS1.2 (wood structure)	0.8
SBS3.1, SBS3.2 (wood structure)	0.99
NIE3.1, NIE3.2 (wood structure)	0.63
NIE6.1, NIE6.2 (wood structure)	0.91
NIE7.1, NIE7.2 (wood structure)	0.99
NS1.1, NS1.3 (wood structure)	0.69
BTNR6.1, BTNR6.2 (log)	1
NYL1.1, NYL1.2 (wood structure)	0.5
LNR11.1, LNR11.2 (wood structure)	0.24
JLG1.1, JLG1.2 (log)	0.71
JLG1.1, JLG1.3 (log)	0.83
JLG1.1, JLG1.4 (log)	1
JLG1.2, JLG1.3 (log)	0.99
JLG1.2, JLG1.4 (log)	1
JLG1.3, JLG1.4 (log)	0.98
CC1.1, CC1.2 (wood structure)	0.88
CC2.1, CC2.2 (wood structure)	0.77
CC3.1, CC3.2 (wood structure)	0.32
JGHA7.1, JGHA7.2 (wood structure)	0.01
JGHA7.1, JGHA7.3 (wood structure)	0.01
JGHA7.2, JGHA7.3 (wood structure)	0.02
JGHA9.1, JGHA9.2 (wood structure)	0.07
WCP4.1, WCP4.2 (wood structure)	0.99
WCP7.1, WCP7.2 (wood structure)	0.9
TBJG9.1, TBJG9.2 (log)	0.56

**Table 4:** Summary of dominant fungal genera detected and published experimental evidence for their involvement in wood decay.

*Ascomycota*		
Genus	No. of Locations	Reported Evidence for Wood Decay
*Albicollum*	2	None
*Angustimassarina*	1	None
*Anteaglonium* #2	3	Activity shown on carboxymethyl cellulose and ABTS agar, indicating cellulase and oxidative activity [33]
*Aspergillus* #2	1	Wood cell wall damage under moist laboratory conditions [34,35]
*Cataractispora*	2	None
*Ceramothyrium*	1	No cellobiose dehydrogenase activity detected in *C. linneae* or *C. thermophilum* [36]
*Ceratostomella*	2	None
*Chaetomium*	1	Wood mass loss and soft-rot damage by *C. globosum*; cellobiose dehydrogenase activity in *C. atrobrunneum* and *C. cellulolyticum* [36,37]
*Chaetosphaeria*	1	None
*Chaetothyrium*	1	None
*Chalarosphaeria*	4	None
*Chloridium*	1	None
*Cladophialophora* #2	6	None
*Cladosporium* #2	1	Laboratory decay potential, but no significant field stump decay [38]
*Conioscypha*	1	Cellulase-related activities and soft-rot cavities in balsa wood by *C. varius* [39]
*Cordana*	1	None
*Cosmospora*	2	None
*Devriesia*	1	None
*Diluviicola*	1	None
*Exophiala* #2	7	Cellulase production by *E. zingiberis* [40]
*Fonsecaea*	1	None
*Gloniopsis*	1	None
*Hawksworthiomyces* #2	2	Lignocellulolytic activity and wood-block decay by *H. lignivorus* [41,42]
*Helicosporium*	2	An isolate produced cellulase and xylanase and was associated with an 8.46 ± 3.08% wood mass loss [43]
*Humicola*	1	Cellobiose dehydrogenase activity in *H. insolens* [36]
*Hypomyces*	1	None
*Kirschsteiniothelia*	1	*H. alopallonella* formed cavities on birch wood cell walls [44]
*Knufia*	1	Cellulase activity on test medium [45]
*Lasiodiplodia*	1	Up to 12.2% rubberwood mass loss and cell wall erosion under the older name *Botryodiplodia theobromae* [46]
*Lignosphaeria*	1	None
*Medicopsis* #2	2	Decaying wood associated with isolate showing protease, lipase, and chitinase activity [47]
*Neoleptodontidium* #2	1	None
*Neomorinagamyces*	1	None
*Neophaeomoniella*	1	None
*Neospadicoides*	1	None
*Nothophaeomoniella*	2	None
*Paracladophialophora*	1	None
*Parahelicomyces*	1	None
*Pectenia*	1	None
*Penicillium* #2	1	Type-II soft-rot-like erosion of pine and sycamore wood by *P. chrysogenum*; cellulose hydrolysis by other isolates [34]
*Phaeoacremonium* #2	1	Lignin peroxidase, manganese peroxidase, and laccase detected in grapevine wood isolates [48]
*Phialemonium*	1	None
*Phialophora*	1	Caused soft-rot cavities in wood [49]
*Phialoseptomonium*	1	None
*Pichia*	1	None
*Pleurotheciella*	1	None
*Pleurothecium*	2	None
*Pseudocastanedospora*	2	None
*Pseudodactylaria*	5	None
*Ramophialophora*	1	None
*Rhamphoria* #2	1	None
*Rhexodenticula*	4	None
*Rhinocladiella*	2	None
*Scytalidium* #2	2	Significant mass loss in decayed beech wood, but not sound beech wood, caused by *S. lignicola*; eight carbohydrate esterase family 1 and eight glycoside hydrolase family 43 proteins in *S. thermophilum* [50,51]
*Spadicoides*	2	None
*Sporidesmium*	4	None
*Talaromyces* #2	2	Cellulases and xylanases from *T. cellulolyticus* [52]
*Teratosphaeria*	1	None
*Teratosphaericola*	4	None
*Thyridium* #2	1	None
*Tolypocladium*	3	None
*Trichoderma* #2	4	*T. hamatum*, *T. harzianum* and *T. koningii* caused significant mass loss in decayed beech wood, but rarely in sound wood [50]
*Tympanis*	2	Small mass loss in pine wood blocks by *T. hypopodia* [53]
*Valsonectria*	1	None
*Vanakripa*	2	None
*Veronaea*	1	None
*Verrucaria*	2	None
*Vertexicola*	2	None
*Vexillomyces*	1	None
*Waltergamsia*	1	None
*Xenomyrothecium*	1	None
*Xylolentia*	1	None
*Zanclospora*	1	None
***Basidiomycota***		
**Genus**	**No. of Locations**	**Reported evidence for wood decay**
*Candolleomyces*	2	None
*Cerinomyces*	2	Substantial wood decay by *C. ceraceus* [54]
*Ceriporia*	1	Pine wood composition change and mass loss by *C. lacerata* [55]
*Clitocybula*	1	Brown-rot characteristics reported for *C. canariensis* [56]
*Coniophora*	1	*C. puteana* is a brown-rot fungus causing substantial mass loss in European beech and Scots pine sapwood [57,58]
*Coprinellus*	1	Additional mass loss of previously field-decayed wood blocks [59]
*Coprinopsis*	1	No wood-decay ability shown [60]
*Flavodon*	1	*F. flavus* degraded lignin in *Mangifera* and *Syzygium* wood [61]
*Ganoderma* #1	2	Wood and lignin loss, delignification, mottled rot, and ligninolytic enzymes [62,63,64,65]
*Gerronema*	1	None
*Gloeodontia*	1	None
*Gymnopilus*	3	Cellulose and lignin decomposition by *G. pampeanus* [66]
*Gymnopus*	1	None
*Hydnum*	1	None
*Hyphoderma*	3	Wood-inhabiting white-rot fungi [67]
*Lentinus*	3	Lignin-modifying enzyme screening of wood-derived isolates [68]
*Mycena*	1	*M. haematopoda* caused substantial loss of undecayed beech wood [50]
*Peniophorella*	7	Found to cause white rot and substantial mass loss in sweetgum and white fir wood [69,70]
*Perenniporia* #1	3	Oak and beech wood degradation and ligninolytic enzymes [63,65,71]
*Phanerochaete* #2	2	Wood-derived lignin mineralization and lignin, xylose, and glucose loss [63,72]
*Phlebiopsis* #2	1	Pine-log colonization and modification by *P. gigantea* [73]
*Picipes* #1	1	White-rot fungi on angiosperm and gymnosperm wood [74]
*Punctulariopsis*	2	None
*Scytinostroma*	1	Selective lignin removal from wood by *S. galactinum* [75]
*Serendipita*	2	None
*Stypella*	1	None
*Subulicystidium*	2	None
*Trametes* #1	1	White-rot erosion, wood mass loss, lignin and sugar loss, and ligninolytic enzymes caused by *T. versicolor* [63,76]
*Trichaptum* #1#2	3	Beech and spruce decomposition and lignocellulolytic enzymes [77,78]
*Tulasnella*	1	*T. albida* had negative oxidase and lignin peroxidase assays [79] but the genome sequence of *T. calospora* was determined and it was found that genes encoding ligninolytic class II peroxidases and laccase were lost in *Tulasnella* genera during their evolution [80]
*Vuilleminia*	3	Extensive white rot of oak branches by *V. comedens* [81]
*Xylodon*	3	Primarily white-rot decomposers [82]
***Mucoromycota***		
**Genus**	**No. of locations**	**Reported evidence for wood decay**
*Rhizopus*	1	None

#1: with adjoining fruiting body molecularly identified as the same taxon; #2: with culture isolates; Bold text denotes table headings.

## Data Availability

More data supporting the reported results can be found in the Supplementary Materials.

## Supplementary Materials

The following supporting information can be downloaded at https://www.mdpi.com/article/10.3390/jof12100754/s1: Section S1. Shiny application details; Section S2. Statistical analysis and plot details; Section S3. Supplementary figures and tables: Table S1: Phylum composition at individual locations; Table S2: Occurrence and relative abundances of pathogenic wood-decay fungal genera previously identified from diseased urban trees, as detected in the present deadwood metagenomic survey; Table S3: Summary statistics of within-area Bray–Curtis dissimilarities; Table S4: Summary of dominant fungal genera detected across all samples and their associated sampling locations and substrate types; Table S5: Widely distributed dominant ascomycete genera, their sampling locations, and reported ecological roles; Figure S1: Beta diversity heatmap showing Bray-Curtis dissimilarity between fungal communities at a genus level across all locations (excluding inconclusive genera); Figure S2: Beta diversity heatmap showing Bray-Curtis dissimilarity between fungal communities at a genus level across all samples in SBS; Figure S3: Beta diversity heatmap showing Bray-Curtis dissimilarity between fungal communities at a genus level across all samples in NIE; Figure S4: Beta diversity heatmap showing Bray-Curtis dissimilarity between fungal communities at a genus level across all samples in SZ; Figure S5: Beta diversity heatmap showing Bray-Curtis dissimilarity between fungal communities at a genus level across all samples in NS; Figure S6: Beta diversity heatmap showing Bray-Curtis dissimilarity between fungal communities at a genus level across all samples in BTNR; Figure S7: Beta diversity heatmap showing Bray-Curtis dissimilarity between fungal communities at a genus level across all samples in GAIA; Figure S8: Beta diversity heatmap showing Bray-Curtis dissimilarity between fungal communities at a genus level across all samples in NYL; Figure S9: Beta diversity heatmap showing Bray-Curtis dissimilarity between fungal communities at a genus level across all samples in LNR; Figure S10: Beta diversity heatmap showing Bray-Curtis dissimilarity between fungal communities at a genus level across all samples in JLG; Figure S11: Beta diversity heatmap showing Bray-Curtis dissimilarity between fungal communities at a genus level across all samples in CC; Figure S12: Beta diversity heatmap showing Bray-Curtis dissimilarity between fungal communities at a genus level across all samples in JGHA; Figure S13: Beta diversity heatmap showing Bray-Curtis dissimilarity between fungal communities at a genus level across all samples in WCP; Figure S14: Beta diversity heatmap showing Bray-Curtis dissimilarity between fungal communities at a genus level across all samples in TBJG; Figure S15: Beta diversity heatmap showing Bray-Curtis dissimilarity between fungal communities at a genus level across all samples in TBH; Figure S16: Beta diversity heatmap showing Bray-Curtis dissimilarity between fungal communities at a genus level across all samples in JGHB; Figure S17: Beta diversity heatmap showing Bray-Curtis dissimilarity between fungal communities at a genus level in samples from West Coast Park and Bukit Merah Nature Reserve (excluding in-conclusive genera); Figure S18: Bar plot of genera with >1% relative abundance in SBS samples; Figure S19: Bar plot of genera with >1% relative abundance in NIE samples; Figure S20: Bar plot of genera with >1% relative abundance in SZ samples; Figure S21: Bar plot of genera with >1% relative abundance in NS samples; Figure S22: Bar plot of genera with >1% relative abundance in BTNR samples; Figure S23: Bar plot of genera with >1% relative abundance in GAIA samples.; Figure S24: Bar plot of genera with >1% relative abundance in NYL samples; Figure S25: Bar plot of genera with >1% relative abundance in LNR samples; Figure S26: Bar plot of genera with >1% relative abundance in JLG samples; Figure S27: Bar plot of genera with >1% relative abundance in CC samples; Figure S28: Bar plot of genera with >1% relative abundance in JGHA samples; Figure S29: Bar plot of genera with >1% relative abundance in WCP samples; Figure S30: Bar plot of genera with >1% relative abundance in TBJG samples; Figure S31: Bar plot of genera with >1% relative abundance in TBH samples; Figure S32: Bar plot of genera with >1% relative abundance in JGHB samples; Figure S33: NMDS ordination of fungal community composition based on Bray-Curtis dissimilarity for all wood samples across 15 locations in Singapore (stress = 0.273). Ellipses represent 95% confidence intervals for each location.
