# Supplementary material for: Amplicon-Based Profiling of Fungal Communities in Decayed Outdoor Wood Structures and Natural Logs in Tropical Singapore
Source: J Fungi (Basel). 2026 Oct 8;12(10):754. doi: 10.3390/jof12100754 (PMC13706489; doi:10.3390/jof12100754)
Supplement: Supplementary file 1 [file jof-12-00754-s001.zip › jof-4521899-supplementary.pdf]

Section S1. Shiny application details

*S1.1 Shiny application interface*

All analyses and plots are implemented in an interactive R Shiny application available at https://jerricasng.shinyapps.io/shiny_app/ or on Github https://github.com/jerricasng-dotcom/Fungi-Metagenomic-Analysis.git.

Test files (Metadata, Batch percentages, Batch ASV sequence, BLAST hits, Combined-post-BLAST) files are also uploaded on Github to demonstrate the expected file formats and allow users to test the Shiny application. Instructions are given in the README file on Github.

*S1.2 Brief instructions on how to use Shiny application*

Main tab:

1. Upload 'TEST Batch Percentage File.xlsx' into 'Percentage file' under 'Option 2: Add New Batches', and 'TEST Batch ASV Sequence.txt' into 'Sequence FASTA'. Set the batch tag to B1.
2. Click 'Save last batch'. If you have more batches, click 'Add next batch' and repeat for each batch.
3. When all batches are added, click “Run all”.
4. You can now download the combined tables and generate “unknowns” at different taxonomic levels.
5. Because NCBI BLAST may not efficiently handle very large ASV batches, splitting sequences by batch is recommended; this can be done from the Main tab using the sequence-splitting options.

BLAST results tab

1. After running BLAST on NCBI, download the BLAST hit table as a CSV file and upload it under 'Upload BLAST hit tables (csv/xlsx, multiple batches)'. You may select multiple files if needed.
2. If available, enter your NCBI API key, set the minimum percentage identity and maximum E-value, then click 'Best Hit Assignments' followed by 'Download Best-Hit assignments' to obtain one best hit per ASV.
3. If a 'taxonomy_for_accessions.xlsx' file is already available, you can upload it so that existing taxonomy is reused and NCBI lookups are not repeated.
4. Under 'Mark ReBLASTed ASVs', click 'All Combined After BLAST' and then 'Download All Combined After BLAST' to save a combined post-BLAST table for future visualization and analysis.

Missing ASV Checker tab

This tab is provided because large BLAST submissions may occasionally fail to return all queries. It compares the original list of ASV IDs with those in the uploaded BLAST hit tables, reports missing ASVs, and re-exports their sequences as FASTA for follow-up analysis. No test files are provided for this tab as it is not essential for basic use.

Visualization & Analysis tab

1. Choose the data source as either: 'Use combined results from BLAST tab', or an Excel file previously downloaded via 'Download All Combined After BLAST'.
2. For testing, upload 'TEST Combined AFTER BLAST.xlsx' under 'Data source' and click 'Load Data'.
3. In the Main tab, upload 'TEST Sample Meta Data.xlsx' under 'Upload metadata (csv/xlsx)'. You can then run visualization and analysis (e.g. taxonomic composition plots, diversity metrics, ordinations, and PERMANOVA).

## Section S2. Statistical analysis and plot details

### *S2.1 Taxonomic composition plots*

Taxonomic composition was summarised at multiple ranks (phylum to species) using relative-abundance stacked bar plots and pie charts, optionally consolidating low-abundance taxa below a user-defined threshold into an "Others" category. Taxa annotated as "Unknown" could be excluded from these plots, because they consist of a heterogenous mixture of ASVs that likely represent multiple, distinct but inconclusive taxa, and aggregating them into a single "Inconclusive" group would introduce disproportionate uncertainty and potential bias into the visualised community profiles.

### *S2.2 Alpha diversity*

Alpha diversity was calculated directly from ASV relative-abundance tables provided by Novogene metagenomic sequencing service. For each sample, richness was computed as the number of ASVs with non-zero relative abundance. Observed ASV richness, Shannon, and Simpson indices were computed on these proportional data using functions from the vegan package (vegan::diversity) with index = "shannon" and index = "simpson", respectively.

### Beta Diversity and non-metric ordination

Bray-Curtis dissimilarity matrices were calculated from ASV relative-abundance data using vegan::vegdist to characterise pairwise differences in community composition between samples. These matrices were used to generate distance heatmaps, hierarchical clustering trees, nonmetric multidimensional scaling (NMDS) ordinations (vegan::metaMDS) and within-group Bray-Curtis dissimilarity boxplots. Group level differences, either between areas or sample types, were tested using permutational multivariate analysis of variance PERMANOVA (vegan::adonis2) on Bray-Curtis distance matrices with 999 permutations.

### *S2.3 Principal component analysis*

Principal component analysis (PCA) was performed with the base prcomp function on Euclidean distance-based representations of the community data to summarise major gradients in composition. PCA scores were visualised with samples coloured by metadata categories such as sampling area or sample type. Group-level differences, either between areas or between sample types, were tested using PERMANOVA (vegan::adonis2, 999 permutations) applied to Euclidean distance matrices.

### *S2.4 Phylum-level composition analysis*

A phylum-level analysis was used to examine the internal composition of selected phyla in more detail. For a user-chosen phylum, the application generates pie charts showing the relative abundances of subordinate taxa at a specific rank (class, order, family, genus, or species), across all areas, a single area, or a user-defined subset of areas, with groups below a user-defined percentage threshold aggregated into an "Others" category.

Section S3. Supplementary figures and tables

*Section S3.1 Tables*

**Table S1.** Phylum composition at individual locations.

| Location | No. Of Samples | % of Ascomycota | % of Basidiomycota | % of Inconclusive Phyla | % of Other Phyla |
|---|---|---|---|---|---|
| CC | 6 | 87.51 | 3.28 | 9.09 | 0.08 |
| GAIA | 6 | 84.09 | 7.41 | 8.08 | 0.43 |
| TBH | 3 | 67.70 | 31.83 | 0.00 | 0.47 |
| JGHB | 3 | 58.88 | 32.05 | 9.07 | 0.00 |
| NIE | 11 | 57.81 | 33.01 | 8.84 | 0.33 |
| TBJG | 8 | 57.59 | 37.08 | 5.26 | 0.08 |
| WCP | 12 | 55.78 | 37.70 | 4.60 | 1.92 |
| LNR | 14 | 48.71 | 37.37 | 13.47 | 0.45 |
| JGHA | 12 | 2.16 | 96.22 | 2.16 | 0.01 |
| SZ | 4 | 17.67 | 49.12 | 33.16 | 0.05 |
| JLG | 24 | 38.59 | 44.81 | 16.23 | 0.37 |
| NYL | 5 | 34.16 | 41.98 | 23.69 | 0.17 |
| SBS | 6 | 36.49 | 29.33 | 34.06 | 0.11 |
| BTNR | 10 | 41.29 | 44.39 | 14.29 | 0.04 |
| NS | 9 | 39.90 | 36.95 | 22.64 | 0.51 |

**Table S2.** Occurrence and relative abundances of pathogenic wood-decay fungal genera previously identified from diseased urban trees, as detected in the present deadwood metagenomic survey.

| Genus | Phylum | Relative abundance per site |
|---|---|---|
| *Flavodon* | Basidiomycota | Not detected |
| *Fomitiporia* | Basidiomycota | Not detected |
| *Fomes / Fomitopsis* | Basidiomycota | Not detected |
| *Meripilus* | Basidiomycota | Not detected |
| *Pseudofibroporia (related to Fibroporia)* | Basidiomycota | Not detected |
| *Serpula* | Basidiomycota | Not detected |
| *Tomophagus (formerly Ganoderma)* | Basidiomycota | Not detected |
| *Fulvifomes* | Basidiomycota | WCP (0.016%) |
| *Ganoderma* | Basidiomycota | BTNR (3.625%), GAIA (0.102%), WCP (0.041%), SBS (0.015%), NIE (0.003%) |
| *Perenniporia* | Basidiomycota | SBS (16.542%), NS (0.016%), SZ (0.023%), GAIA (0.02%), NIE (0.004%), JLG (0.002%) |
| *Phellinus* | Basidiomycota | NIE (< 0.001%) |

| | | |
|---|---|---|
| *Rigidoporus* | Basidiomycota | JLG (0.002%) |
| *Scytalidium* | Ascomycota | NIE (0.648%), NS (0.208%), WCP (0.023%), TBJG (0.017%) |

Bold text denotes table headings.

**Table S3.** Summary statistics of within-area Bray–Curtis dissimilarities

| Area | n-pairs | Median | Q1 | Q3 | IQR | Min | Max | Range |
|---|---|---|---|---|---|---|---|---|
| SBS | 15 | 0.930 | 0.874 | 0.995 | 0.120 | 0.802 | 0.999 | 0.196 |
| NIE | 55 | 0.962 | 0.919 | 0.987 | 0.068 | 0.029 | 0.998 | 0.969 |
| SZ | 6 | 0.986 | 0.977 | 0.992 | 0.015 | 0.958 | 0.996 | 0.038 |
| NS | 36 | 0.947 | 0.866 | 0.976 | 0.110 | 0.668 | 0.999 | 0.330 |
| BTNR | 45 | 0.996 | 0.985 | 0.998 | 0.014 | 0.646 | 1.000 | 0.354 |
| GAIA | 15 | 0.977 | 0.891 | 0.990 | 0.099 | 0.678 | 1 | 0.322 |
| NYL | 10 | 0.962 | 0.906 | 0.992 | 0.085 | 0.504 | 1 | 0.496 |
| LNR | 91 | 0.986 | 0.972 | 0.992 | 0.020 | 0.244 | 0.999 | 0.755 |
| JLG | 276 | 0.989 | 0.956 | 0.996 | 0.040 | 0.011 | 1 | 0.989 |
| CC | 15 | 0.768 | 0.663 | 0.907 | 0.244 | 0.295 | 0.959 | 0.664 |
| JGHA | 66 | 0.024 | 0.014 | 0.045 | 0.031 | 0.003 | 0.087 | 0.084 |
| WCP | 66 | 0.977 | 0.924 | 0.987 | 0.063 | 0.269 | 0.999 | 0.731 |
| TBJG | 28 | 0.999 | 0.992 | 1 | 0.008 | 0.565 | 1 | 0.435 |
| JGHB | 3 | 0.635 | 0.490 | 0.721 | 0.231 | 0.344 | 0.806 | 0.462 |
| TBH | 3 | 0.971 | 0.695 | 0.986 | 0.291 | 0.419 | 1 | 0.581 |

**Table S4.** Summary of dominant fungal genera detected across all samples and their associated sampling locations and substrate types.

| ***Ascomycota*** | | | |
|---|---|---|---|
| **Genus** | **Processed Wood** | **Natural Log** | **No. of Locations** |
| *Albicollum* | LNR9.1, JGHB1.1, JGHB2.1 | | 2 |
| *Angustimassarina* | GAIA3.1 | | 1 |
| *Anteaglonium* | BTNR2.1, BTNR3.1, NYL3.1, WCP2.1, WCP9.1 | | 3 |
| *Aspergillus* | WCP4.2 | | 1 |
| *Cataractispora* | CC2.1, CC3.1, CC3.2 | JLG4.1 | 2 |
| *Ceramothyrium* | | BTNR5.1 | 1 |
| *Ceratostomella* | NYL1.2, NYL4.1 | JLG19.1 | 2 |
| *Chaetomium* | | LNR7.2 | 1 |
| *Chaetosphaeria* | LNR14.1 | | 1 |
| *Chaetothyrium* | | JLG13.1 | 1 |
| *Chalarosphaeria* | CC1.1, WCP7.1 | BTNR5.1, TBJG8.1 | 4 |
| *Chloridium* | NS1.1 | | 1 |
| *Cladophialophora* | SZ3.1, SZ4.1, NS2.1, NS3.1, NS7.3, NS8.1, WCP4.1, WCP7.1, WCP7.2, TBJG4.1 | LNR2.1, LNR4.1, LNR5.1, LNR6.1, JLG1.1, JLG1.3, JLG10.1, JLG19.1, JLG20.1, JLG21.1, TBJG8.1 | 6 |
| *Cladosporium* | GAIA2.1 | | 1 |
| *Conioscypha* | NYL3.1 | | 1 |

| | | | |
|---|---|---|---|
| *Cordana* | GAIA7.1 | | 1 |
| *Cosmospora* | GAIA1.1, WCP4.2 | | 2 |
| *Devriesia* | NYL2.1 | | 1 |
| *Diluviicola* | TBJG6.1 | | 1 |
| *Exophiala* | SBS1.1, NIE7.1, GAIA1.1, GAIA5.1, NYL4.1, LNR9.1, CC1.1, CC1.2, CC2.1, CC3.1, CC3.2 | JLG19.1, JLG21.1 | 7 |
| *Fonsecaea* | LNR14.1 | | 1 |
| *Gloniopsis* | GAIA5.1 | | 1 |
| *Hawksworthiomyces* | NIE8.1, CC2.1, CC2.2 | | 2 |
| *Helicosporium* | NS3.1 | JLG7.1 | 2 |
| *Humicola* | SBS2.1 | | 1 |
| *Hypomyces* | | LNR3.1 | 1 |
| *Kirschsteiniothelia* | WCP7.2 | | 1 |
| *Knufia* | NS7.3 | | 1 |
| *Lasiodiplodia* | | TBJG9.1, TBJG9.2 | 1 |
| *Lignosphaeria* | BTNR3.1 | | 1 |
| *Medicopsis* | GAIA3.1, GAIA4.1, GAIA5.1 | JLG6.1 | 2 |
| *Neoleptodontidium* | NS8.1 | | 1 |
| *Neomorinagamyces* | GAIA7.1 | | 1 |
| *Neophaeomoniella* | WCP1.1 | | 1 |
| *Neospadicoides* | NYL4.1 | | 1 |
| *Nothophaeomoniella* | NIE7.1, WCP6.1 | | 2 |
| *Paracladophialophora* | | LNR7.2 | 1 |
| *Parahelicomyces* | CC1.2 | | 1 |
| *Pectenia* | NIE1.1 (Duplicate), NIE2.1 (Duplicate) | | 1 |
| *Penicillium* | WCP3.1 | | 1 |
| *Phaeoacremonium* | GAIA3.1, GAIA5.1 | | 1 |
| *Phialemonium* | NIE6.1 | | 1 |
| *Phialophora* | | JLG6.1 | 1 |
| *Phialoseptomonium* | | JLG12.1 | 1 |
| *Pichia* | | JLG1.4 | 1 |
| *Pleurotheciella* | GAIA4.1 | | 1 |
| *Pleurothecium* | | BTNR6.1, LNR4.1, LNR5.1 | 2 |
| *Pseudocastanedospora* | NS1.1, NS1.3, NS6.3, NS7.3, WCP10.1 | | 2 |
| *Pseudodactylaria* | NS7.3, LNR12.1, TBJG4.1, TBH3.1 | BTNR6.1 | 5 |
| *Ramophialophora* | GAIA7.1 | | 1 |
| *Rhamphoria* | WCP5.1 | | 1 |
| *Rhexodenticula* | SBS3.1, SZ4.1, NS6.3 | LNR5.1 | 4 |
| *Rhinocladiella* | GAIA5.1, WCP10.1 | | 2 |

| | | | |
|---|---|---|---|
| *Scytalidium* | BTNR4.1 | BTNR9.1, JLG1.3, JLG13.1, JLG20.1 | 2 |
| *Spadicoides* | WCP3.1 | JLG3.1, JLG7.1 | 2 |
| *Sporidesmium* | NIE1.1 (Duplicate), NIE2.1 (Duplicate), CC1.1, CC2.2, TBJG7.1 | JLG1.1, JLG10.1, JLG15.1 | 4 |
| *Talaromyces* | SZ3.1, BTNR4.1 | | 2 |
| *Teratosphaeria* | TBH1.1 | | 1 |
| *Teratosphaericola* | NIE4.1, NS8.1, NYL2.1, WCP3.1 | | 4 |
| *Thyridium* | TBJG6.1 | | 1 |
| *Tolypocladium* | SBS1.1, NS2.1, WCP1.1 | | 3 |
| *Trichoderma* | NIE5.1, WCP4.1 | LNR3.1, JLG6.1, JLG13.1 | 4 |
| *Tympanis* | NS2.1 | LNR6.1 | 2 |
| *Valsonectria* | SBS1.1 | | 1 |
| *Vanakripa* | | LNR3.1, JLG10.1 | 2 |
| *Veronaea* | | JLG1.1, JLG1.2 | 1 |
| *Verrucaria* | NIE5.1, NIE7.1, CC1.1 | | 2 |
| *Vertexicola* | SBS3.1 | JLG4.1 | 2 |
| *Vexillomyces* | | LNR6.1 | 1 |
| *Waltergamsia* | | TBJG2.1 | 1 |
| *Xenomyrothecium* | SBS3.2 | | 1 |
| *Xylolentia* | WCP4.1, WCP5.1 | | 1 |
| *Zanclospora* | NS5.1 | | 1 |
| ***Basidiomycota*** | | | |
| **Genus** | **Processed Wood** | **Natural Log** | **No. of Locations** |
| *Candolleomyces*#1 | | JLG1.1, TBJG9.1 | 2 |
| *Cerinomyces* | NYL2.1, TBJG4.1 | | 2 |
| *Ceriporia* | | LNR3.1, LNR4.1 | 1 |
| *Clitocybula* | | BTNR1.1 | 1 |
| *Coniophora* | NIE4.1 | | 1 |
| *Coprinellus* | | JLG1.2 | 1 |
| *Coprinopsis* | | TBJG2.1, TBJG9.2 | 1 |
| *Flavodon* | | JLG17.2 | 1 |
| *Ganoderma* | | BTNR8.2, JLG5.2 | 2 |
| *Gerronema* | NS5.1 | | 1 |
| *Gloeodontia* | | BTNR6.2 | 1 |
| *Gymnopilus* | LNR14.1, WCP10.1 | JLG1.3 | 3 |
| *Gymnopus* | SZ2.1 | | 1 |
| *Hydnum* | | JLG20.1 | 1 |
| *Hyphoderma* | TBH2.1, TBH3.1, JGHB2.1 | BTNR7.2 | 3 |
| *Lentinus* | NS4.1, NYL3.1 | JLG9.2, JLG16.2 | 3 |
| *Mycena* | BTNR2.1 | | 1 |

| | | | |
|---|---|---|---|
| *Peniophorella* | SBS1.2, NIE6.2, NIE7.2, NIE8.1, BTNR4.1, LNR12.1, WCP6.1, WCP8.1, WCP10.1, JGHB1.1, JGHB2.1, JGHB3.1 | BTNR9.1, LNR10.1 | 7 |
| *Perenniporia* | SBS4.2, LNR9.1 | BTNR8.2 | 3 |
| *Phanerochaete* | NYL1.1, NYL1.2, LNR11.1 | LNR1.1 | 2 |
| *Phlebiopsis* | | JLG1.4 | 1 |
| *Picipes* | NS1.3 | | 1 |
| *Punctulariopsis* | SBS3.2, LNR8.1 | | 2 |
| *Scytinostroma* | SZ4.1 | | 1 |
| *Serendipita* | NS8.1, GAIA4.1 | | 2 |
| *Stypella* | | JLG3.1 | 1 |
| *Subulicystidium* | NIE3.1, NIE3.2 | JLG1.2, JLG12.1, JLG14.1 | 2 |
| *Trametes* | | JLG2.2, JLG8.2, JLG18.2 | 1 |
| *Trichaptum* | NYL1.2, JGHA2.1, JGHA3.1, JGHA5.1, JGHA6.1, JGHA7.1, JGHA7.2, JGHA7.3, JGHA8.1, JGHA9.1, JGHA9.2, JGHA10.1, JGHA11.1, TBJG1.2 | | 3 |
| *Tulasnella* | | JLG12.1, JLG20.1 | 1 |
| *Vuilleminia* | LNR11.1, LNR11.2, TBJG7.1 | JLG11.1 | 3 |
| *Xylodon* | SZ1.1, WCP7.1 | LNR10.1 | 3 |
| ***Mucoromycota*** | | | |
| **Genus** | **Processed Wood** | **Natural Log** | **No. of locations** |
| *Rhizopus* | WCP4.2 | | 1 |

**Table S5.** Widely distributed dominant ascomycete genera, their sampling locations, and reported ecological roles.

| Genus | Locations appeared | Ecological Role | Rot Type | Isolates |
|---|---|---|---|---|
| ***Chalarosphaeria*** | CC, WCP, BTNR, TBJG | Saprotrophic on dead plant tissues, frequently isolated from dead wood and bark of broad-leaved tree species [85]. | Not well studied | None |
| ***Cladophialophora*** | SZ, NS, WCP, TBJG, LNR, JLG | Known for human infections, some species associated with living plants (corn and other vegetables). Found | Not well studied | MOE119B-2, MOE126A-2, MOE127B-1-1, MOE130B-1 |

| | | | | |
|---|---|---|---|---|
| | | occasionally in decaying wood [86]. Some species potentially involved in stone biodegradation [87,88]. | | |
| ***Exophiala*** | SBS, NIE, GAIA, NYL, LNR, CC, JLG | Widely distributed across diverse climates, with species occurring in both natural and anthropogenic environments, and some species known to cause infections in humans and animals. Found in decaying wood [89]. Considered a micro-colonial fungus that is involved in stone biodegradation. | Not well studied | MOE070A-2, MOE132A-2, MOE133A-2, MOE134A-2, MOE135B-1 |
| ***Pseudodactylaria*** | NS, LNR, TBJG, TBH | Saprotrophic on submerged decaying wood, occurring in both fresh water and terrestrial habitats [90]. | Not well studied | None |
| ***Rhexodenticula*** | SBS, SZ, NS, LNR | Found in rotten leaves and dead culms in terrestrial habitat, with some species also acting as saprotrophs on submerged decaying wood in fresh water [91]. | Not well studied | None |
| ***Sporidesmium*** | NIE, CC, TBJG, JLG | Saprotrophic on submerged decaying wood [92] and on newly or older fallen logs and reported across all stages of decomposition throughout the year [93]. Certain species, such as *Sporidesmium sclerotivorum,* are known as a biotrophic mycoparasite [94]. | Not well studied | None |
| ***Teratosphaericola*** | NIE, NS, NYL, WCP | Known to be foliicolous and plant pathogenic on Eucalyptus [95]. | Not well studied | None |
| ***Trichoderma*** | NIE, WCP, LNR, JLG | *T. hamatum*, *T. harzianum* and *T. koningii* caused significant mass loss in decayed beech wood, but rarely in sound wood [95], Saprotrophic on decaying wood [96], opportunistic on plants and a known biocontrol agent of plant pathogen [97]. | Able to produce cellulase and xylanases [96]. | Multiple plates were thrown as it was contaminating our pure isolates. |

Bold text denotes table headings.

*S3.2 Beta diversity heatmaps all areas (S1)*

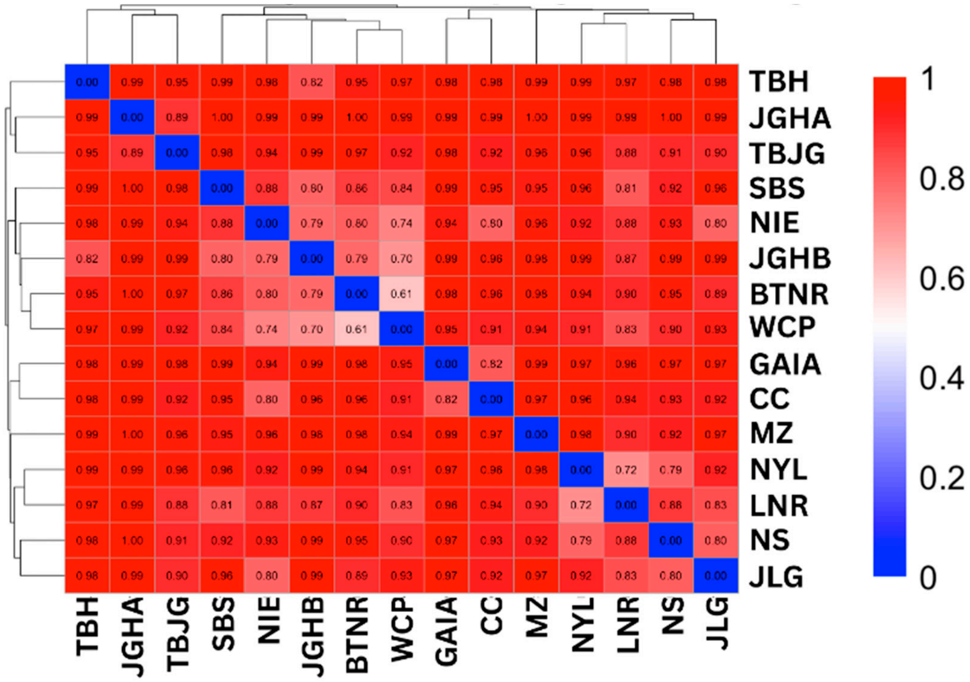


**Figure S1.** Beta diversity heatmap showing Bray-Curtis dissimilarity between fungal communities at a genus level across all locations (excluding inconclusive genera).

*S3.3 Beta diversity heatmaps by area (S2 – S16)*

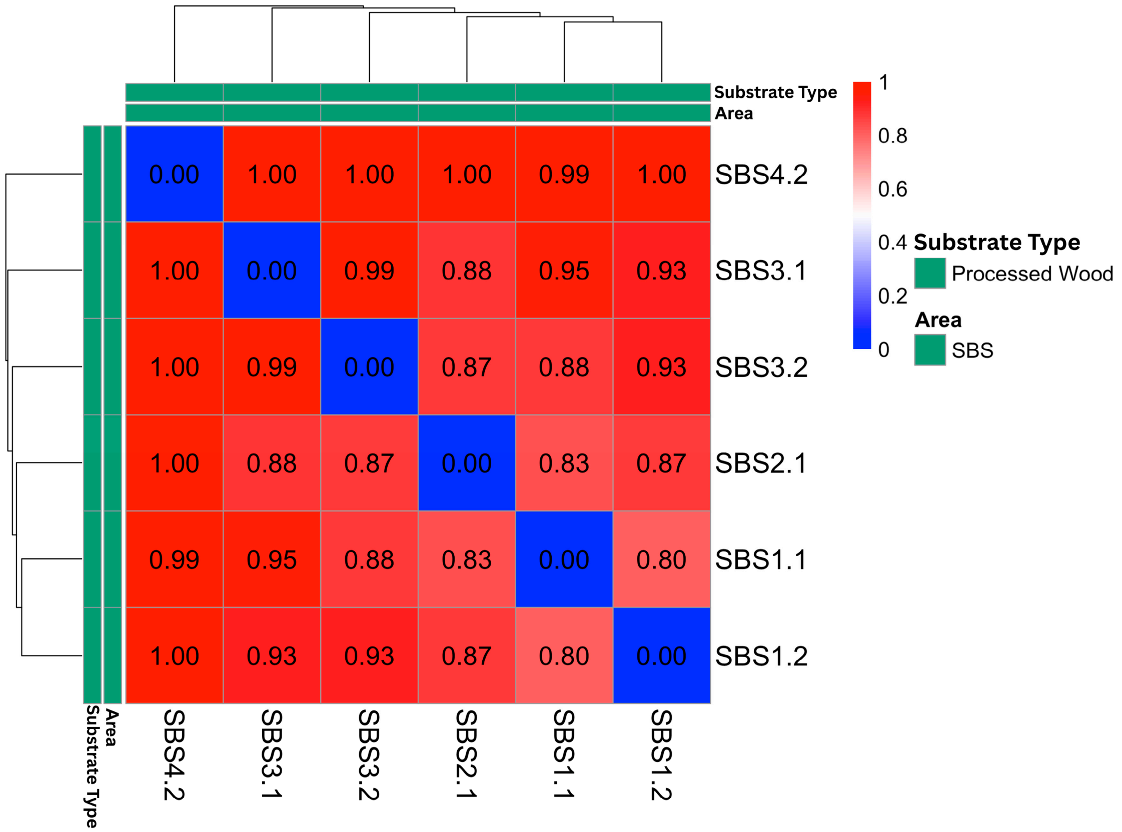


**Figure S2.** Beta diversity heatmap showing Bray-Curtis dissimilarity between fungal communities at a genus level across all samples in SBS

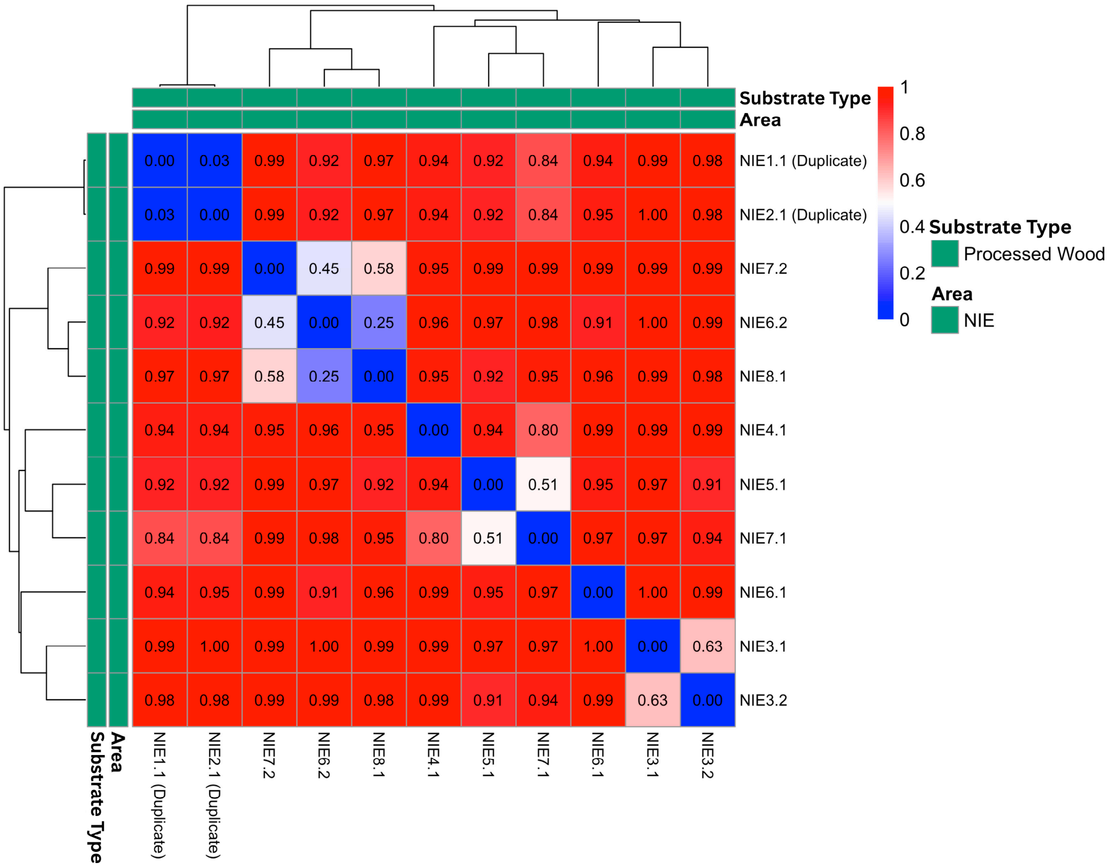


**Figure S3.** Beta diversity heatmap showing Bray-Curtis dissimilarity between fungal communities at a genus level across all samples in NIE

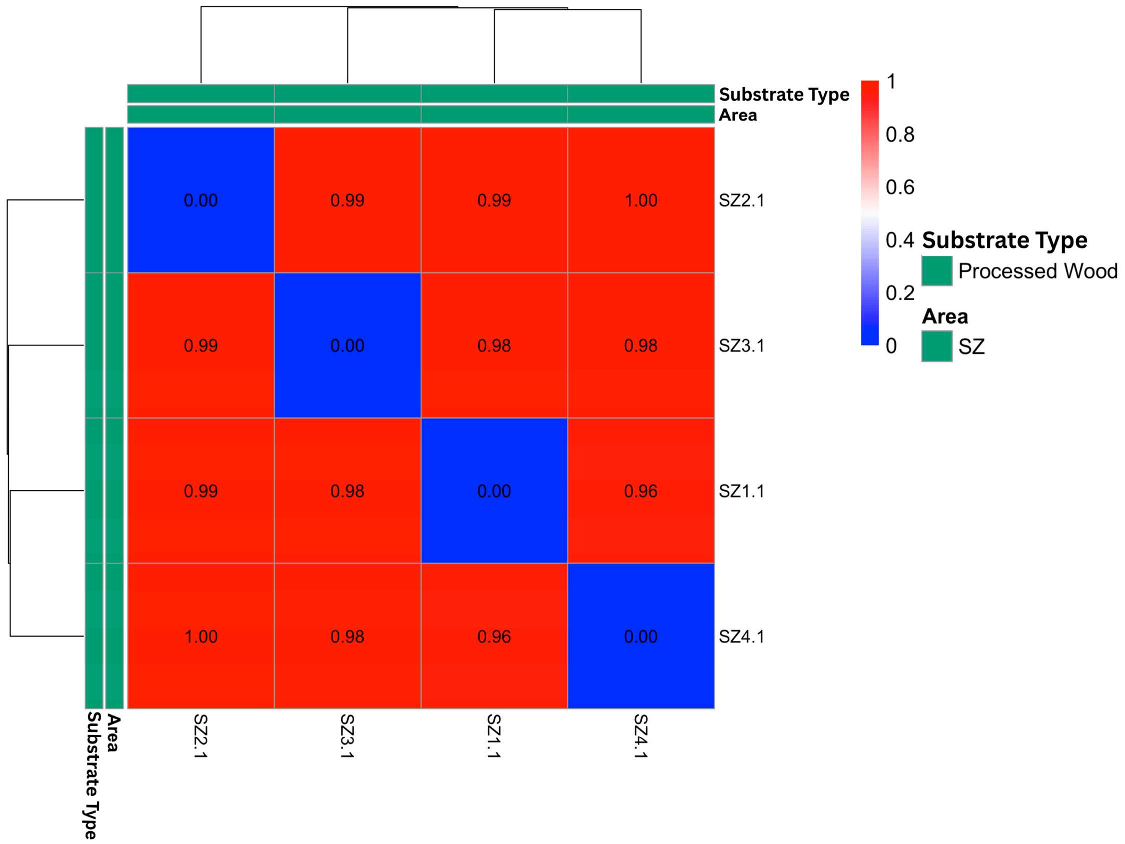


**Figure S4.** Beta diversity heatmap showing Bray-Curtis dissimilarity between fungal communities at a genus level across all samples in SZ

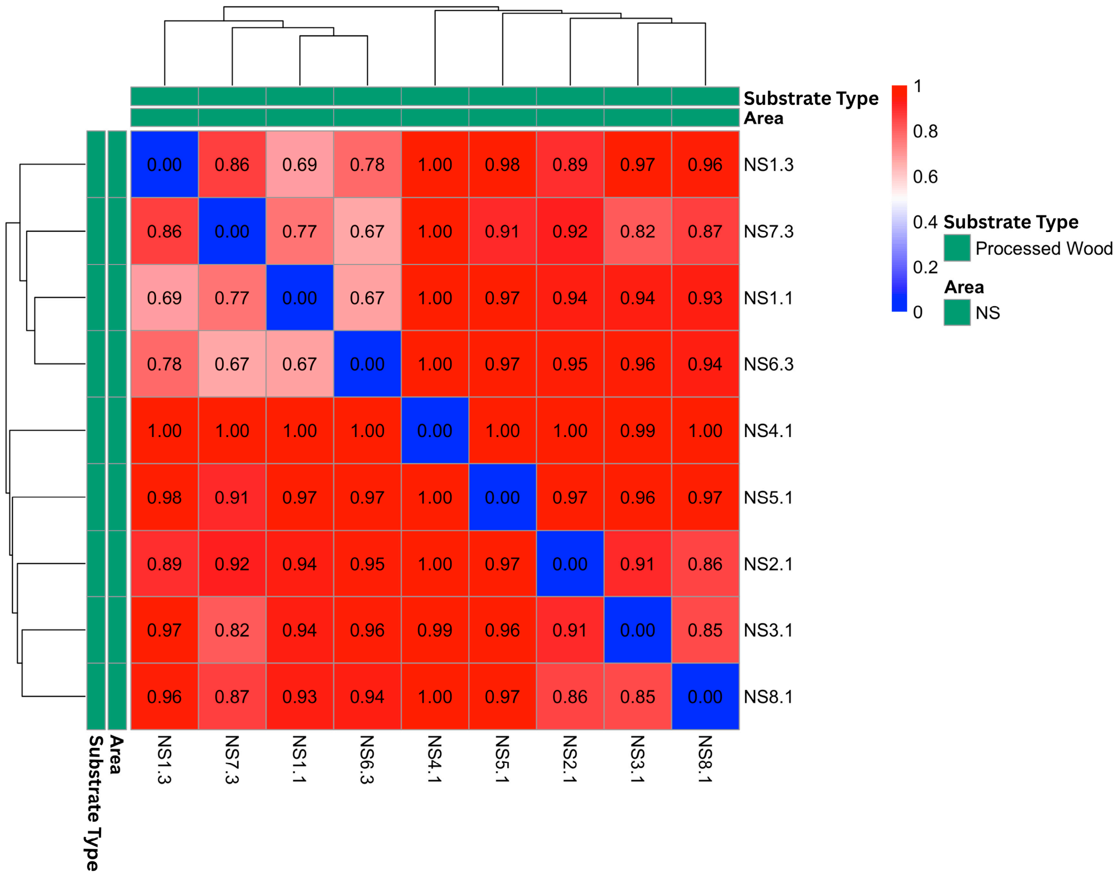


**Figure S5.** Beta diversity heatmap showing Bray-Curtis dissimilarity between fungal communities at a genus level across all samples in NS

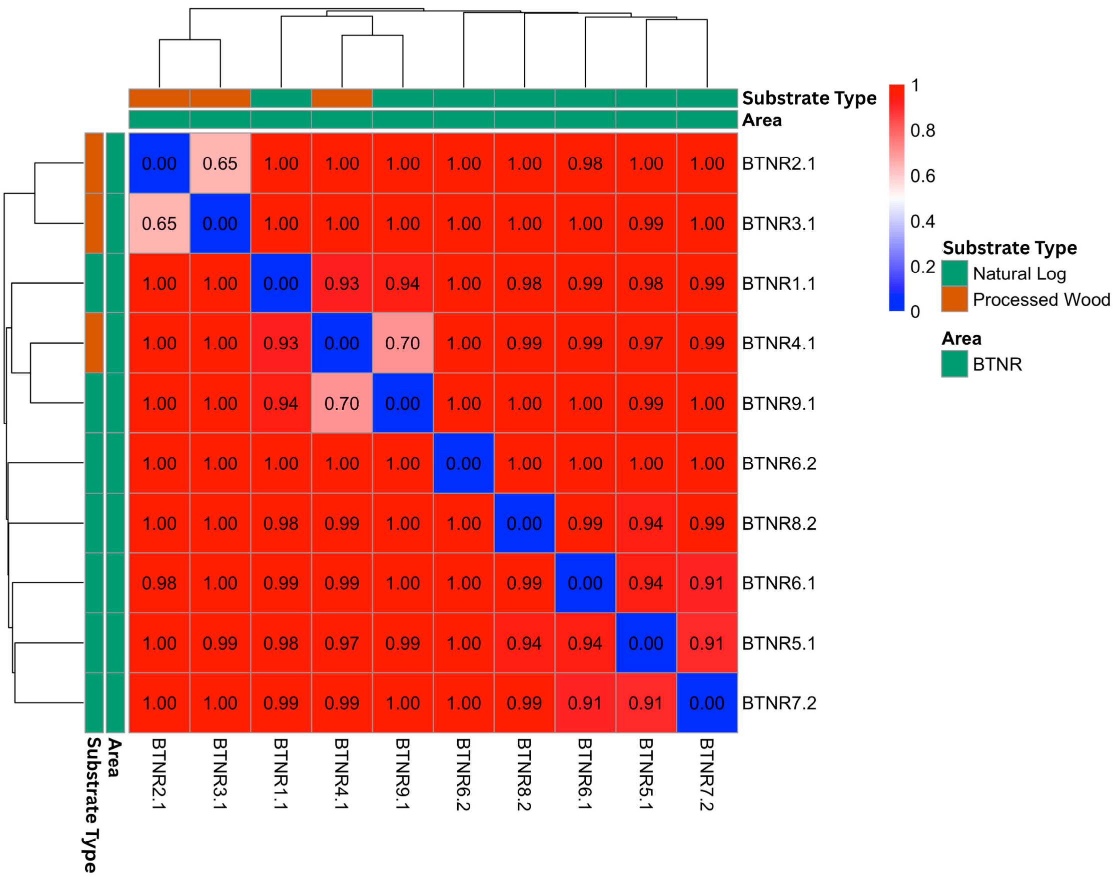


**Figure S6.** Beta diversity heatmap showing Bray-Curtis dissimilarity between fungal communities at a genus level across all samples in BTNR

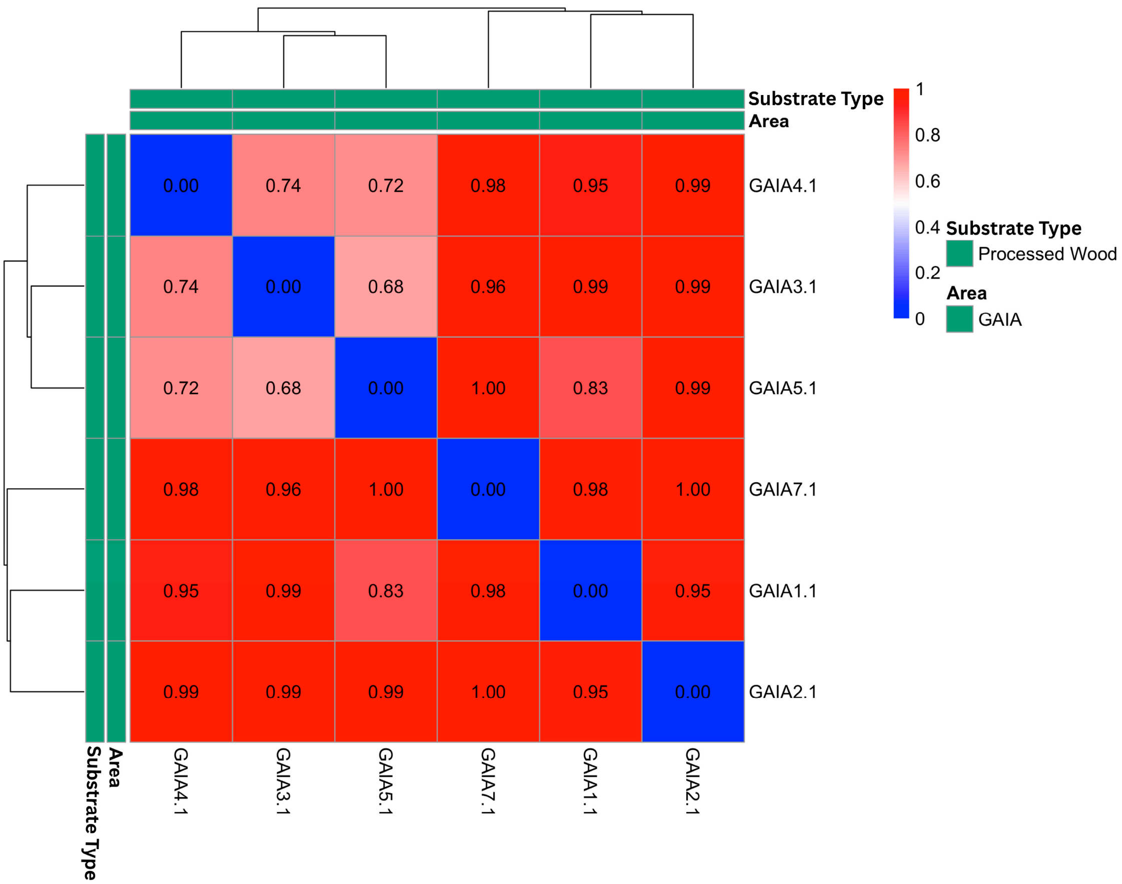


**Figure S7.** Beta diversity heatmap showing Bray-Curtis dissimilarity between fungal communities at a genus level across all samples in GAIA

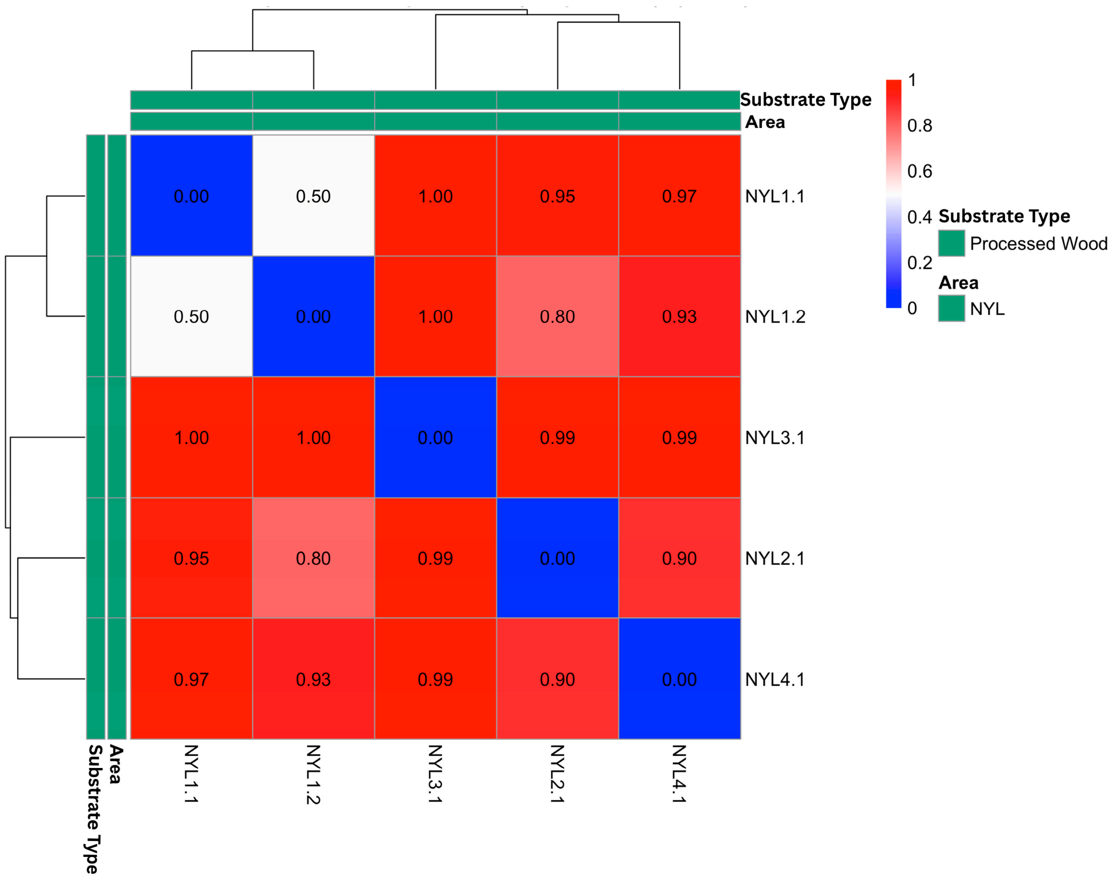


**Figure S8.** Beta diversity heatmap showing Bray-Curtis dissimilarity between fungal communities at a genus level across all samples in NYL

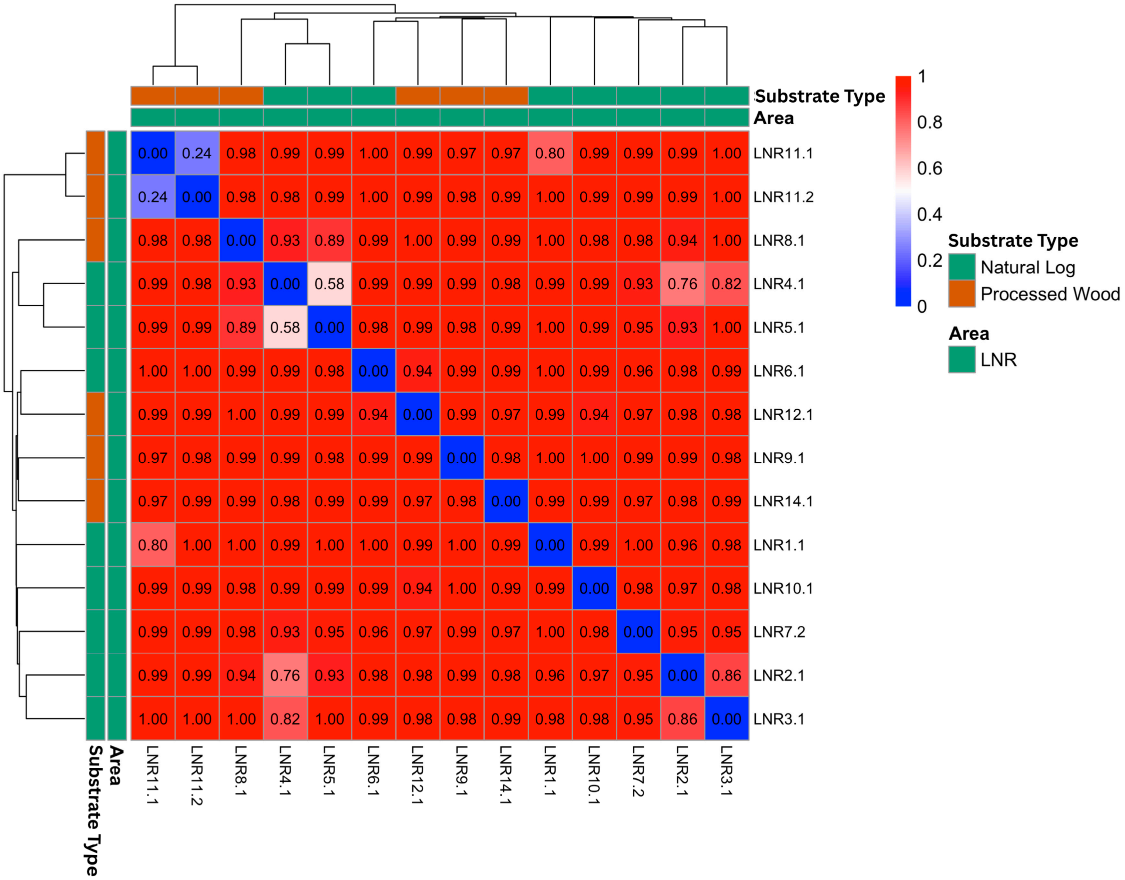


**Figure S9.** Beta diversity heatmap showing Bray-Curtis dissimilarity between fungal communities at a genus level across all samples in LNR

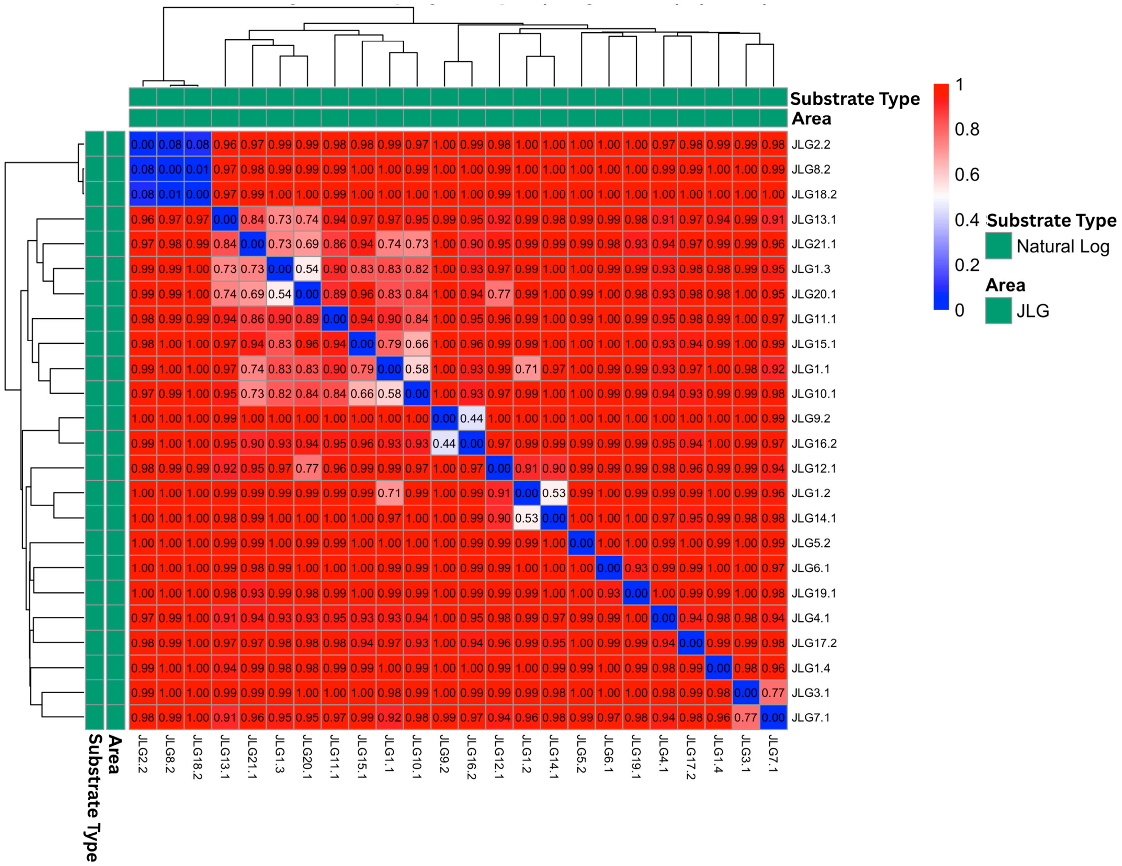


**Figure S10.** Beta diversity heatmap showing Bray-Curtis dissimilarity between fungal communities at a genus level across all samples in JLG

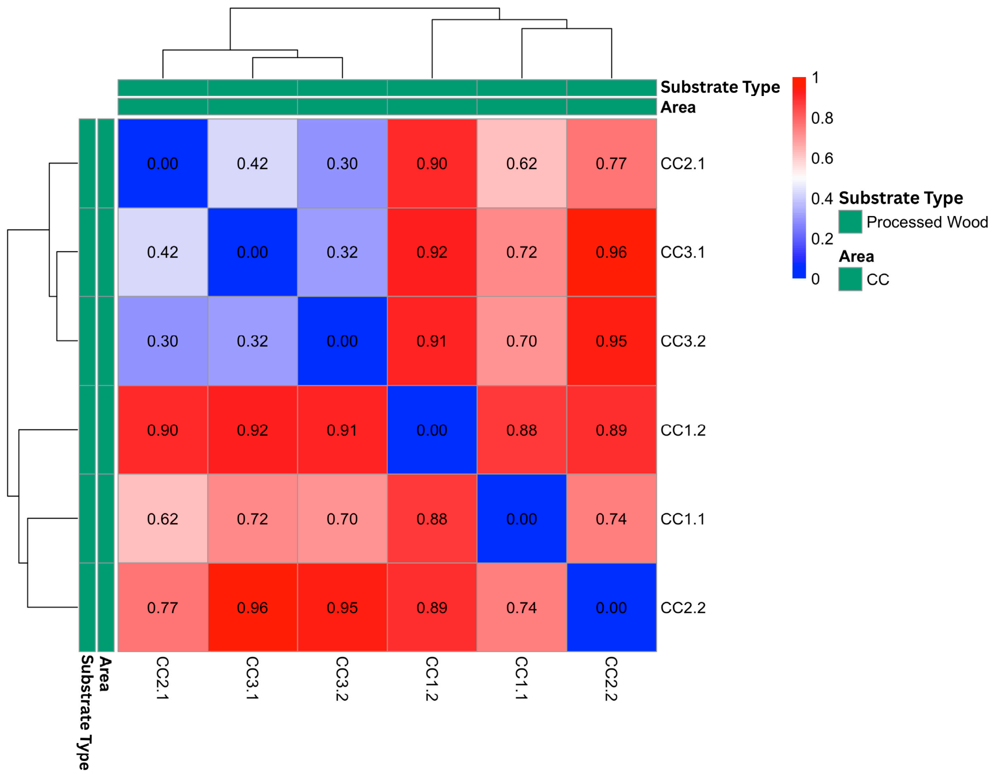


**Figure S11.** Beta diversity heatmap showing Bray-Curtis dissimilarity between fungal communities at a genus level across all samples in CC

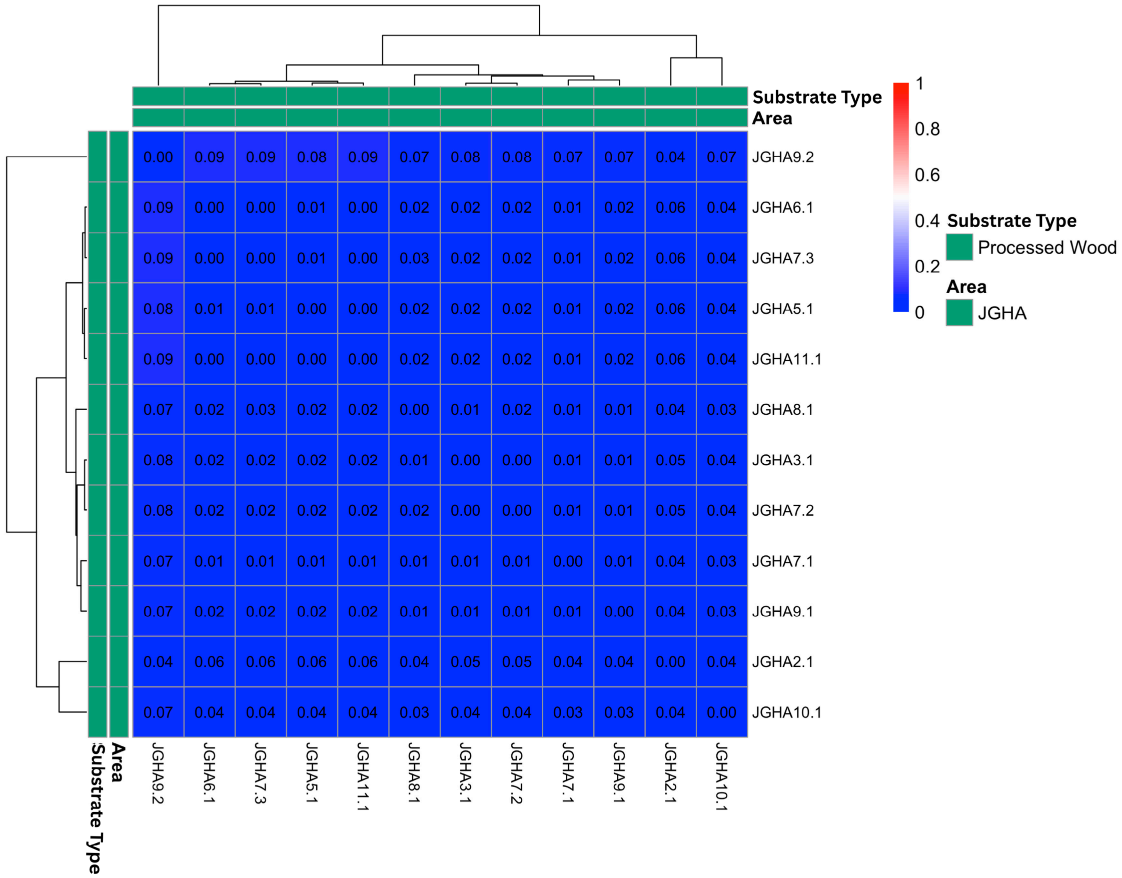


**Figure S12.** Beta diversity heatmap showing Bray-Curtis dissimilarity between fungal communities at a genus level across all samples in JGHA

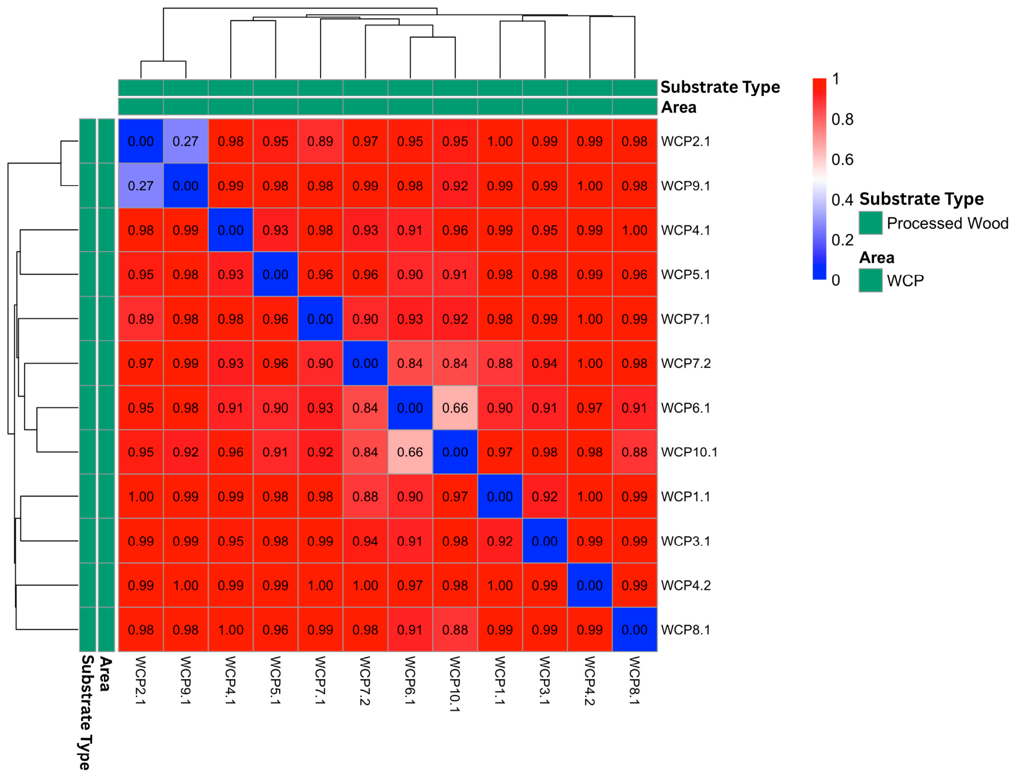


**Figure S13.** Beta diversity heatmap showing Bray-Curtis dissimilarity between fungal communities at a genus level across all samples in WCP

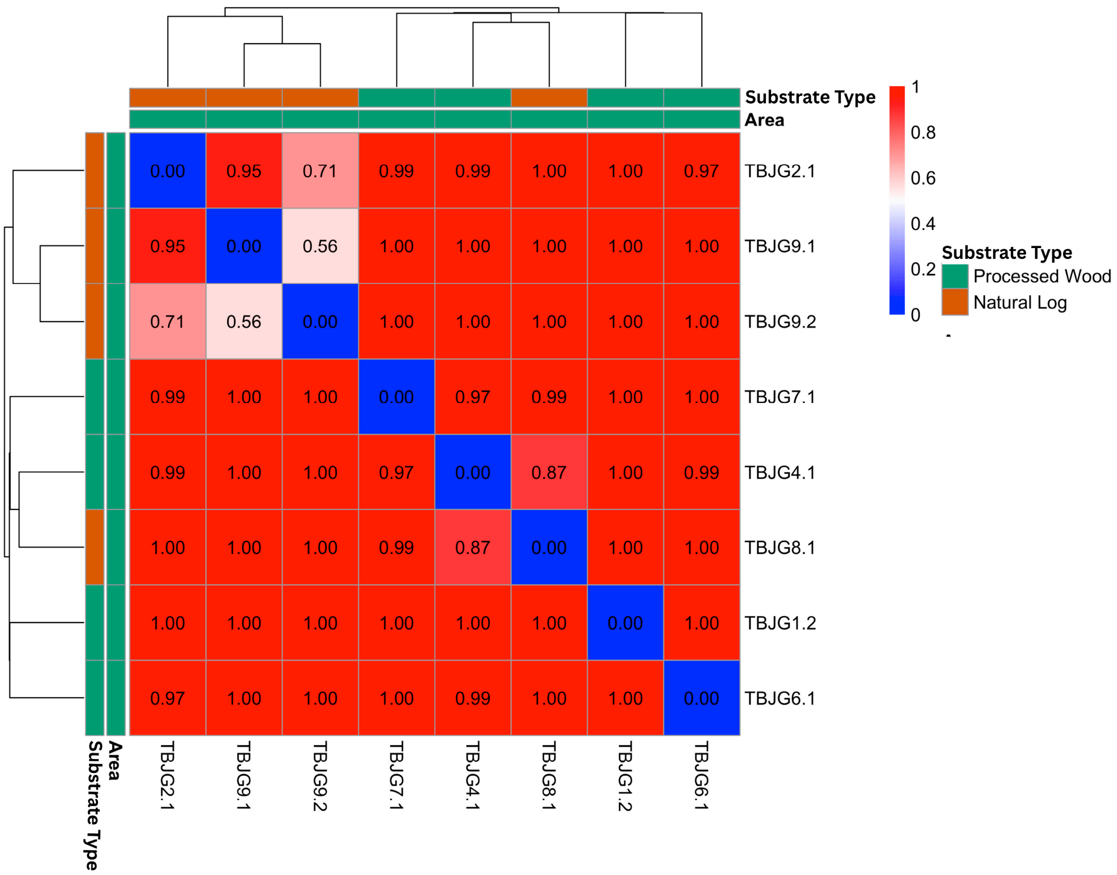


**Figure S14.** Beta diversity heatmap showing Bray-Curtis dissimilarity between fungal communities at a genus level across all samples in TBJG

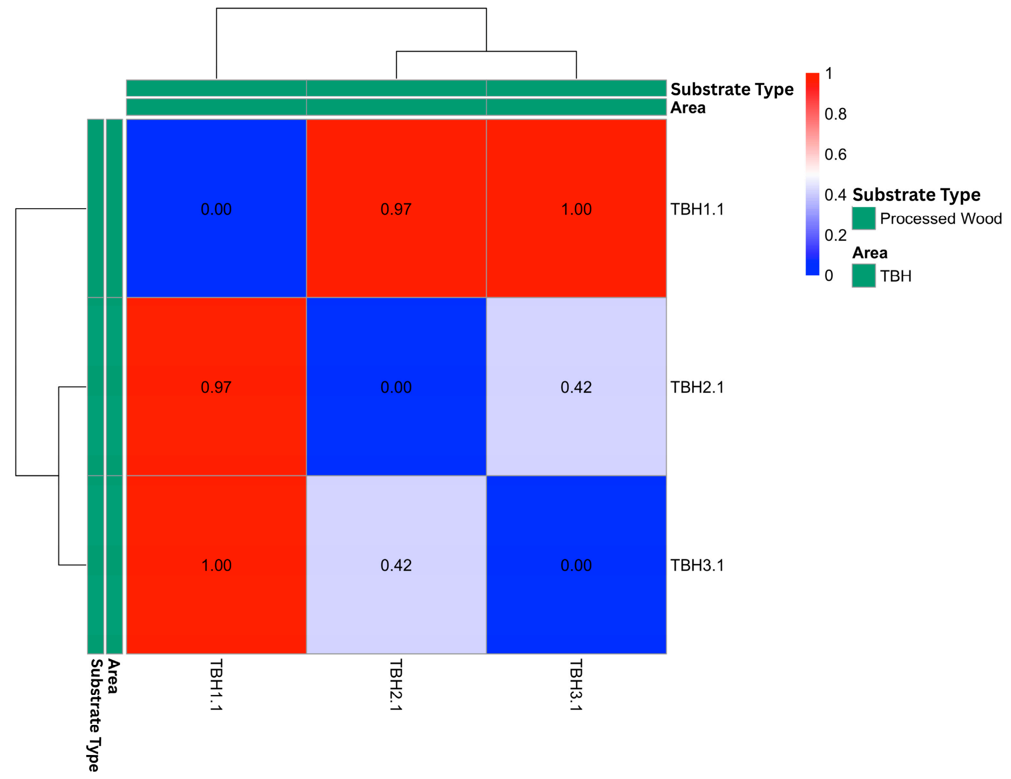


**Figure S15.** Beta diversity heatmap showing Bray-Curtis dissimilarity between fungal communities at a genus level across all samples in TBH

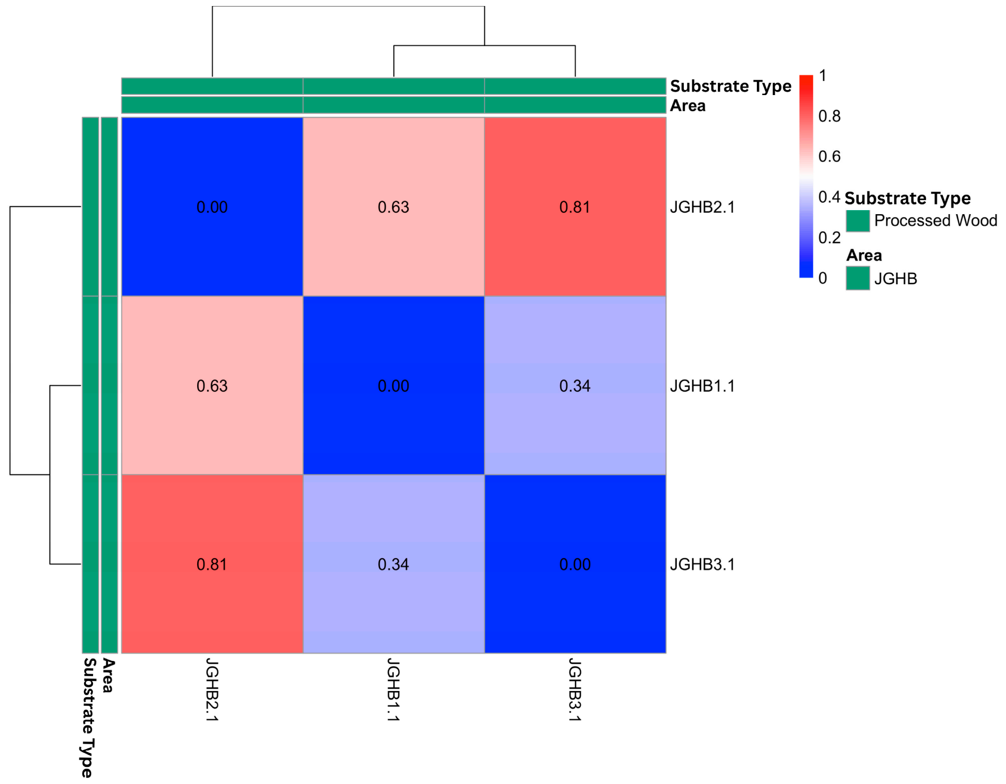


**Figure S16.** Beta diversity heatmap showing Bray-Curtis dissimilarity between fungal communities at a genus level across all samples in JGHB

*S3.4 Beta diversity heatmap for BTNR and WCP (S17)*

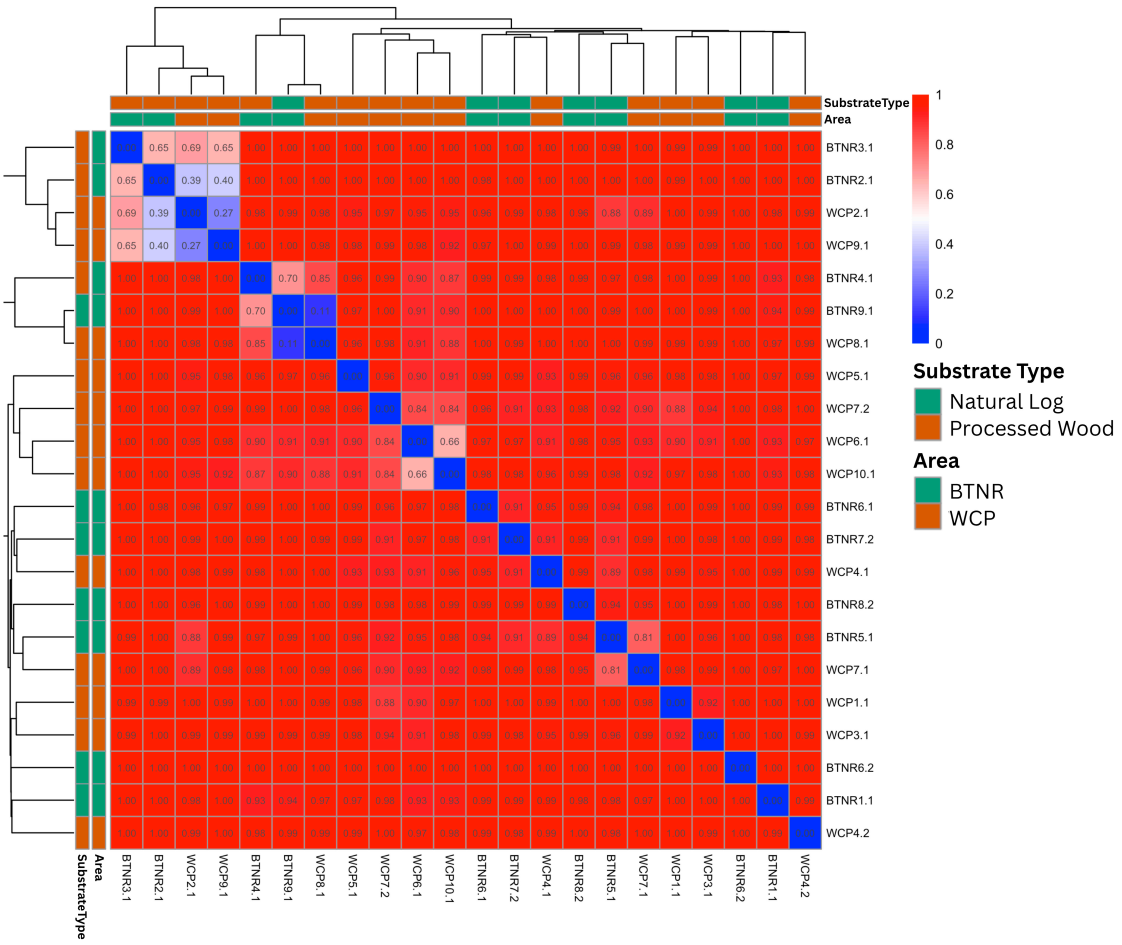


**Figure S17.** Beta diversity heatmap showing Bray-Curtis dissimilarity between fungal communities at a genus level in samples from West Coast Park and Bukit Merah Nature Reserve (excluding inconclusive genera).

*S3.5. Bar plot by area with genus > 1% (S18-32)*

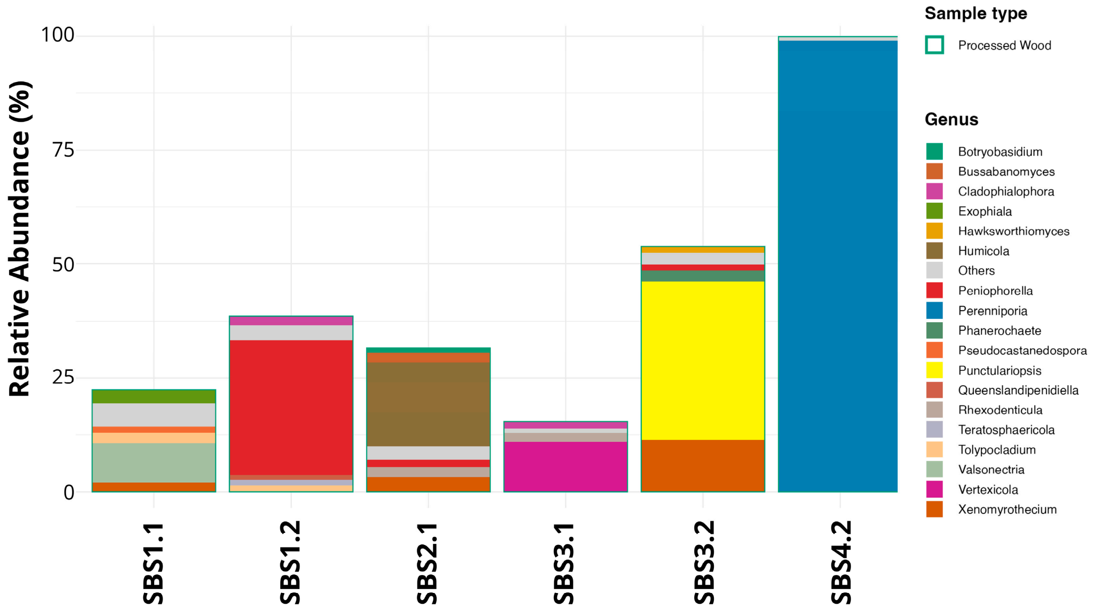


**Figure S18.** Bar plot of genera with > 1% relative abundance in SBS samples

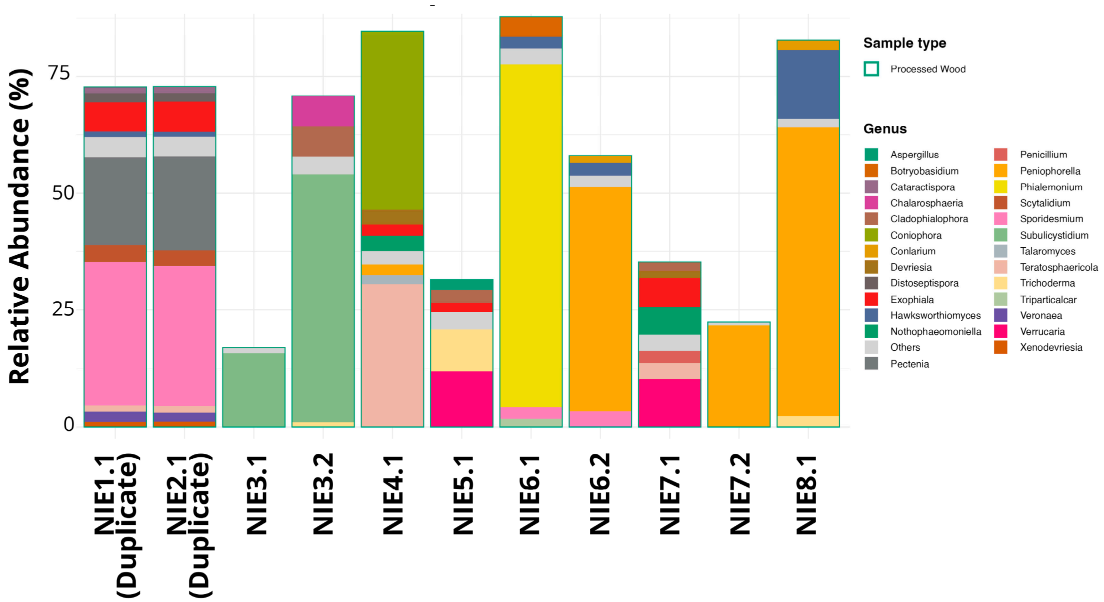


**Figure S19.** Bar plot of genera with > 1% relative abundance in NIE samples

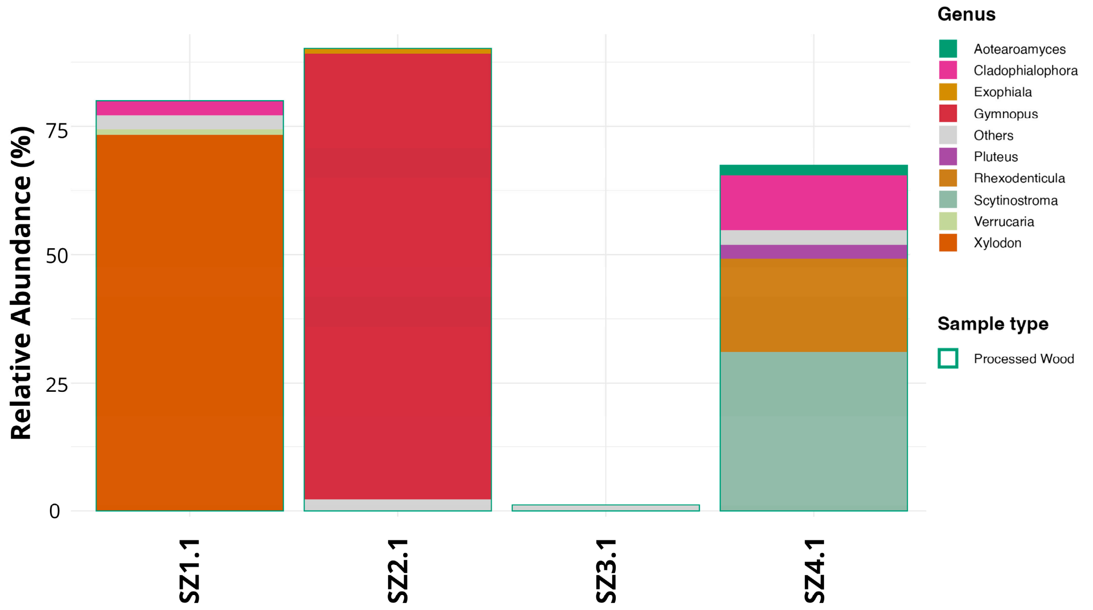


**Figure S20.** Bar plot of genera with > 1% relative abundance in SZ samples

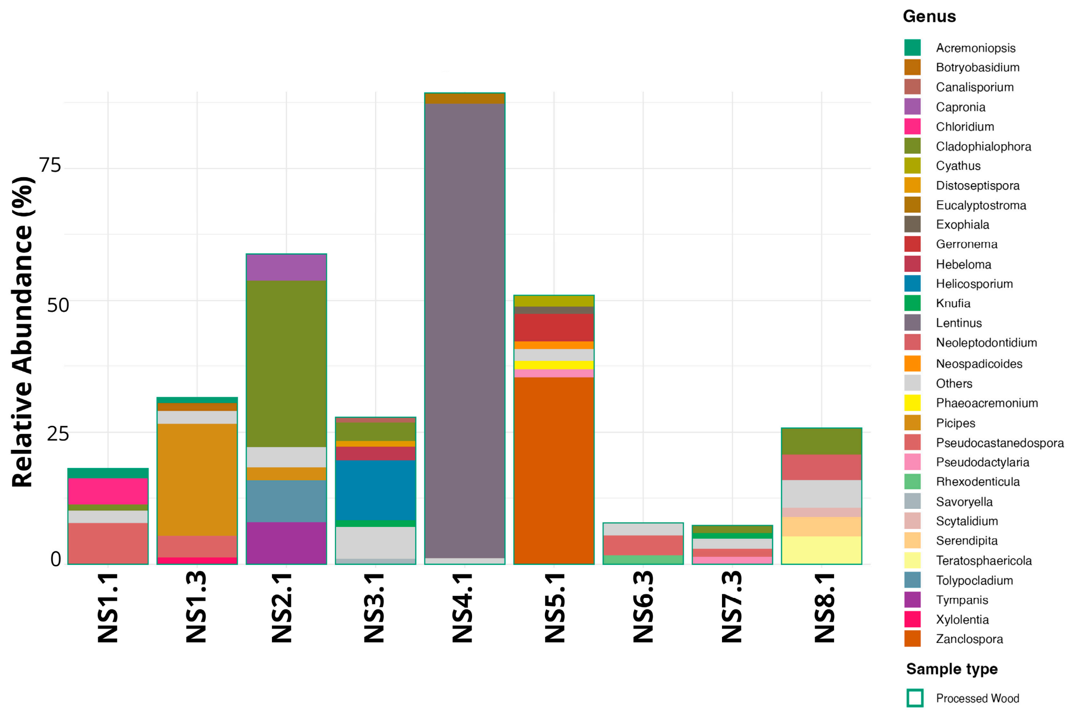


**Figure S21.** Bar plot of genera with > 1% relative abundance in NS samples

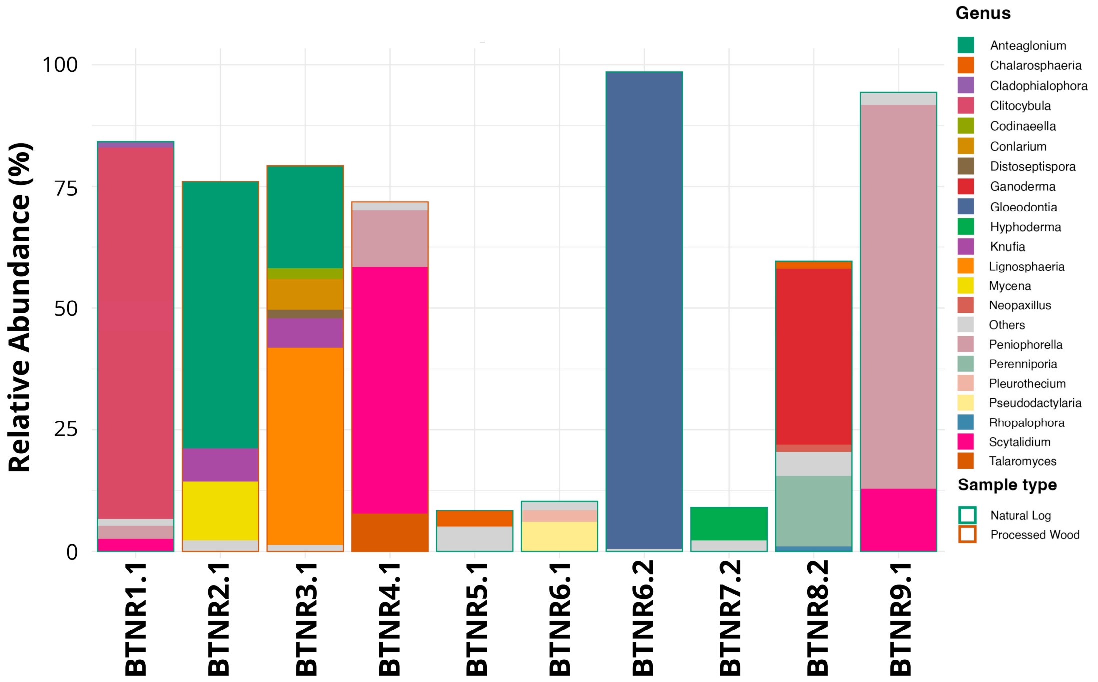


**Figure S22.** Bar plot of genera with > 1% relative abundance in BTNR samples

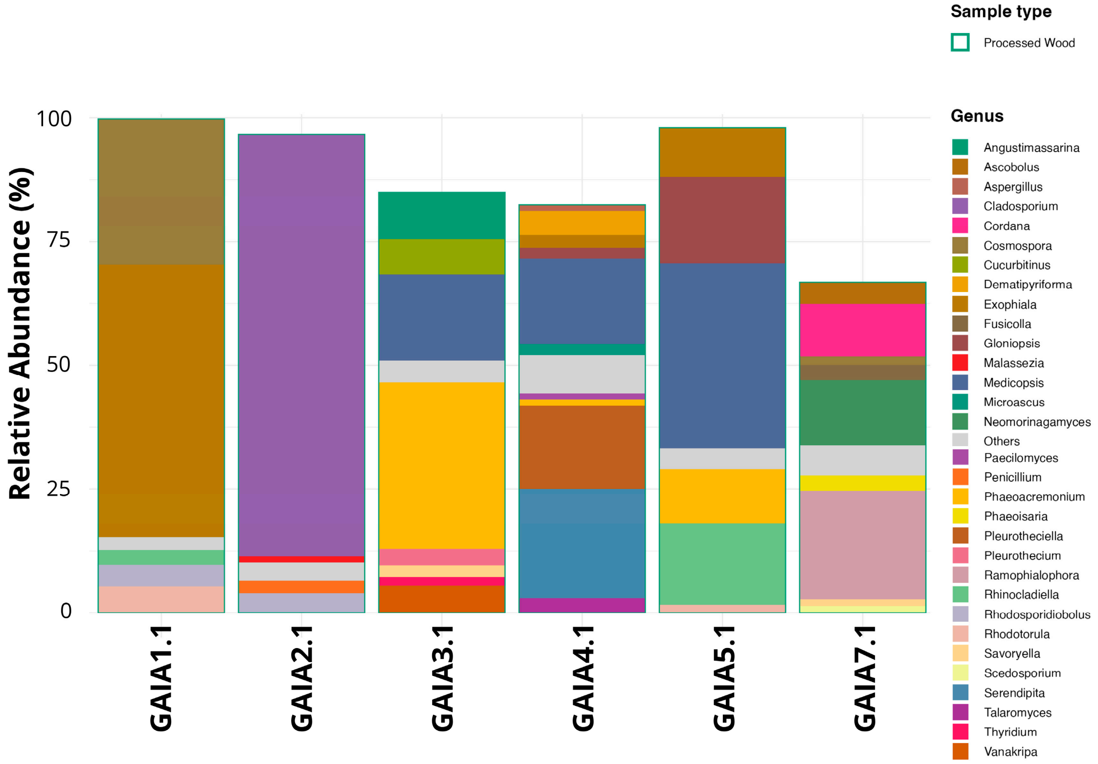


**Figure S23.** Bar plot of genera with > 1% relative abundance in GAIA samples.

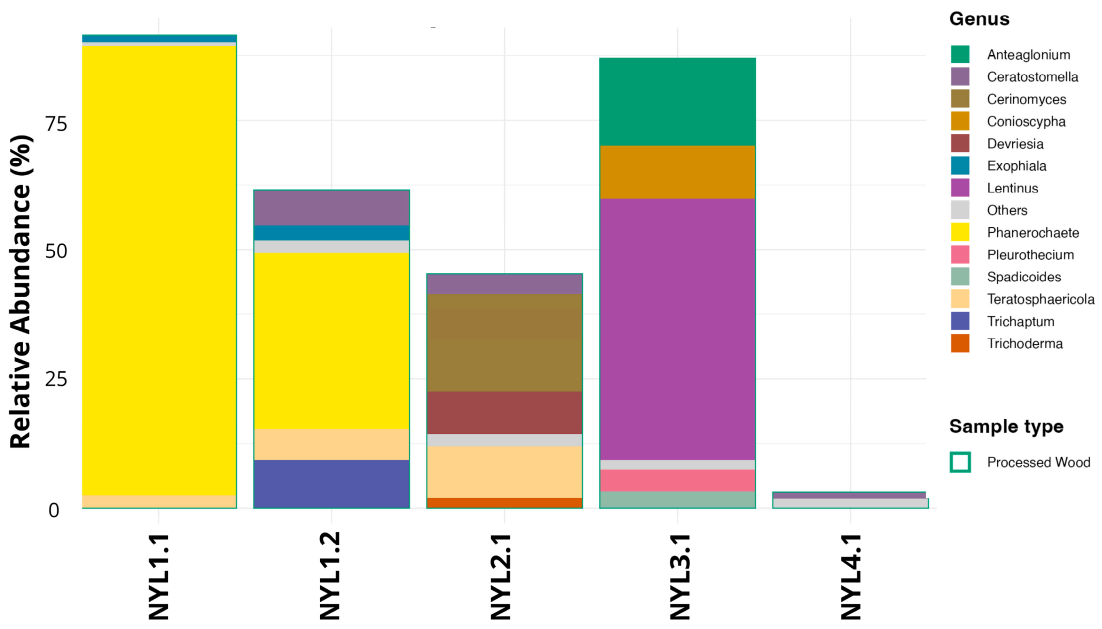


**Figure S24**. Bar plot of genera with > 1% relative abundance in NYL samples

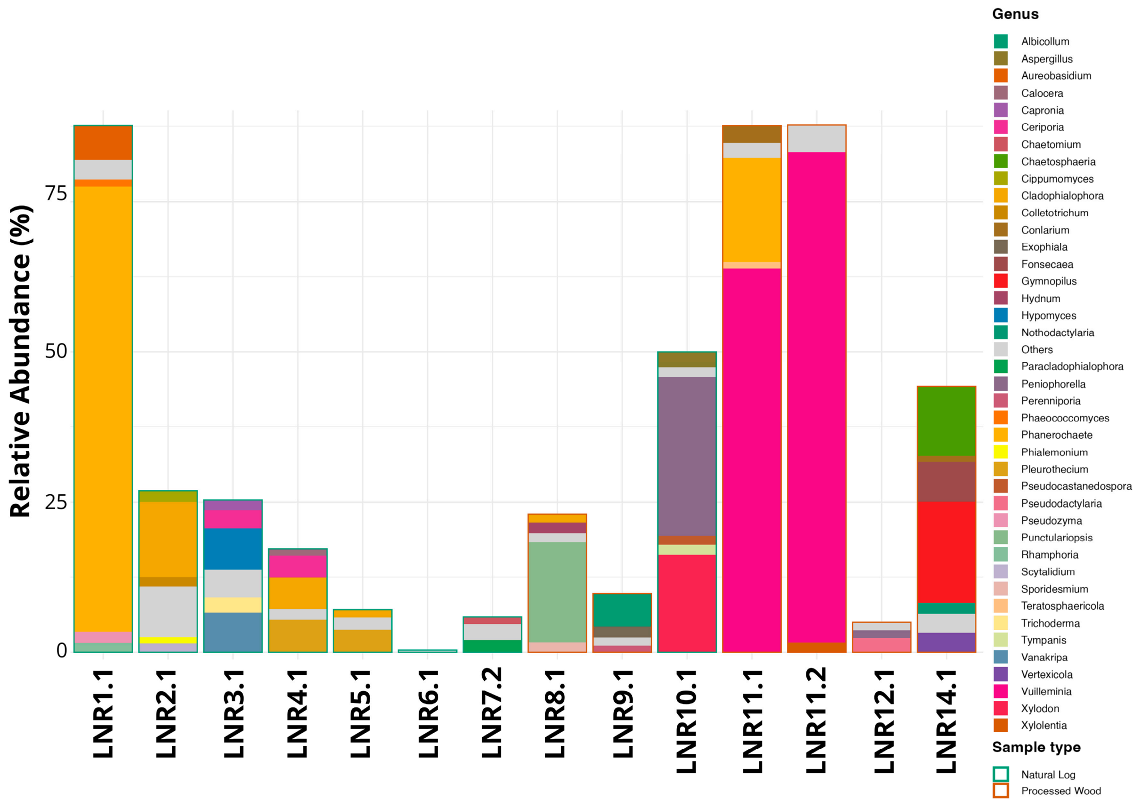


**Figure S25**. Bar plot of genera with > 1% relative abundance in LNR samples

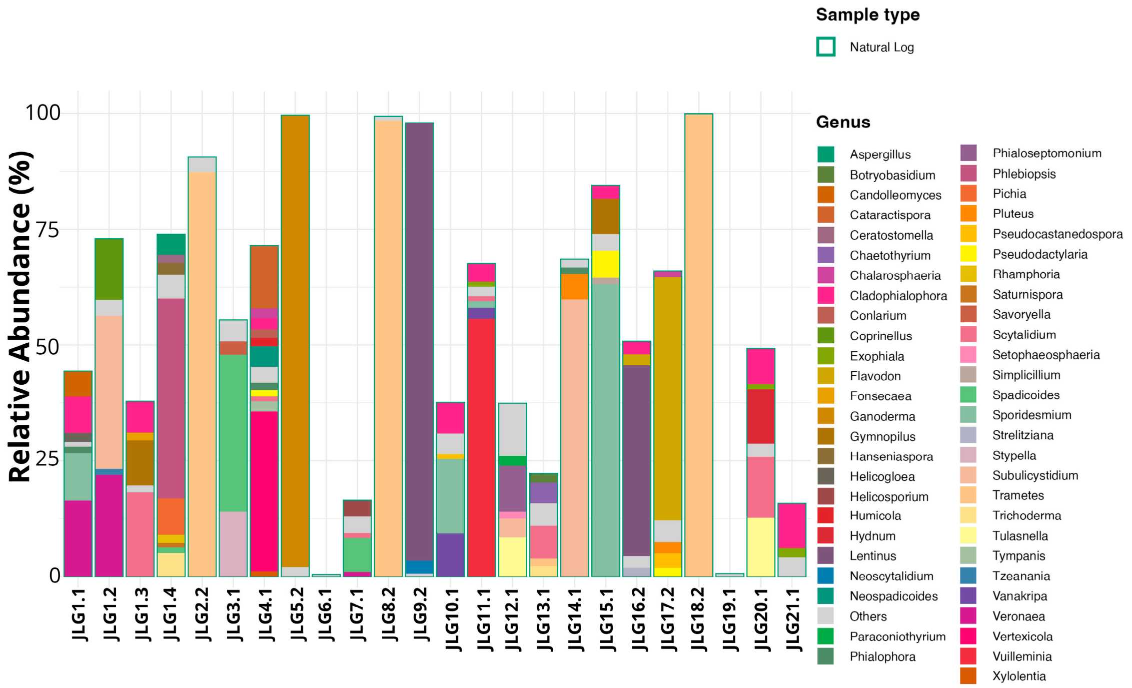


**Figure S26**. Bar plot of genera with > 1% relative abundance in JLG samples

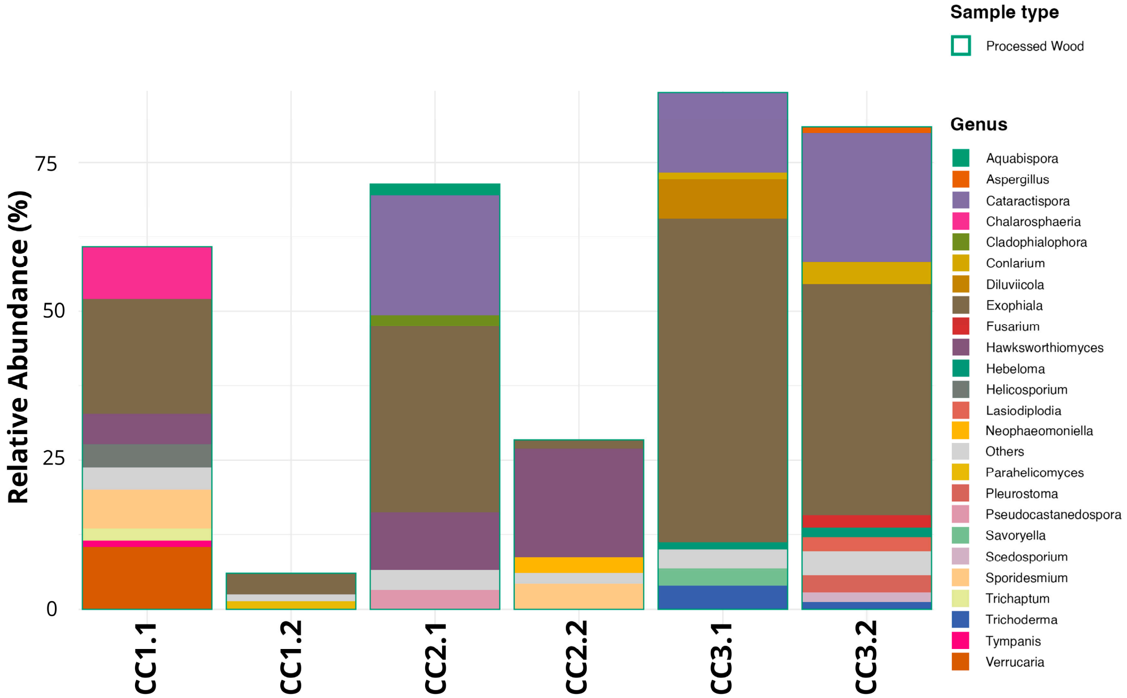


**Figure S27**. Bar plot of genera with > 1% relative abundance in CC samples

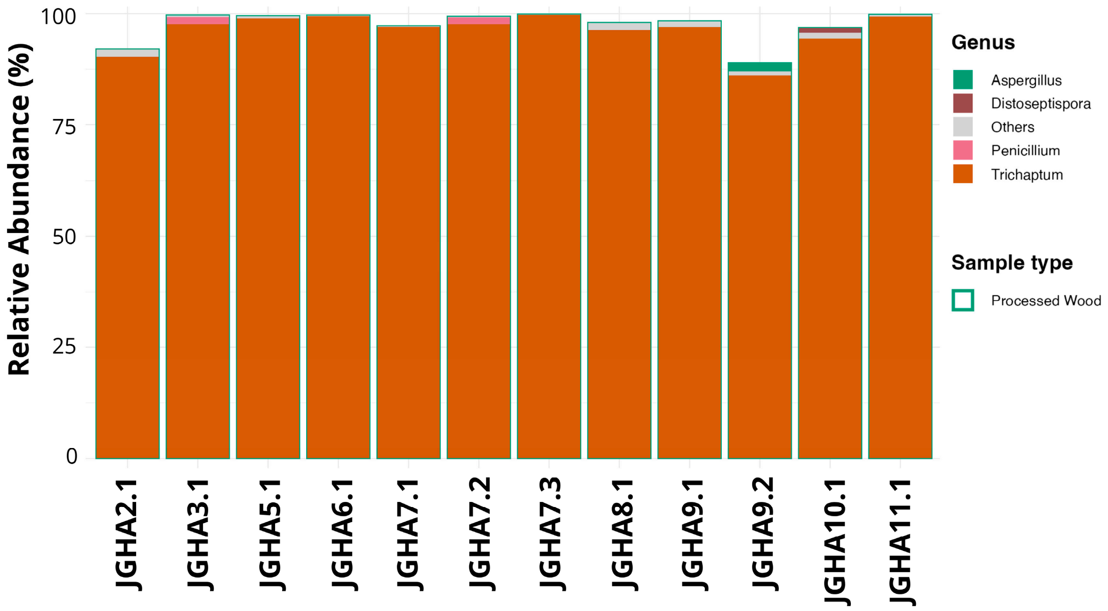


**Figure S28**. Bar plot of genera with > 1% relative abundance in JGHA samples

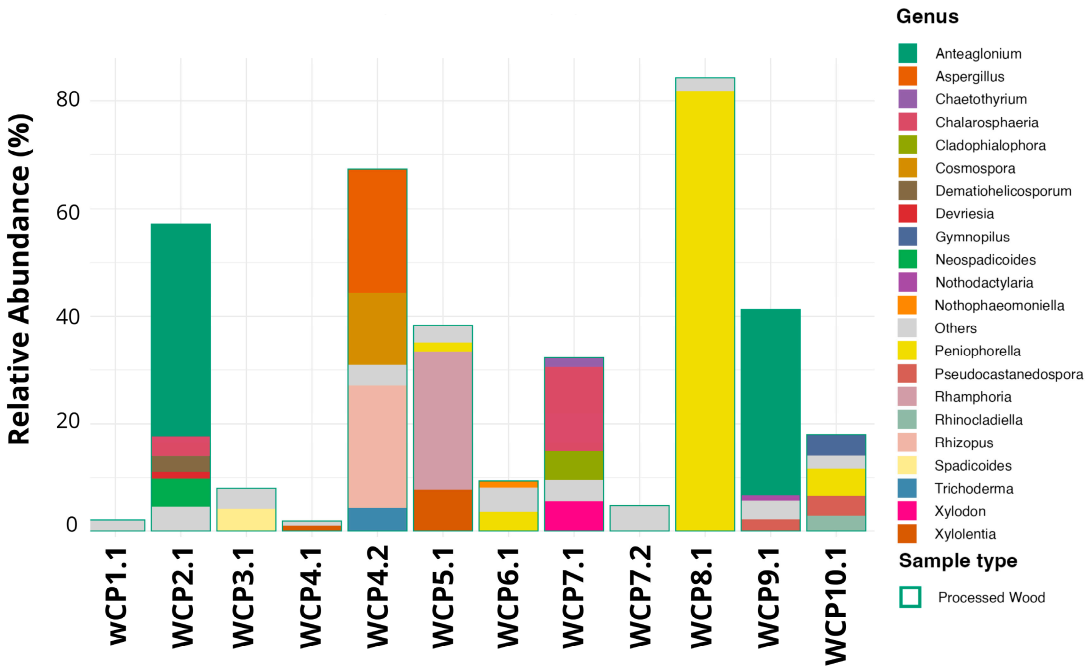


**Figure S29**. Bar plot of genera with > 1% relative abundance in WCP samples

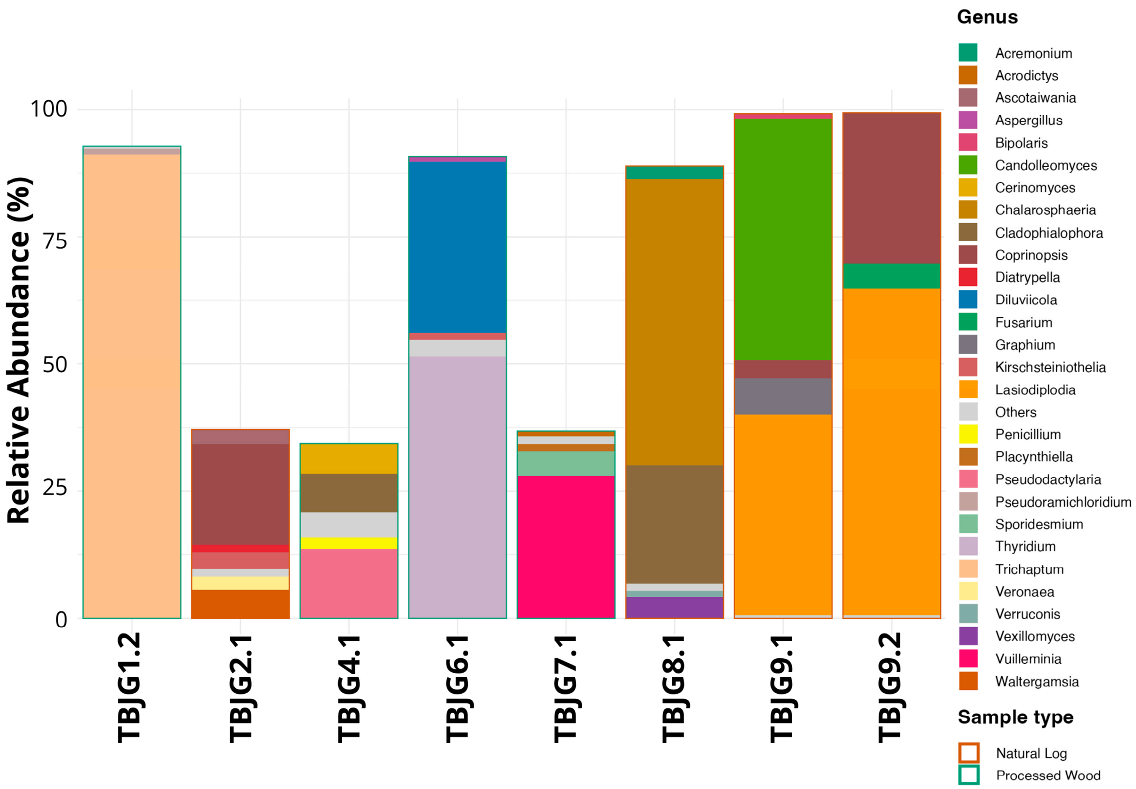

**Figure S30**. Bar plot of genera with > 1% relative abundance in TBJG samples

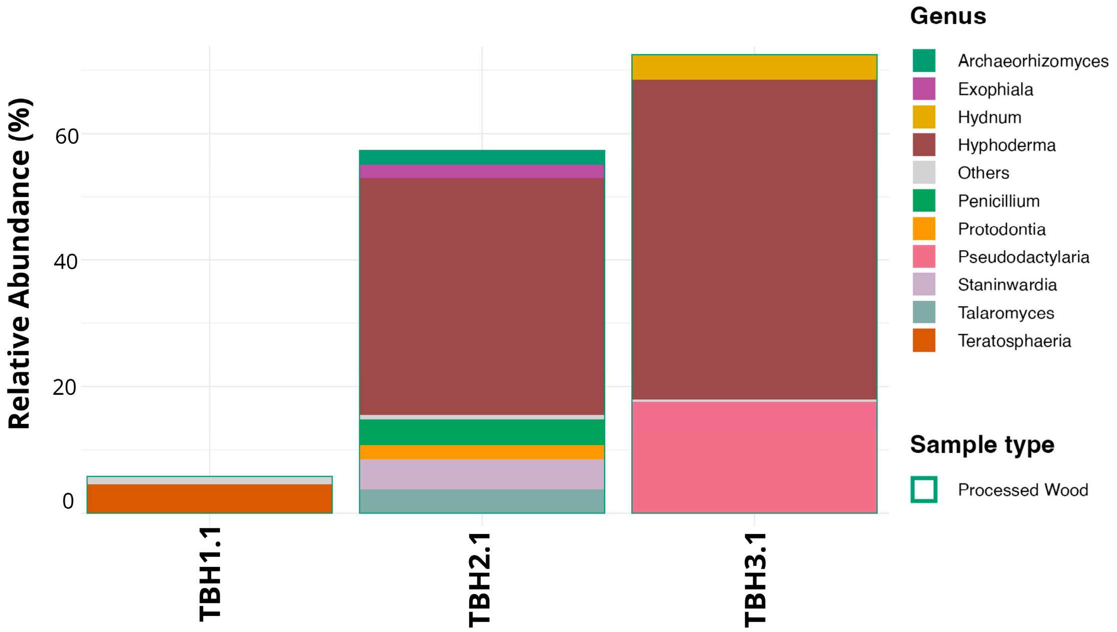

**Figure S31**. Bar plot of genera with > 1% relative abundance in TBH samples

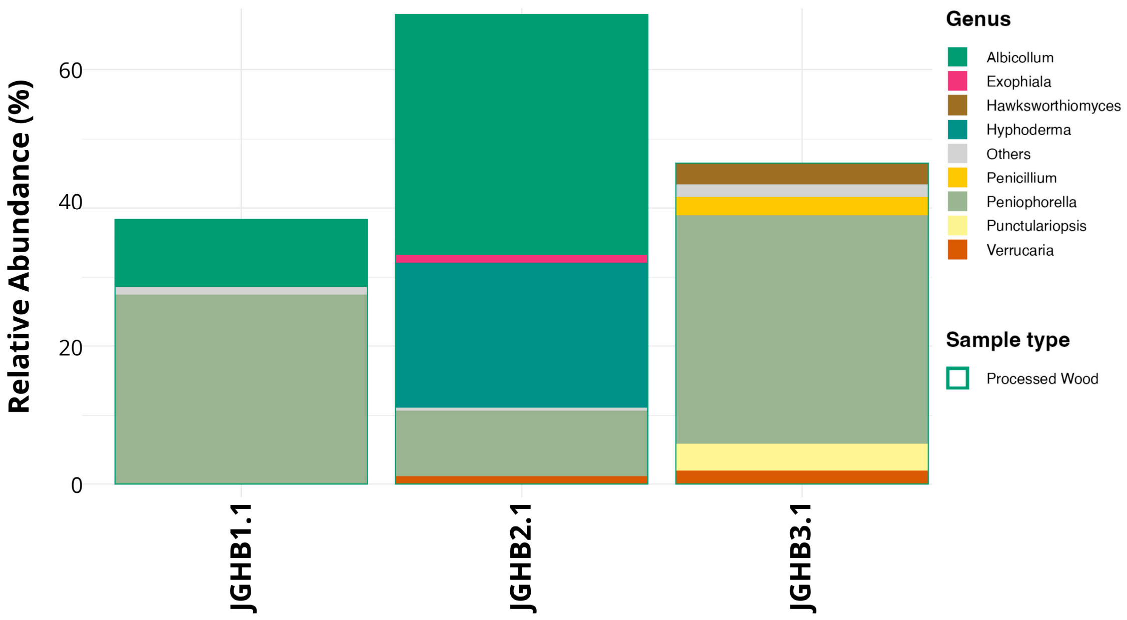


**Figure S32**. Bar plot of genera with > 1% relative abundance in JGHB samples

*S3.6. NMDS ordination*

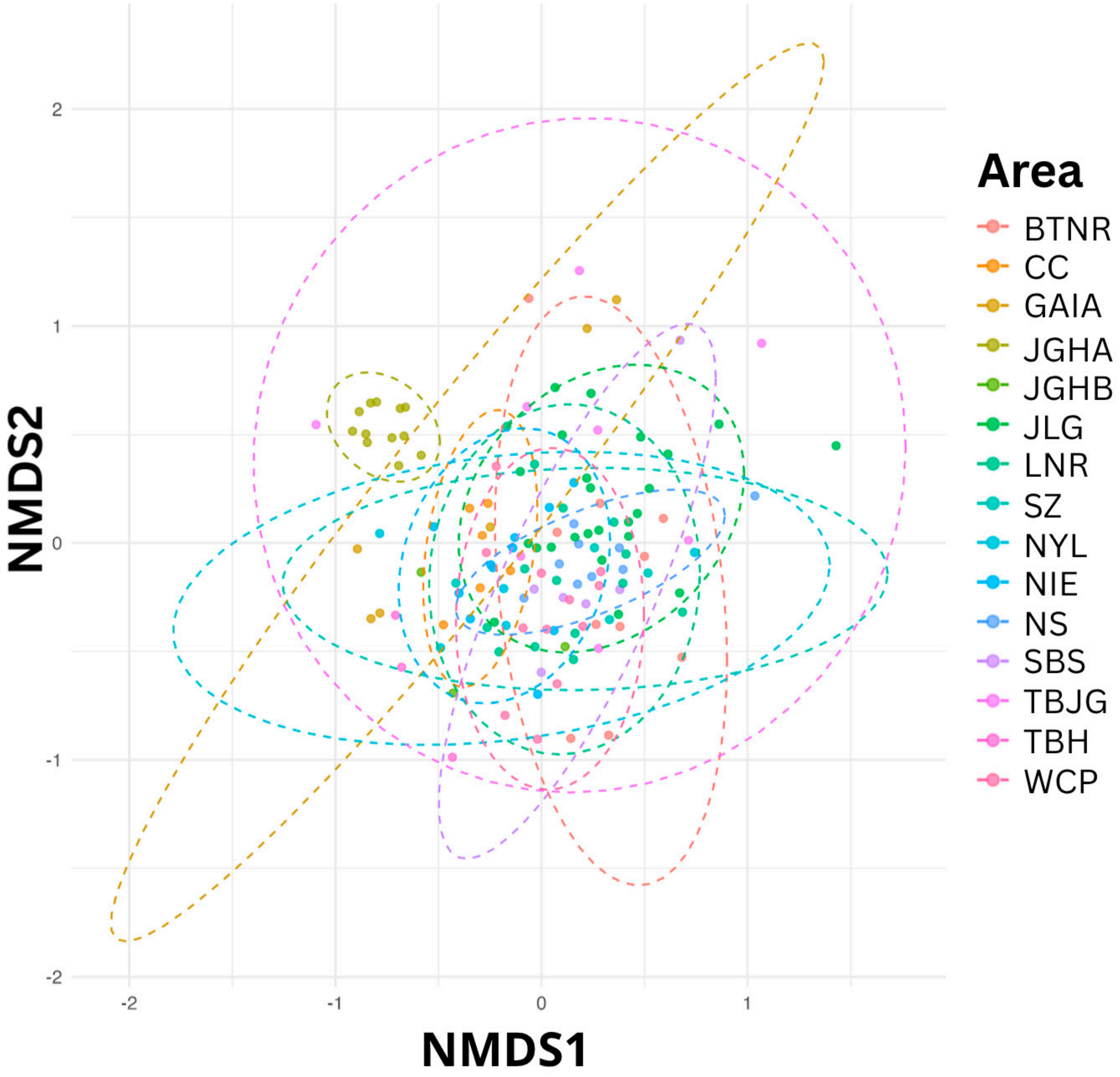


**Figure S33.** NMDS ordination of fungal community composition based on Bray-Curtis dissimilarity for all wood samples across 15 locations in Singapore (stress = 0.273). Ellipses represent 95% confidence intervals for each location.
